# Exponentially tighter bounds on limitations of quantum error mitigation

**DOI:** 10.1038/s41567-024-02536-7

**Published:** 2024-07-25

**Authors:** Yihui Quek, Daniel Stilck França, Sumeet Khatri, Johannes Jakob Meyer, Jens Eisert

**Affiliations:** 1 Dahlem Center for Complex Quantum Systems, Freie Universität Berlin, Berlin, Germany; 2 School of Engineering and Applied Sciences, Harvard University, Cambridge, MA USA; 3 Department of Mathematical Sciences, University of Copenhagen, Copenhagen, Denmark; 4 Univ Lyon, Inria, ENS Lyon, UCBL, LIP, Lyon, France; 5 Helmholtz-Zentrum Berlin für Materialien und Energie, Berlin, Germany

**Keywords:** Quantum information, Qubits, Information theory and computation, Computational science

## Abstract

Quantum error mitigation has been proposed as a means to combat unwanted and unavoidable errors in near-term quantum computing without the heavy resource overheads required by fault-tolerant schemes. Recently, error mitigation has been successfully applied to reduce noise in near-term applications. In this work, however, we identify strong limitations to the degree to which quantum noise can be effectively ‘undone’ for larger system sizes. Our framework rigorously captures large classes of error-mitigation schemes in use today. By relating error mitigation to a statistical inference problem, we show that even at shallow circuit depths comparable to those of current experiments, a superpolynomial number of samples is needed in the worst case to estimate the expectation values of noiseless observables, the principal task of error mitigation. Notably, our construction implies that scrambling due to noise can kick in at exponentially smaller depths than previously thought. Noise also impacts other near-term applications by constraining kernel estimation in quantum machine learning, causing an earlier emergence of noise-induced barren plateaus in variational quantum algorithms and ruling out exponential quantum speed-ups in estimating expectation values in the presence of noise or preparing the ground state of a Hamiltonian.

## Abstract (web summary)

Error mitigation has helped improve the performance of current quantum computing devices. Now, a mathematical analysis of the technique suggests its benefits may not extend to larger systems.

## Main

Quantum computers promise to efficiently solve some computational tasks that are out of reach of classical supercomputers. As early as the 1980s^1^, it was suspected that quantum devices may have computational capabilities that go substantially beyond those of classical computers. Shor’s algorithm, presented in the mid-1990s, confirmed this suspicion by presenting an efficient quantum algorithm for factoring, for which no efficient classical algorithm is known^2^. Since then, quantum computing has been a hugely inspiring theoretical idea. However, it soon became clear that unwanted interactions with the environment and, hence, the concomitant decoherence are the major threats to realizing quantum computers as actual physical devices. Fortunately, early fears that decoherence could not be overcome in principle were proven wrong. The field of quantum error correction has presented ideas that show that one can still correct for arbitrary unknown errors^3^. This key insight triggered a development that led to the blueprint of what is called the fault-tolerant quantum computer^4,5^, a (so far still fictitious) device that allows for arbitrary local errors and can still maintain an arbitrarily long and complex quantum computation. That said, known schemes for fault tolerance have demanding and possibly prohibitive overheads^5^. For the quantum devices that have been realized in recent years, such prescriptions still seem out of scope.

For this reason, quantum error mitigation has gained traction recently^6–9^ as a possible near-term surrogate for quantum error correction. The idea is to correct the effect of quantum noise on a near-term computation’s result through classical post-processing of measurement outcomes, without mid-circuit measurements and adaptive gates, as is done in error correction. This minimizes the overheads in physical hardware.

Although a compelling prospect, we ask: to what extent can we really achieve such classical correction of quantum noise post hoc?

In this work, we argue that the current generation of error-mitigation schemes must be seriously reconsidered if we are to reach this goal. Empirically, some of them seem to come at a severe quantum resource penalty. At least one specific protocol, zero-noise extrapolation, requires a number of samples that scales exponentially as the number of gates in the light cone of the observable of interest, with the exponent depending on the noise levels^8^. A similar exponential scaling besets probabilistic error cancellation under a sparse noise model^10^. Our results contribute to a theoretical understanding of the conditions under which this happens.

We identify striking obstacles to error mitigation. Even to mitigate noisy circuits slightly beyond constant depth requires a superpolynomial number of uses of those circuits in the worst case. These obstacles are seen when we turn the lens of statistical learning theory onto the problem of error mitigation. We formulate this problem rigorously as one where the mitigation algorithm works with a classical description of a noiseless circuit and specimens of the noisy circuit’s output state, on which it can perform arbitrary measurements.

We then distinguish two tasks. In the first (weak error mitigation), the goal is to output a collection of expectation values of the noiseless circuit’s output state. This approach is used to mitigate errors in variational quantum circuits^6,11^, an important family of circuits for near-term quantum devices. In other situations (strong error mitigation), the goal is to output a sample from the clean output state when measured in the computational basis. This approach is used to mitigate errors in algorithms for hard combinatorial optimization problems, a class that includes the famous quantum approximate optimization algorithm^12^. Our framework and results encompass many error-mitigation protocols used in practice, including virtual distillation^13,14^, Clifford data regression^15^, zero-noise extrapolation^7^ and probabilistic error cancellation^7^.

New error-mitigation schemes are being intensely developed, even as we write^6–8^. Unsurprisingly, given the high expectations for such techniques, their limitations have also already been studied. In particular, we build on a key work^16^ that first identified limitations to quantum error mitigation by studying how noise affects the distinguishability of quantum states. In that work, as well as in refs. ^17,18^ that are concurrent to our work, the authors take an information-theoretic approach to study the sample complexity of weak error mitigation under depolarizing noise (more generally, Pauli channels and thermal noise applied to the identity circuit) and show that it scales exponentially but only with the depth of the circuit. This is not a limitation when the depth is $O(\log n)$, as is the case in practice.

In fact, the quantum community has known for some time that quantum states being manipulated by noisy quantum circuits undergoing depolarizing noise converge exponentially quickly (in depth) to maximally mixed states^19–21^. This behaviour suggests that quantum advantage is lost once circuits exceed logarithmic depth (if no error correction is used). Strikingly, in contrast to this natural expectation, our limitations kick in for circuits that come much closer to constant depth. This is because we introduce a dependence of the sample cost on the width of the circuits (*n*). We also analyse error mitigation under non-unital noise, a highly physically relevant class of noise that includes *T*_1_ decay, the primary source of noise in superconducting qubit architectures. In addition to the mathematical tools we developed for analysing noisy computation, we make a strong conceptual point: error-mitigation schemes that have empirically been found in practice to have $\exp (n)$ sample complexity are pretty much as good as they can get, unless they are crafted to address circuits with a special structure that eludes our bounds.

Although quantum error mitigation has seen practical success on small noisy quantum devices^9^, our results put hard limits on how much we can expect error mitigation to scale with the size of such devices. We have demonstrated worst-case circuits that must be run exponentially many times for error mitigation on them to work. Lower bounds shine light on upper bounds. Our work suggests that future sample-efficient error-mitigation schemes must dodge the limitations we have identified. How then can we design circuits to be more resilient to noise, and how far into the feasible regime can we push the line between error mitigation and error correction?

## Introduction to the technique

**Fig. 1:**
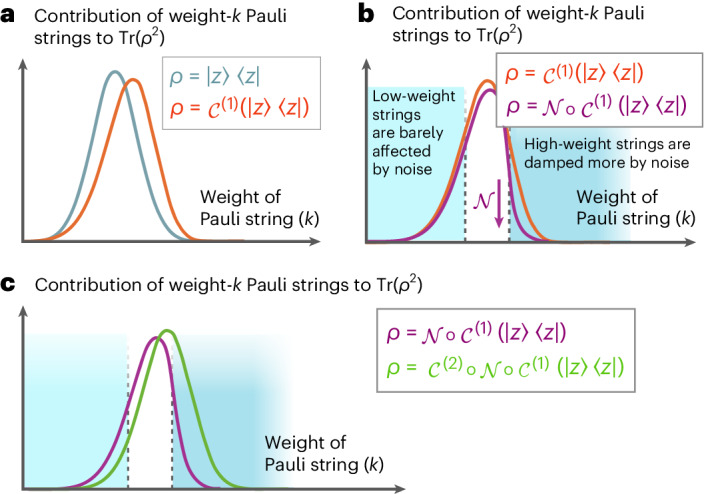
Intuition for our circuit construction: the higher the weight of a Pauli string, the more sensitive it is to Pauli noise (see equation (26) in the Supplementary Information). **a**–**c**, Whereas, for product pure states, there are Pauli strings with constant-order expectation values for all weights, most quantum states have correlators of only high weight. Thus, most random states are substantially more sensitive to noise than product quantum states. **a**, The effect of applying a noiseless Pauli mixing circuit to a computational basis state is to shift the binomial of contributions to a weighted binomial (compare equation (31) to equation (35)). **b**, The effect of applying one subsequent layer of depolarizing noise on the output of the aforementioned circuit (compare equation (35) to equation (38)). **c**, The effect of applying yet another Pauli mixing layer to the state output by the aforementioned circuit (as captured in equation (35)).

**Fig. 2:**
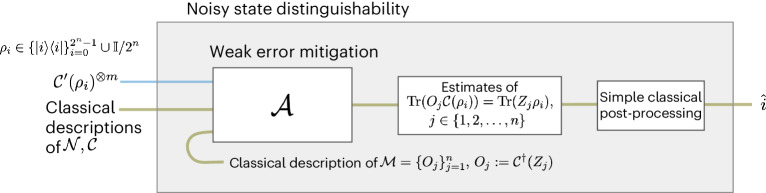
Quantum error mitigation as a statistical inference problem. To lower-bound the sample complexity of weak error mitigation, we show that it can be used as a subroutine to solve a constructed problem of distinguishing states under noise.

**Fig. 3:**
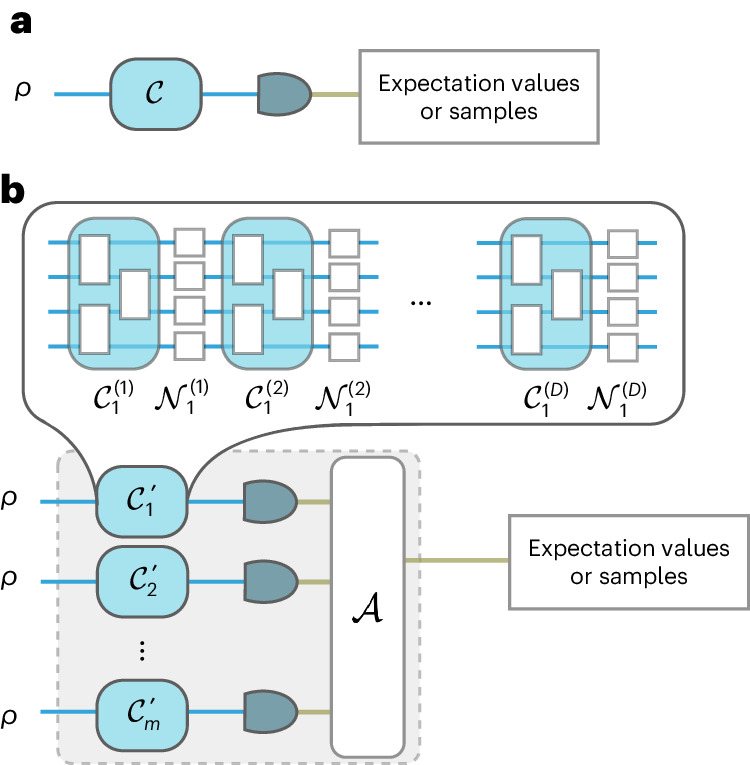
Circuits without and with quantum noise. **a**, Idealization of near-term quantum algorithms without quantum noise. Most such algorithms work by running an *n*-qubit quantum circuit ${{{\mathcal{C}}}}$ on an input quantum state *ρ*, measuring the output state and then returning either samples from the resulting probability distribution or expectation values of specified observables. **b**, The model of error mitigation used in this work, building on the framework established in ref. ^16^. The quantum channel ${{{{\mathcal{C}}}}}_{i}^{{\prime} }$ represents the *i*th run of ${{{{\mathcal{C}}}}}^{{\prime} }$, the noisy version of ${{{\mathcal{C}}}}$. We model the noise acting on ${{{\mathcal{C}}}}$ by interleaving its layers with layers of a given noise channel. In Supplementary Information Section I, we show that this model applies to practical error-mitigation protocols such as virtual distillation^13,14^, Clifford data regression^15^, zero-noise extrapolation^7^ and probabilistic error cancellation^7,8^. In this work, we study how *m*, the number of noisy circuit runs, scales with *n* and *D* to reliably recover the expectation values.

To establish the framework, we start off by defining what constitutes error mitigation in the rest of this work. In the literature, the term ‘error mitigation’ has been used to describe protocols that are appended after a noisy quantum algorithm. Such protocols reduce the unwanted effect of noise on a quantum circuit by measuring it and classically post-processing the results (Figs. 1–3). They then output either samples from the would-be noiseless circuit or its expectation values on observables of interest, depending on the purpose of the original quantum algorithm. More concretely, we formulate the problem of error mitigation as follows.

### Problem 1

(Error mitigation (informal)) *Upon input of:*

A. *(1) a classical description of a noiseless circuit* ${{{\mathcal{C}}}}$ *and a finite set* ${{{\mathcal{M}}}}=\{{O}_{i}\}$ *of observables, and*
B. *(2) copies of the output state* ${\sigma }^{{\prime} }$ *of the noisy circuit* ${{{{\mathcal{C}}}}}^{{\prime} }$, *and the ability to perform collective measurements, then output either:*

A. *(1) estimates of the expectation values* ${{\mathrm{Tr}}}\,({O}_{i}\sigma )$ *for each* ${O}_{i}\in {{{\mathcal{M}}}}$ *(weak error mitigation), where σ is the output state of the noiseless quantum circuit* ${{{\mathcal{C}}}}$, *or*
B. *(2) samples from a probability distribution close to the distribution of σ when measured in the computational basis (strong error mitigation).*

*The number* *m* *of copies of* ${\sigma }^{{\prime} }$ *needed is the sample complexity of* (*weak or strong*) *error mitigation*.

The bounds we prove apply even to error-mitigation protocols that are given an exact description of the noise model affecting the circuit. Naturally, this also means our bounds apply to those protocols that know nothing about the noise and must learn it on the fly, as they are a subset of the protocols mentioned in the previous sentence. We refer to the Supplementary Information for rigorous definitions of error-mitigation algorithms (see Definitions 5 and 6 therein) and for an explanation of how these definitions connect to well-known protocols such as zero-noise extrapolation and probabilistic error cancellation (see Section I therein). These protocols, for instance, run ${{{\mathcal{C}}}}$ with varying levels of noise and then apply simple post-processing to ${{{\mathcal{C}}}}$ or modify the circuit based on the noise. This level of abstraction of error-mitigation protocols builds on those of refs. ^16–18^.

In this work, we reduce a statistical inference problem on noisy quantum states to error mitigation on the circuits that produced them. What motivates this perspective is the observation that a ‘good’ error-mitigation algorithm should act as an effective denoiser, allowing one to distinguish one state from another, even if the states can be accessed only by measuring their noisy versions. Seen from this angle, error mitigation solves the following problem.

### Problem 2

(Noisy state discrimination (informal)) *Upon input of:*

*(1) classical descriptions of a set S* = {*ρ*_0_, *ρ*_1_, …, *ρ*_*N*_} *of n*-*qubit quantum states, a noiseless circuit*${{{\mathcal{C}}}}$, *and*
*(2) m* *copies of a state* ${{{{\mathcal{C}}}}}^{{\prime} }(\;{\rho }_{i})$ *(where* ${{{{\mathcal{C}}}}}^{{\prime} }$ *is the noisy version of* ${{{\mathcal{C}}}}$*), with i* ∈ {0, …, *N*} *unknown, and the ability to perform collective measurements, then output* $\hat{i}\in \{0,\ldots ,N\;\}$ *such that* $\hat{i}=i$ *(‘success’) with high probability.*

Problems 1 and 2 are intimately related. To see this, consider in Problem 2 discriminating between the maximally mixed state ${\rho }_{N}:={\mathbb{I}}/{2}^{n}$ and the states ${\rho }_{x}:=\left\vert x\right\rangle \left\langle x\right\vert$ for *x* ∈ {0, 1}^*n*^. This problem can be solved by performing weak error mitigation with the observables ${{{\mathcal{M}}}}={\{{{{\mathcal{C}}}}({Z}_{i})\}}_{i\in [n]}$. This is because a weak error-mitigation algorithm should output the estimates $\operatorname{Tr}({{{\mathcal{C}}}}({Z}_{i}){{{\mathcal{C}}}}({\rho }_{x}))=\operatorname{Tr}({Z}_{i}{\rho }_{x})$. Now consider two cases: (1) if the unknown state had been ${{{{\mathcal{C}}}}}^{{\prime} }({\rho }_{x})$ for *x* ∈ {0, 1}^*n*^, then $\operatorname{Tr}(\;{\rho }_{x}{Z}_{i})=2{x}_{i}-1$; (2) if instead the unknown state had been ${{{{\mathcal{C}}}}}^{{\prime} }(\;{\rho }_{N})$, then $\operatorname{Tr}(\;{\rho }_{N}{Z}_{i})=0,\forall i\in [n]$. The output of Problem 1 thus completely identifies the label of the state *i*, thus solving Problem 2. The upshot is as follows. If at least *m* copies of the noisy state must be used for successful discrimination in the above setting (solving Problem 2), then at least the same number of copies are needed for successful weak error mitigation (solving Problem 1). We use the information-theoretic generalized Fano method for multiple hypothesis testing^22^ to provide a lower bound on *m*. The crucial quantity to study, it turns out, is the quantum relative entropy:

1 $$D({{{{\mathcal{C}}}}}^{{\prime} }(\;{\rho }_{x})\parallel {\mathbb{I}}/{2}^{n}).$$

The inverse rate of decay of this quantity bounds the sample complexity of error mitigation. We use tools to precisely control the quantum relative entropies of unitary two-designs under unital and non-unital quantum noise so that we can construct circuits ${{{{\mathcal{C}}}}}^{{\prime} }$ that yield our strong bounds on error mitigation.

## Results

With our information-theoretic perspective, we are able to establish the following fundamental limits on a broad swath of error-mitigation protocols.

### Sample complexity of error mitigation for local depolarizing noise

Intuitively, one would expect the sample complexity of error mitigation to scale with the size of the noiseless circuit ${{{\mathcal{C}}}}$ and the amount of noise affecting it. Our Theorem 1 confirms this intuition starkly. It shows that the dependence of the resource requirement on these parameters is exponentially higher than previously known. Concretely, Theorem 1 hinges on showing that the distance of a noisy circuit’s output state to the maximally mixed states goes below constant order at $\operatorname{poly}\log\log n$ depth, whereas the onset of this effect at the exponentially larger $\log n$ depth pointed out in previous works was already detrimental to many near-term applications^16–18,21,23,23–27^.

#### Theorem 1

(Number of samples for mitigating depolarizing noise scales exponentially with the number of qubits and depth) *Let* ${{{\mathcal{A}}}}$ *be a weak error-mitigation algorithm that mitigates the errors in an n*-*qubit, D-layer quantum circuit* ${{{\mathcal{C}}}}$ *affected by local depolarizing noise* ${{{\mathcal{N}}}}$ *of parameter p. For some parameter s* > 0 *and depths* $D\ge {{\varOmega }}({\log}^{2}(n/s))$, ${{{\mathcal{A}}}}$ *requires as input at least s*^−1^*p*^−*Ω*(*n* *D*/*s*)^ *copies of the output state* ${\sigma }^{{\prime} }$ *of the noisy quantum circuit* ${{{{\mathcal{C}}}}}^{{\prime} }$.

Refer to Supplementary Information Theorem 4 for the full, formal statement. In other words, setting $s={{{\mathcal{O}}}}(1)$ tells us that error mitigation for even a circuit of polylogarithmic depth demands exponentially many (in qubit number) samples, in the worst case. By picking, for example, the parameter $s={{\varOmega }}(n/{\log }^{2}(n))$, we see that even at the low depth $D=\operatorname{poly}\log\log(n)$, error mitigation already requires superpolynomially many samples. Figure 1 illustrates the intuition behind our construction of circuits that saturate this bound. They rapidly entangle and shift weight onto high-Hamming weight Paulis, which makes them particularly sensitive to noise.

Note, however, that these circuits, which leverage a construction by ref. ^28^, require all-to-all connectivity to implement, so that they mix rapidly (that is, at short depths). In Supplementary Information Section II.E.1, we show analogous results for geometrically local circuits on a *d*-dimensional lattice. In that setting, we show that exponentially many samples are required for error mitigation at depths of ${{{\mathcal{O}}}}({n}^{1/d}\operatorname{poly}\log(n))$. Even at depths $\tilde{{{{\mathcal{O}}}}}({\log}^{2/d}(n)\operatorname{poly}\log\log(n))$, error mitigation requires superpolynomially many samples. These effects kick in at depths $\tilde{{{{\mathcal{O}}}}}({n}^{1/d})$, which is the minimal depth required to ensure that all qubits have a light cone proportional to the system size. These constructions thus illustrate a refined version of the basic intuition: rather than the size of the circuit, it is more precisely the number of gates in the light cone of observables that determines the difficulty of error mitigation.

### Sample complexity of error mitigation for non-unital noise

Our result in Theorem 1 relies critically on the structure of depolarizing noise, namely, that it is a Pauli channel, and thus, it is unital. Moving beyond this toy model, we are also able to show sample complexity bounds for circuits affected by non-unital noise, which is notably trickier to analyse. Here, the essence of our conclusion remains the same. Whenever a family of circuits is highly entangling, which we model through the assumption that it forms a unitary two-design, mitigating local non-unital noise typically requires a number of samples that is exponential with both the number of channel applications and number of qubits.

#### Theorem 2

(Number of samples for mitigating non-unital noise scales exponentially as the number of qubits and depth) *Weak error mitigation on an n-qubit, D-layer noisy quantum circuit of the form* $\bigcirc_{t=1}^D {\mathcal{N}}_t\circ {\mathcal{U}}_t$*, where the* ${{{{\mathcal{U}}}}}_{t}$ *are drawn independently from a unitary two-design and where* ${{{{\mathcal{N}}}}}_{t}={{{{\mathcal{N}}}}}^{\otimes n}$ *is an n-fold tensor product of a qubit non-unital noise channel* ${{{\mathcal{N}}}}$*, requires as input at least c*^−*Ω*(*n* *D*)^ *of the circuit’s output state, for some constant c that depends on the noise* ${{{\mathcal{N}}}}$.

Refer to Supplementary Information Theorem 7 for the full, formal statement. To prove it, we compute the expected overlap of two quantum states that are output by *D* alternating layers of a unitary sampled from a unitary two-design followed by a non-unital noise channel. Although this model of only applying local noise after a (global) unitary is simplified, we study error mitigation under non-unital noise beyond the setting of the variational algorithms studied in ref. ^23^. Our observation here that global unitaries result in fast decay to the maximally mixed state under non-unital noise further strengthens the evidence for a connection between the entanglement generated by a circuit and the rate at which noise spreads.

We prove Theorems 1 and 2 in the setting of weak error mitigation, and we assume that the error-mitigation algorithm does not use knowledge of the input state to the circuit. Although this is the setting most often used in practice, it does not cover all proposals in the literature. We now ask whether our results can be extended to close variants of this setting that are also practically relevant.

### Dependence on the initial state

The strong limitations we have proven so far are based on the assumption that the error-mitigation algorithm does not make any use of information about the input state to the noisy circuit so that the error-mitigation algorithm is input-state agnostic. Does lifting this restriction—thereby including input-state-aware error-mitigation protocols—change the picture substantially? A first observation is that we can no longer rule out the possibility of successful error mitigation with subexponential worst-case sample complexity simply because a classical simulation with no resort to the noisy quantum device at all is a valid error-mitigation algorithm with zero sample complexity, albeit possibly being computationally hard. Our no-go result in this case thus takes a different perspective: we show that an error-mitigation algorithm that makes use of a noisy quantum device must use exponentially many copies of its noisy output state to produce an output meaningfully different from some fully classical procedure that does not invoke the quantum device.

#### Theorem 3

(Resource cost of successful error mitigation) *A successful weak error-mitigation algorithm* ${{{\mathcal{A}}}}$ *must use* $m={c}^{{{{\mathcal{O}}}}(nD)}$ *copies of the noisy output state* ${\sigma }^{{\prime} }$ *in the worst case, or there exists an equivalent algorithm* ${{{{\mathcal{A}}}}}^{{\prime} }$ *with purely classical inputs such that the output of* ${{{{\mathcal{A}}}}}^{{\prime} }$ *is indistinguishable from the output of* ${{{\mathcal{A}}}}$.

For the full, formal statement, see Supplementary Information Theorem 2. In this way, we show that our constructions apply both to the input-state-agnostic and input-state-aware settings.

### Interrelation between strong and weak error mitigation

Our work defines and studies strong error mitigation. The relevance of this notion is that, in some cases, weak error mitigation does not quite achieve the algorithmic goal at hand, for instance, solving hard combinatorial optimization problems on a noisy quantum device. In that case, one is not only necessarily interested in the optimal value of the cost function (obtained through weak error mitigation) but also in an assignment that achieves that value (obtained through strong error mitigation). Although it is not hard to see that for local observables, strong error mitigation implies weak error mitigation, we also ask the converse question. Could there be an algorithm that takes in the output of weak error mitigation (expectation values) and ‘bootstraps’ them into the output of strong error mitigation (samples)? We give a partial negative answer that rules out certain types of protocols.

#### Theorem 4

(Exponentially many observables (in the same eigenbasis) are needed to output samples from the same basis) *Suppose we have a weak error-mitigation algorithm that, given a set* ${{{\mathcal{M}}}}={\{{O}_{i}\}}_{i}$ *of observables with the same eigenbasis, outputs their expectation value estimates* $\{{\hat{o}}_{i}\}$*. In general, at least* $\exp (n)$ *many distinct* ${\hat{o}}_{i}$ *must be queried by any algorithm that takes as input the* $\{{\hat{o}}_{i}\}$ *and outputs samples from their eigenbasis.*

We refer readers to Supplementary Information Theorem 9 for the full, formal statement. The proof of this statement takes the perspective of expectation value estimates as statistical queries^29^ and leverages existing results on optimality guarantees of hypothesis selection^30^ and on the statistical query hardness of learning parity functions^31^.

### Consequences of our results

#### The interplay of entanglement and noisy computation

At the heart of our technical results is entanglement. The circuits we construct are built of highly entangling gates that allow them to scramble quickly to the maximally mixed state, confirming the intuition that such circuits are exponentially more sensitive to noise than general circuits. This opens up an intriguing research direction. How does the amount of entanglement generated by a quantum circuit relate to its noise sensitivity?

That studying entanglement could be key to defeating noise is illustrated by the contrast between our results and those of ref. ^27^. The latter showed that on expectation, the total variation distance between the output distribution ${{{\mathcal{D}}}}$ of a noisy random quantum circuit and a uniform distribution decays exponentially with depth only:

2 $$\begin{array}{l}{{\mathbb{E}}}_{{{{\mathcal{B}}}}}\left[\parallel {{{\mathcal{D}}}}-U{\parallel }_\mathrm{TV}\right]={{\varOmega }}(\exp (-CD)).\end{array}$$

This result applies to the noisy circuit ensemble ${{{\mathcal{B}}}}$ created by applying uniformly random two-qubit Clifford gates at each step, whereas our circuit ensemble is more structured. Viewing the quantity on the left-hand side of equation (2) as analogous to our equation (1), however, we see that our circuit ensemble also mixes faster than theirs (as the relative entropy of our ensemble decays exponentially fast with both *n* and *D*). We believe that this difference arises precisely because ${{{\mathcal{B}}}}$ is not as entangling as our ensemble. (We substantiate this with the following oversimplified summary of their proof: a constant fraction of Clifford gates acting on two qubits are a product of one-qubit gates. Thus, at each step there is a constant probability *q* > 0 that the gate we pick to act on qubit 1 is a product. As the gates at different layers are independent, at depth *D* with probability at least *q*^*D*^, the first qubit of the system will be in a product state with the rest. In that case, the effect of the noise on that qubit cannot depend on the system size *n*.)

#### Loss of quantum advantage at log log *n* depth

Proofs of Theorem 1, which studies the convergence of a noisy circuit’s output state to the maximally mixed state, have appeared in various forms before. Fast convergence is disastrous for error mitigation^16–18,23,24^, kernel estimation^25^ in quantum machine learning as well as the depth at which quantum advantage from sampling noisy random quantum circuits^27^ or variational quantum algorithms^21,23,26^ can be obtained. However, these results share the common feature that they are able to show only an exponential-with-depth contraction of the circuit’s output state towards the maximally mixed state and show only that the distance to the maximally mixed state goes below constant order at logarithmic depth. Furthermore, one can show that such results are tight for trivial circuits consisting solely of the identity gate.

We point out that the aforementioned applications pertain to noisy intermediate-scale quantum processors. Such processors run shallow quantum circuits, which means that their depth scales like $D=O(\log (n))$, so that the rate of convergence in the aforementioned results is inversely polynomial with *n*. In this depth regime, our circuits converge exponentially fast with *n*. The onset of the attendant effects is, therefore, exponentially earlier in our circuits. At $\log\log(n)$ depth, increasing the number of qubits already brings about a superpolynomial drop in distinguishability from the maximally mixed state.

In addition, our results imply that there will be no exponential quantum speed-ups for estimating expectation values in the presence of noise. This is because there are classical algorithms that exhibit the same exponential scaling with complexity^32,33^ exhibited by our results. As such, both classical and quantum algorithms will have exponential complexity for this task; quantum can at best improve the exponent.

#### No noisy circuits for ground state preparation

Our work also bears on a task originating from the quantum sphere: preparing the ground state of a Hamiltonian. When optimizing the energy of a Hamiltonian, the outputs of noisy quantum circuits concentrate strongly^21,23^, which causes any possibility of quantum advantage to be lost at a depth *D* for any circuit for which the error probability satisfies *p* = *Ω*(*D*^−1^), an argument that follows a similar route as our work. Our contributions show that this generic bound can be loose in the worst case, as we show that the 2-Rényi entropy can decrease exponentially faster. Thus, in the worst case and at sufficiently high depth *D* polylogarithmic with *n*, unless the error probability is $p={{{\mathcal{O}}}}({(nD)}^{-1})$, the outputs of noisy circuits will concentrate around strings with energies that do not offer a quantum advantage. Unfortunately, it has been shown that for many important classes of local Hamiltonians^23,34–37^, any circuit that aims at preparing the ground state has to have at least logarithmic depth. For highly entangled ground states of non-local Hamiltonians, we expect even larger lower bounds on the depth at which geometrically local circuits can prepare such ground states, as entanglement has to be built between distant sites. The fast build-up of entanglement, however, is also a key reason that our circuits display a system-size-dependent decay to the maximally mixed state. Together these statements imply that noisy variational ground state preparation is mostly a lose–lose proposition. Either the depth is insufficient to reach a good approximation of the ground state or it is so high that noise takes over.

### Outlook

We have established a general rigorous framework that encapsulates large classes of schemes for quantum error mitigation that are already being used on today’s noisy quantum processors. For these schemes, we have identified severe information-theoretic limitations that are exponentially tighter than what was previously known^16–18^, due to the additional dependence we have now identified of the sampling cost on the width of the circuit.

Although our bounds are still worst-case bounds, they are worst case within a class of quantum circuits that can be as shallow as $\operatorname{poly}\log \log (n)$, which is substantially closer to practice than the *d* = *Ω*(*n*) of previous bounds. To close this gap even further, one should aim to lift the requirement of global connectivity in our circuits. This also hints at a prescription for practitioners: choosing suitably local quantum circuits may improve the performance of error-mitigation schemes (although potentially coming at the price of reduced expressivity). It would be intriguing to examine whether this is why some error-mitigation protocols run in the recent past have been found to fare rather well in practice.

We reiterate that our results do not rule out quantum error mitigation for tackling suitably low noise levels in existing quantum architectures, but they should be viewed as a clarion call for deepened understanding. What aspects of our worst-case construction of circuits carries over into a typical use case? We believe that the spread of entanglement has to play a role. Broadly speaking, we see two regimes emerge once we go to the Heisenberg picture. For the first, the evolution does not spread out the observables or generate much entanglement. However, in this regime, we expect certain simulation techniques like tensor networks to perform well too. Second, in the other extreme, the evolution does generate considerable entanglement, and this is the regime our circuits operate in. Then, error mitigation will probably require exponential resources, as we have found. What we have observed, then, is a tension at the heart of near-term quantum computing. Although it is known that entanglement needs to spread extensively over the architecture so that one can hope for a quantum advantage, we see here (as well as in ref. ^38^) that this spreading of entanglement can be an adversary, as it also assists noise in spreading rapidly.

We strongly believe that the way forward for dealing with errors in quantum computation will involve new schemes that are intermediate between mere quantum error mitigation and resource-expensive fault-tolerant quantum computing. In the medium term, some ‘parsimonious’ form of quantum error correction involving limited amounts of quantum redundancy will presumably be necessary. How much is enough? Clarifying this point will be an important step in bringing quantum computers closer to reality.

## Methods

In this section, we present substantial details of the arguments presented in the main text. In ‘Preliminaries: relative entropy and derived quantities’, we provide some background and define relative entropies, elements of the Pauli group and unitary two-designs that we need for our proofs. Then, in ‘Error mitigation: the lay of the land’, we introduce our error-mitigation setting as well as two variants of error mitigation that we call weak and strong, respectively. In Supplementary Information Section I, we further argue that this setting encompasses many error-mitigation protocols used in practice, including virtual distillation^13,14^, Clifford data regression^15^, zero-noise extrapolation^7^ and probabilistic error cancellation^7,8^. Although most theoretical analyses of error mitigation have focused exclusively on the weak error-mitigation setting, here we argue with reference to the practical protocols that the strong setting is equally relevant, and we make a first attempt at relating the two settings. We also show that many existing error-mitigation protocols fit into the model that we will work with.

## Preliminaries: relative entropy and derived quantities

At the heart of many of our technical arguments is relative entropy, which is a measure of the distance between two quantum states, and how relative entropy contracts under noise. We first define distance measures on classical probability distributions. Let *R* and *S* be two probability measures on the same support ${{{\mathcal{X}}}}$. It will suffice for our purposes to let ${{{\mathcal{X}}}}$ be a finite set.

- **Total variation distance**. This is defined as
  3 $${d}_\mathrm{TV}(R,S):=\sup_{A\subseteq {{{\mathcal{X}}}}}| R(A)-S(A)| =\frac{1}{2}\sum\limits_{x\in {{{\mathcal{X}}}}}| R(x)-S(x)| .$$
- **Kullback–Leibler divergence or classical relative entropy**. The classical relative entropy is defined as
  4 $$D(R| | S):=\sum\limits_{x\in {{{\mathcal{X}}}}}R(x)\log \frac{R(x)}{S(x)}={{\mathbb{E}}}_{x \sim R}\left[\log \frac{R(x)}{S(x)}\right],$$
  where throughout the manuscript we take $\log$ to be base 2.

Now we introduce distance measures on quantum states. The primary such measure we will consider is quantum relative entropy, which can be understood as a quantum generalization of classical Kullback–Leibler divergence. For this reason, we will use the same notation *D*( ⋅ ∥ ⋅ ) for both quantum relative entropy and classical relative entropy.

Let *ρ* and *σ* be two quantum states in $D({{{{\mathcal{H}}}}}_{n})$ (though, in general, these quantities are defined with *σ* any positive semi-definite operator). We will use the following divergences:

- **Relative entropy**. If supp(*ρ*) ⊆ supp(*σ*), we define the (quantum) relative entropy as
  5 $$D(\;\rho \parallel \sigma ):=\operatorname{Tr}(\;\rho \log \rho )-\operatorname{Tr}(\;\rho \log \sigma ).$$
- **Petz–Rényi relative entropy**. For a parameter *α* ∈ (0, 1) ∪ (1, *∞*),
  6 $${D}_{\alpha }(\;\rho \parallel \sigma ):=\frac{1}{\alpha -1}\log\operatorname{Tr}\left[{\rho }^{\alpha }{\sigma }^{1-\alpha }\right].$$
  For *α* ∈ (1, *∞*), this definition holds when supp(*ρ*) ⊆ supp(*σ*). In the limit *α* → 1, Petz–Rényi relative entropy reduces to the quantum relative entropy, that is, $\lim_{\alpha \to 1}{D}_{\alpha }(\;\rho \parallel \sigma )=D(\;\rho \parallel \sigma )$.
- **Maximum relative entropy**. For supp(*ρ*) ⊆ supp(*σ*),
  7 $${D}_{\max }(\;\rho \parallel \sigma ):=\inf \left\{\gamma :\rho \le {2}^{\gamma }\sigma \right\}.$$
  It holds that $D(\;\rho \parallel \sigma )\le {D}_{\max }(\;\rho \parallel \sigma )$ (ref. ^39^).

It will often be illuminating to fix the second argument of relative entropy to be the maximally mixed state, while putting the state of interest in the first argument of relative entropy. Relative entropies of this form may even be upper-bounded explicitly in terms of quantities relating to the state of interest, as we now show the following.

### Lemma 1

(Purity controls relative entropy to the maximally mixed state) *For any ρ* *on n qubits,*

8 $$D\left(\rho\Big\Vert \frac{{\mathbb{I}}}{{2}^{n}}\right)\le n+\log (\operatorname{Tr}(\;{\rho }^{2})).$$

### Proof

The statement follows from the fact that relative entropy is upper-bounded by the 2-Rényi relative entropy:

9 $${D}_{2}(\;\rho \parallel \sigma ):=\log\operatorname{Tr}\left[{\rho }^{2}{\sigma }^{-1}\right].$$

This can be seen because Petz–Rényi relative entropy for *α* ∈ (0, 1) ∪ (1, *∞*) satisfies an ordering property^39^, that is,

10 $${{{\rm{for}}}}\,\alpha > \beta > 0,\;{D}_{\alpha }(\;\rho \parallel \sigma )\ge {D}_{\beta }(\;\rho \parallel \sigma ),$$

and

11 $$\lim_{\alpha \to 1}{D}_{\alpha }(\;\rho \parallel \sigma )=D(\;\rho \parallel \sigma ).$$

For any *ρ* on *n* qubits and *σ* the maximally mixed state, we thus have

12 $$\begin{aligned}D\left(\rho\Big\Vert \frac{{\mathbb{I}}}{{2}^{n}}\right)&\le {D}_{2}\left(\rho\Big\Vert \frac{{\mathbb{I}}}{{2}^{n}}\right)\\&=\log\operatorname{Tr}\left({\rho }^{2}{\left(\frac{{\mathbb{I}}}{{2}^{n}}\right)}^{-1}\right)\\&= n+\log(\operatorname{Tr}(\;{\rho }^{2})),\end{aligned}$$

as stated.

### Pauli operators and Pauli channels

We denote the single-qubit Pauli operators by ${\mathbb{I}},X,Y\;\text{and}\;Z$. Furthermore, for *a* = (*a*_1_, *a*_2_, …, *a*_*n*_) ∈ {0, 1}^*n*^, we let

13 $${Z}^{\;a}:={Z}^{\;{a}_{1}}\otimes {Z}^{\;{a}_{2}}\otimes \cdots \otimes {Z}^{\;{a}_{n}},$$

14 $${X}^{\;a}:={X}^{\;{a}_{1}}\otimes {X}^{\;{a}_{2}}\otimes \cdots \otimes {X}^{\;{a}_{n}},$$

where

15 $${Z}^{\;{a}_{i}}:=\left\{\begin{array}{l}{\mathbb{I}},\quad{a}_{i}=0\\ Z,\quad{a}_{i}=1\end{array}\right.,$$

16 $${X}^{\;{a}_{i}}:=\left\{\begin{array}{l}{\mathbb{I}},\quad{a}_{i}=0\\ X,\quad{a}_{i}=1\end{array}\right.\,.$$

We then define the Pauli group.

#### Definition 1

(Pauli group and Pauli weight) *Let n* ∈ {1, 2, …}*. The Pauli group* ${{{{\mathcal{P}}}}}_{n}$*, by definition, consists of all operators of the form i*^*k*^*X*^*a*^*Z*^*b*^*, where k* ∈ {0, 1, 2, 3} *and a*, *b* ∈ {0, 1}^*n*^*. Let* ${{{{\mathcal{Q}}}}}_{n}:={{{{\mathcal{P}}}}}_{n}/\{\pm 1,\pm i\}$ *be the quotient group that results from disregarding global phases in* ${{{{\mathcal{P}}}}}_{n}$*. For every Pauli operator* $P\in {{{{\mathcal{Q}}}}}_{n}$*, we denote by w*(*P*) *the weight of P, which is the number of qubits on which P acts non-trivially*.

We remind readers of the following basic fact about Pauli operators. For *n*-qubit Paulis $P,Q\in {{{{\mathcal{Q}}}}}_{n}$,

17 $$\operatorname{Tr}(P\cdot Q)=\begin{cases}{2}^{n}&P=Q,\\ 0&{{\mbox{otherwise.}}}\end{cases}$$

A great deal of our analysis is devoted to error mitigation on circuits affected by depolarizing noise. Depolarizing noise is an example of a Pauli channel, which are channels that act on Hilbert space operators as

18 $${{{\mathcal{P}}}}(\cdot )=\sum\limits_{P\in {{{{\mathcal{Q}}}}}_{n}}{q}_{P}P(\cdot )P,$$

where *q*_*P*_ is a probability distribution over ${{{{\mathcal{Q}}}}}_{n}$.

#### Depolarizing noise

##### Definition 2

(Depolarizing channels) *For* $M\in L({{\mathbb{C}}}^{d})$*, the d-dimensional depolarizing channel* ${{{{\mathcal{D}}}}}_{p}^{(d)}$*, for d* ≥ 2*, is defined as*

19 $${{{{\mathcal{D}}}}}_{p}^{(d)}(M\;):=pM+(1-p)\operatorname{Tr}[M\;]\frac{{\mathbb{I}}}{d},\quad p\in [-1/({d}^{\;2}-1),1].$$

When the superscript for the dimension is omitted, we implicitly refer to the single-qubit depolarizing channel (with *d* = 2):

20 $${{{{\mathcal{D}}}}}_{p}(M\;):= {{{{\mathcal{D}}}}}_{p}^{(2)}(M\;)=pM+(1-p)\operatorname{Tr}[M\;]\frac{{\mathbb{I}}}{2},\quad{}p\in [-1/3,1].$$

Note that the single-qubit depolarizing channel has an alternative representation in terms of the Pauli operators:

21 $${{{{\mathcal{D}}}}}_{p}(\;\rho )={q}_{X}X\rho X+{q}_{Y}Y\rho Y+{q}_{Z}Z\rho Z+{q}_{{\mathbb{I}}}\rho ,$$

where

22 $${q}_{X}={q}_{Y}={q}_{Z}=\frac{1-p}{4}$$

and

23 $${q}_{I}=\frac{1+3p}{4}.$$

In the context of depolarizing noise acting within an *n*-qubit circuit, a global depolarizing channel is simply a 2^*n*^-dimensional depolarizing channel. Using (17), we find that the global depolarizing channel on *n* qubits can be written as

24 $${{{{\mathcal{D}}}}}_{p}^{({2}^{n})}(P)=pP+(1-p){\delta }_{P,{\mathbb{I}}}{\mathbb{I}},\;\forall \,P\in {{{{\mathcal{P}}}}}_{n}.$$

Alternatively, one could also model the noise within a circuit as an *n*-fold local (single-qubit) depolarizing channel ${{{{\mathcal{D}}}}}_{p}^{\otimes n}$. In particular, the single-qubit depolarizing channel has the property that

25 $${{{{\mathcal{D}}}}}_{p}(X\;)=pX,\quad{{{{\mathcal{D}}}}}_{p}(Y\;)=pY,\quad{{{{\mathcal{D}}}}}_{p}(Z\;)=pZ\quad{{\mbox{and}}}\quad{{{{\mathcal{D}}}}}_{p}({\mathbb{I}})={\mathbb{I}}.$$

The following lemma shows that depolarizing noise is particularly amenable to analysis in the Pauli basis.

##### Lemma 2

(Action of depolarizing noise on a Pauli string depends on its weight) *For all p* ∈ [−1/3, 1] *and* $P\in {{{{\mathcal{P}}}}}_{n}$,

26 $${{{{\mathcal{D}}}}}_{p}^{\otimes n}(P)={p}^{w(P)}P.$$

##### Proof

This follows immediately from (21) and the definition of ${{{{\mathcal{D}}}}}_{p}^{\otimes n}$. □

### Unitary two-designs and Clifford unitaries

A unitary *t*-design, for *t* ∈ {1, 2, …}, is a finite ensemble ${\{(1/K,{U}_{k})\}}_{k = 1}^{K}$ of unitaries such that^40^

27 $$\frac{1}{K}\sum\limits_{k=1}^{K}{U}^{\otimes t}\otimes {({U}^{{\dagger} })}^{\otimes t}={\int}_{\!\!U}{U}^{\otimes t}\otimes {({U}^{{\dagger} })}^{\otimes t}\,{{\mbox{d}}}U,$$

where the integral on the right-hand side is with respect to the Haar measure on the unitary group. The *n*-qubit Clifford group ${{{{\mathcal{C}}}}}_{n}$ forms a unitary two-design (Theorem 1 in ref. ^40^), where by definition the Clifford group is the normalizer of the Pauli group ${{{{\mathcal{P}}}}}_{n}$ (ref. ^41^), that is the unitaries that map elements of the Pauli group to elements of the Pauli group under conjugation.

When the unitaries in a given ensemble are all Cliffords, the ensemble additionally possesses the following property^28^, which will be crucial to us.

#### Definition 3

(Pauli mixing) *Consider an ensemble* ${{{\mathcal{E}}}}={\left\{\;{p}_{i},{U}_{i}\right\}}_{i = 1}^{k}$ where ${U}_{i}\in {{{{\mathcal{C}}}}}_{n}$. ${{{\mathcal{E}}}}$ *is Pauli mixing, if for all* $P\in {{{{\mathcal{Q}}}}}_{n}$ *such that P* ≠ *I, the distribution* $\left\{{p}_{i},{\pi }_{{U}_{i}}(P)\right\}$ *is uniform over* ${{{{\mathcal{Q}}}}}_{n}\backslash \{I\;\}$*, where* ${\pi }_{{U}_{i}}$ *is the permutation of* ${{{{\mathcal{Q}}}}}_{n}\backslash \{I\;\}$ *induced by conjugating P by U*_*i*_.

The ensemble is a Clifford two-design (a unitary two-design whose elements are Cliffords) if and only if it is Pauli mixing^42^.

### Decay of purities

Consider that the *n*-fold Paulis are an orthogonal basis for ${{{{\mathcal{H}}}}}_{2}^{\otimes n}$, and recall that the purity upper-bounds the relative entropy to the maximally mixed state (Lemma 1). A conceptual cornerstone of our proof construction is to consider how the purity of states decays after each successive layer in the circuit by looking at the distribution over contributions of weight-*k* Pauli strings to this quantity. More rigorously, for state of interest $\rho =\sum\nolimits_{P\in {{{{\mathcal{P}}}}}_{n}}{c}_{P}P$ expanded in the Pauli basis, the purity may also be expanded as

28 $$\operatorname{Tr}(\;{\rho }^{2})=\sum\limits_{P,{P}^{{\prime} }\in {{{{\mathcal{P}}}}}_{n}}{c}_{P}{c}_{{P}^{{\prime} }}\operatorname{Tr}(P{P}^{{\prime} })=\sum\limits_{P\in {{{{\mathcal{P}}}}}_{n}}{c}_{P}^{2}{2}^{n}.$$

Here, the second equality is because the product of two non-identical Paulis is another Pauli, which is traceless. We will look at the contribution

29 $${C}_{k}:= \sum\limits_{P\in {{{{\mathcal{P}}}}}_{n}\atop w(P)=k}{c}_{P}^{2}{2}^{n},$$

and see how it is distributed over *k*, for different choices of *ρ*. These *ρ* correspond roughly to a state progressing through different layers of our circuit.

- **Computational basis state**. For *a* ∈ {0, 1}^*n*^, let
  30 $$\rho =\left\vert a\right\rangle \left\langle a\right\vert =\frac{1}{{2}^{n}}\sum\limits_{b\in {\{0,1\}}^{n}}{(-1)}^{a\cdot b}{Z}^{\;b},$$
  where *a* ⋅ *b* = *a*_1_*b*_1_ + *a*_2_*b*_2_ + … + *a*_*n*_*b*_*n*_ (mod 2). Then
  31 $$\operatorname{Tr}(\;{\rho }^{2})=\frac{1}{{2}^{n}}\sum\limits_{b\in {\{0,1\}}^{n}}1,$$
  where we have invoked the orthogonality of Pauli operators given by equation (17). Equation (31) shows that the only Pauli strings contributing to the purity are those containing *Z* without *X* and *Y*. This means that *C*_*k*_ is simply proportional to the total number of weight-*kn*-bit-strings, which is $$\left(\begin{array}{c}n\\ k\end{array}\right)/({2}^{n}-1)$$.
- **Pure product state**. A very similar story to the above holds in this case. We may decompose an *n*-qubit product state as
  32 $$\rho ={\rho }_{1}\otimes \cdots \otimes {\rho }_{n}=\sum\limits_{k}\sum\limits_{P\in {{{{\mathcal{P}}}}}_{n}:w(P)=k}{c}_{P}P,$$
  where for *P* such that *w*(*P*) = *k*, ${c}_{P}={\prod}_{i = 1}^{k}\operatorname{Tr}({P}_{i}{\rho }_{i})$ is a *k*-fold product. In particular, suppose that for each *ρ*_*i*_, the distribution over Paulis is bounded away from uniform in the following sense: there exists some Pauli $Q\in {{{\mathcal{P}}}}\setminus {\mathbb{I}}$ such that the coefficient of *Q* in *ρ*_*i*_ is at least 1 − *ϵ*. Then, for product states, the contribution of weight-*k* Pauli strings to the purity is approximately also $$\left(\begin{array}{c}n\\ k\end{array}\right)/({2}^{n}-1)$$.
- **State acted on by a noiseless Clifford two-design**. We consider the expected purity after the two-design acts. We see that for any initial state
  33 $$\rho=\frac{1}{{2}^{n}}{\mathbb{I}}+\sum\limits_{P\in {{{{\mathcal{P}}}}}_{n}\setminus {\mathbb{1}}}{c}_{P}P,$$
  this is
  34 $$\begin{aligned}\mathop{{\mathbb{E}}}\limits_{{{{\mathcal{C}}}} \sim {{{\mathcal{E}}}}}[\operatorname{Tr}({{{\mathcal{C}}}}{(\;\rho )}^{2})]&={{\mathbb{E}}}_{{{{\mathcal{C}}}} \sim {{{\mathcal{E}}}}}\left[{{\mathrm{Tr}}}\,{\left(\sum\limits_{P\in {{{{\mathcal{P}}}}}_{n}}{c}_{P}{{{\mathcal{C}}}}(P)\right)}^{2}\right]\\ &={{\mathbb{E}}}_{{{{\mathcal{C}}}} \sim {{{\mathcal{E}}}}}\left[\operatorname{Tr}\left(\sum\limits_{P\in {{{{\mathcal{P}}}}}_{n}}{c}_{P}^{2}{{{\mathcal{C}}}}(P){{{\mathcal{C}}}}(P)\right)\right]\\ &=\operatorname{Tr}\left[\left({\mathbb{1}}\frac{1}{{4}^{n}}+\sum\limits_{P\in {{{{\mathcal{P}}}}}_{n}\setminus {\mathbb{1}}}{c}_{P}^{2}\;\mathop{{\mathbb{E}}}\limits_{{{{\mathcal{C}}}} \sim {{{\mathcal{E}}}}}[{{{\mathcal{C}}}}(P){{{\mathcal{C}}}}(P)]\right)\right],\end{aligned}$$
  and, hence,
  35 $$\begin{aligned}\mathop{{\mathbb{E}}}\limits_{{{{\mathcal{C}}}} \sim {{{\mathcal{E}}}}}[\operatorname{Tr}({{{\mathcal{C}}}}{(\;\rho )}^{2})]&=\frac{1}{{2}^{n}}+\sum\limits_{P\in {{{{\mathcal{P}}}}}_{n}\setminus {\mathbb{1}}}{c}_{P}^{2}\operatorname{Tr}\left(\sum\limits_{Q\in {{{{\mathcal{P}}}}}_{n}\setminus {\mathbb{1}}}\frac{1}{{4}^{n}-1}Q\cdot Q\right)\\ &=\frac{1}{{2}^{n}}+\sum\limits_{k=1}^{n}\sum\limits_{Q\in {{{{\mathcal{P}}}}}_{n}\setminus {\mathbb{1}}:\atop w(Q)=k}\frac{1}{{4}^{n}-1}\left(\sum\limits_{P\in {{{{\mathcal{P}}}}}_{n}\setminus {\mathbb{1}}}{c}_{P}^{2}\right){2}^{n}.\end{aligned}$$
  Here, the second-last equality follows from the Pauli mixing property of Clifford two-designs, which says that any non-identity *P* gets mapped by such an ensemble to a uniform distribution over non-identity Paulis. This means that, in contrast to the above two scenarios, the contribution of weight-*k* Pauli strings to the purity $\operatorname{Tr}({{\mathbb{E}}}_{{{{\mathcal{C}}}} \sim {{{\mathcal{E}}}}}{[{{{\mathcal{C}}}}(\;\rho )]}^{2})$ is proportional to the number of weight-*k* Paulis there are, which is $$\left(\begin{array}{c}n\\ k\end{array}\right){3}^{k}/({4}^{n}-1)$$. Higher-weight Paulis contribute more.
- **State acted on by a noisy Clifford two-design**.
  36 $$\begin{array}{ll}\mathop{{\mathbb{E}}}\limits_{{{{\mathcal{C}}}} \sim {{{\mathcal{E}}}}}[\operatorname{Tr}({{{\Phi }}}_{p}\circ {{{\mathcal{C}}}}{(\;\rho )}^{2})]&={{\mathbb{E}}}_{{{{\mathcal{C}}}} \sim {{{\mathcal{E}}}}}\left[\operatorname{Tr}\left({\left(\frac{1}{{2}^{n}}{\mathbb{I}}+\frac{1}{{4}^{n}-1}\sum\limits_{P\in {{{{\mathcal{P}}}}}_{n}\setminus {\mathbb{I}}}{c}_{P}{p}^{w({{{\mathcal{C}}}}(P))}{{{\mathcal{C}}}}(P)\right)}^{2}\right)\right]\\ &={{\mathbb{E}}}_{{{{\mathcal{C}}}} \sim {{{\mathcal{E}}}}}\left[\operatorname{Tr}\left(\frac{1}{{4}^{n}}{\mathbb{I}}+{\left(\frac{1}{{4}^{n}-1}\right)}^{2}\sum\limits_{P\in {{{{\mathcal{P}}}}}_{n}\setminus {\mathbb{1}}}{c}_{P}^{2}{p}^{2w({{{\mathcal{C}}}}(P))}I\right)\right]\\&=\frac{1}{{2}^{n}}+{\left(\frac{1}{{4}^{n}-1}\right)}^{2}{2}^{n}\sum\limits_{P\in {{{{\mathcal{P}}}}}_{n}\setminus {\mathbb{1}}}{c}_{P}^{2}\;{{\mathbb{E}}}_{{{{\mathcal{C}}}} \sim {{{\mathcal{E}}}}}\left[\;{p}^{2w({{{\mathcal{C}}}}(P))}\right]\\ &\approx \frac{1}{{2}^{n}}\left(1+\frac{1}{{4}^{n}-1}\sum\limits_{P\in {{{{\mathcal{P}}}}}_{n}\setminus {\mathbb{1}}}{c}_{P}^{2}\;{{\mathbb{E}}}_{{{{\mathcal{C}}}} \sim {{{\mathcal{E}}}}}\left[\;{p}^{2w({{{\mathcal{C}}}}(P))}\right]\right).\end{array}$$
  By the Pauli mixing property, we find
  37 $$\begin{aligned}{{\mathbb{E}}}_{{{{\mathcal{C}}}} \sim {{{\mathcal{E}}}}}\left[\;{p}^{2w({{{\mathcal{C}}}}(P))}\right]&=\sum\limits_{i=1}^{{4}^{n}-1}\frac{1}{{4}^{n}-1}{p}^{2w({Q}_{i})}\\ &=\sum\limits_{k=1}^{n}\sum\limits_{Q\in {{{{\mathcal{P}}}}}_{n}\setminus {\mathbb{1}}:\atop w(Q)=k}\frac{1}{{4}^{n}-1}{p}^{2k},\end{aligned}$$
  implying that
  38 $$\begin{array}{l}{{\mathbb{E}}}_{{{{\mathcal{C}}}} \sim {{{\mathcal{E}}}}}\left[\operatorname{Tr}({{{\varPhi }}}_{p}\circ {{{\mathcal{C}}}}{(\;\rho )}^{2})\right]\\\approx \frac{1}{{2}^{n}}\left(1+\frac{1}{{4}^{n}-1}\sum\limits_{P\in {{{{\mathcal{P}}}}}_{n}\setminus {\mathbb{1}}}{c}_{P}^{2}\sum\limits_{k=1}^{n}\sum\limits_{Q\in {{{{\mathcal{P}}}}}_{n}\setminus {\mathbb{1}}:\atop w(Q)=k}\frac{1}{{4}^{n}-1}{p}^{2k}\right).\end{array}$$
  In particular, we see that all weight-*k* Pauli strings have exactly equal weights in the Pauli basis expansion of the state after the two-design, but now this weight is damped by a factor exponential with the Hamming weight *k*. That is, the contribution of all weight-*k* Paulis is proportional to $$\left(\begin{array}{c}n\\ k\end{array}\right){3}^{k}/({4}^{n}-1){p}^{2k}$$.

## Error mitigation: the lay of the land

The aim of an error-mitigation procedure is to produce a representation of the output of a noiseless quantum circuit given access to the actual noisy quantum device. Before we formally define different error-mitigation tasks, we outline the model for noisy quantum circuits that is used throughout this work. We consider quantum circuits of depth *D* acting on *n* qubits. In the quantum channel picture, such circuits take the form

39 $${{{\mathcal{C}}}}={{{{\mathcal{C}}}}}^{(D)}\circ \cdots \circ {{{{\mathcal{C}}}}}^{(2)}\circ {{{{\mathcal{C}}}}}^{(1)},$$

where each ${{{{\mathcal{C}}}}}^{(1)},\ldots ,{{{{\mathcal{C}}}}}^{(D)}$ denotes a layer of unitary quantum gates. We will then assume that in the noisy version, a quantum channel will act after each unitary we implement. That is, for such a circuit, we take its noisy version to be

40 $${{{\varPhi }}}_{{{{\mathcal{C}}}},{{{{\mathcal{N}}}}}^{(D)},\ldots, {{{{\mathcal{N}}}}}^{(1)}}={{{{\mathcal{N}}}}}^{(D)}\circ {{{{\mathcal{C}}}}}^{(D)}\circ \cdots \circ {{{{\mathcal{N}}}}}^{(2)}\circ {{{{\mathcal{C}}}}}^{(2)}\circ {{{{\mathcal{N}}}}}^{(1)}\circ {{{{\mathcal{C}}}}}^{(1)},$$

where ${{{{\mathcal{N}}}}}^{(1)},{{{{\mathcal{N}}}}}^{(2)},\ldots ,{{{{\mathcal{N}}}}}^{(D)}$ are quantum channels that describe the noise.

For simplicity, we will often consider the case in which every layer has an identical noise channel acting on it. In that case, we will denote the noisy version of the circuit as ${{{\varPhi }}}_{{{{\mathcal{C}}}},{{{\mathcal{N}}}}}$ or, if the noise channel ${{{\mathcal{N}}}}$ can be described by a parameter *p*, ${{{\varPhi }}}_{{{{\mathcal{C}}}},p}$. However, our results extend to when the noise is not uniform or not unital. For pedagogical reasons, our results are initially derived for local depolarizing noise and then extended to other noise models.

### Error-mitigation setting

The protocols listed in Supplementary Information Section I are but a slice of the wealth of error-mitigation techniques that have so far been proposed. In this section, we aim to unify all of these techniques by introducing the model of error mitigation that we will be using in the rest of this work. As explained in Supplementary Information Section I, lower bounds against the model imply lower bounds for many of those protocols. We will take the input of an error-mitigation protocol to be the following.

#### Definition 4

(Resources for error mitigation) *An error-mitigation algorithm predicts properties of the noiseless output state of a quantum circuit* ${{{\mathcal{C}}}}(\rho )$ *given the following elements.*

- *Classical descriptions of* ${{{\mathcal{C}}}}$ *and the noise channel* ${{{\mathcal{N}}}}$ *acting on* ${{{\mathcal{C}}}}$.
- *(Optional) A classical description of the input state to the circuit, ρ. If the algorithm utilizes this classical description, we call the algorithm input-state aware. If the algorithm does not utilize this classical description, we call it input-state agnostic.*
- *The ability to perform collective quantum measurements on several copies of the noisy circuit output state* ${{{\varPhi }}}_{{{{\mathcal{C}}}},{{{\mathcal{N}}}}}(\rho )$ *(see the remarks below on randomized families of circuits).*

We now make several remarks about this definition. First, our goal is to demarcate the information-theoretic limits of error mitigation. This we do by studying the sample complexity of error mitigation in our model. How many copies of the noisy output state are required to achieve the desired error-mitigation output? We rigorously quantify how this number scales in the complexity of ${{{\mathcal{C}}}}$ and the noise ${{{\mathcal{N}}}}$. Note that sample complexity lower-bounds computational complexity, and so lower bounds for sample complexity are also lower bounds for computational complexity.

Second, the practical scope of our model is broader than meets the eye. It encompasses even those protocols that run several different circuits with various levels of noise or take as additional input ‘training data’, which are pairs of (experimental) noisy and (simulated) noiseless expectation values for different circuits. In this case, we can always identify a representative circuit or noise level in the class of circuits that may be run. It is this circuit whose parameters will determine the complexity of error mitigation. The reader is referred to the discussion in Supplementary Information Section I for more details.

Third, we start in Supplementary Information Section II by proving lower bounds against input-state-agnostic protocols, and then in Supplementary Information Section II.B, we extend the results to input-state-aware protocols. Input-state-agnostic protocols are a natural starting point because they cannot possibly work by simulating the circuit ${{{\mathcal{C}}}}$, no matter how shallow that circuit is, simply because they do not know the input to the circuit. In fact, many existing error-mitigation protocols in the literature are already covered by the input-state-agnostic model because the classical description of the input state is not a parameter in the protocol.

Fourth, note that we have allowed for the ability to perform arbitrary collective measurements. This requires access to a quantum memory, a non-trivial quantum resource that might be out of reach. Because of this, most error-mitigation protocols in the literature do not require this ability. However, our conclusions hold even for algorithms that have such additional power.

Error mitigation is not an end in itself. Typically, it is used as the last step in a pipeline to solve some (quantum) computational task. Depending on what kind of task that is, different outputs of the mitigation procedure are required. Arguably, the most popular intended applications for near-term quantum computers are variational quantum algorithms^11,43–48^ where a quantum state is prepared through a parametrized quantum circuit and the parameters are iteratively adjusted to optimize a function *L*(〈*O*_1_〉, 〈*O*_2_〉, …) of expectation values of operators evaluated on said state. The archetypal variational quantum algorithm is the variational quantum eigensolver^11,43,44^ where the function

41 $$L(\left\vert \psi \right\rangle )=\sum\limits_{i}\left\langle \psi \right\vert {O}_{i}\left\vert \psi \right\rangle =\sum\limits_{i}\langle {O}_{i}\rangle$$

is the expectation value of a Hamiltonian *H* = ∑_*i*_*O*_*i*_. In this case, the ground state of *H* yields the solution to the optimization problem, and so the optimized parametrized quantum circuit should ideally prepare a state close to the ground state of *H*. However, such circuits are noisy, and the goal of error mitigation is to correct this. It stands to reason then that an error-mitigation protocol should output estimates of the expectation values 〈*O*_*i*_〉 for the state output by the noiseless version of these circuits. We can formally capture this task in the following definition of weak error mitigation. In all the definitions below, let $\left\vert \psi \right\rangle ={{{\mathcal{C}}}}(\;\rho )$ be the state vector output by the noiseless circuit.

#### Definition 5

(Weak error mitigation (expectation value error mitigation)) *An* (*ϵ*, *δ*) *weak error-mitigation algorithm* ${{{\mathcal{A}}}}$ *with resources as in* *Definition* *4**takes as input a classical description of a set of observables* ${{{\mathcal{M}}}}=\{{O}_{1},\ldots ,{O}_{\ell }\}$ *satisfying* ∥*O*_*i*_∥ ≤ 1 *and outputs estimates* ${\hat{o}}_{i}$ *of* ${o}_{i}=\left\langle \psi \right\vert {O}_{i}\left\vert \psi \right\rangle$ *such that*

42 $${\mathbb{P}}\left[| {\hat{o}}_{i}-{o}_{i}| \le \epsilon \,{{\mbox{for all}}}\,1\le i\le \ell \right]\ge 1-\delta .$$

Here, the probability in equation (42) is over the randomness of the error-mitigation algorithm. This randomness could come from using classical random bits or from making measurements of the quantum states available as input.

The task of computing expectation values is ubiquitous in near-term quantum computing. Most error-mitigation algorithms in the literature address the weak error-mitigation task, including all protocols listed in Supplementary Information Section I. However, in some applications, knowing expectation values is not sufficient. In these cases, we would like to represent the strongest possible access, on par with access to the quantum computer, which is sampling the circuit’s output. This leads us to a definition of strong error mitigation.

#### Definition 6

(Strong error mitigation (sample error mitigation)) *An* (*ϵ*, *δ*) *strong error-mitigation algorithm* ${{{\mathcal{A}}}}$ *with resources as in* *Definition* *4**outputs a bit-string z* *sampled according to a distribution z* ~ *μ* *such that with probability* 1 − *δ*,

43 $${d}_{{{{\rm{TV}}}}}\left(\;\mu ,{D}_{\left\vert \psi \right\rangle }\right)\le \epsilon, \qquad \,({\mbox{additive error}}\,\epsilon)$$

*or alternatively*

44 $$\frac{{D}_{\left\vert \psi \right\rangle }(z)}{\mu (z)}\le \kappa \,{{\mbox{for all}}}\,z\in {\{0,1\}}^{n},\qquad \,({\mbox{multiplicative error}}\,\kappa)$$

*where* ${D}_{\left\vert \psi \right\rangle }$ *is the distribution arising from a computational basis measurement of* $\left\vert \psi \right\rangle$.

As the strong error-mitigation task is more difficult than the weak error-mitigation task (as we will show below, strong error mitigation implies weak error mitigation), and weak error mitigation is usually sufficient for near-term applications, there are fewer methods available that achieve this, an example being virtual distillation^13,14^. The two notions of error are related, as *κ* multiplicative error implies *ϵ* = (1 − 1/*κ*)^1/2^ additive error. To see this, note that the multiplicative error requirement can be rewritten as

45 $$\frac{{D}_{\left\vert \psi \right\rangle }(z)}{\mu (z)}\le \kappa \to {D}_{\infty }\left({D}_{\left\vert \psi \right\rangle }\parallel \mu\right)\le \log (\kappa ).$$

By the monotonicity of relative entropies, we then have

46 $${D}_\mathrm{KL}\left({D}_{\left\vert \psi \right\rangle }\parallel \mu\right)={D}_{1}\left({D}_{\left\vert \psi \right\rangle }\parallel \mu\right)\le {D}_{\infty }\left({D}_{\left\vert \psi \right\rangle }\parallel \mu\right)\le \log (\kappa ).$$

By the Bretagnolle–Huber inequality, we can then relate this to the total variation distance as

47 $${d}_\mathrm{TV}\left({D}_{\left\vert \psi \right\rangle },\mu\right)\le \sqrt{1-\exp\left(-{D}_\mathrm{KL}\left({D}_{\left\vert \psi \right\rangle }\parallel \mu\right)\right)}\le \sqrt{1-\frac{1}{\kappa }}.$$

On the other hand, it is easy to check that additive error does not imply multiplicative error for any setting of the parameters.

The additive-error requirement for strong error mitigation makes intuitive sense when sampling access to the noiseless quantum state is required and it is not important that the samples generated by the error-mitigation algorithm should come from any particular subset of the support. However, this is not the case for some of the near-term quantum algorithms for which one might want to mitigate errors. Consider, for example, variational quantum optimization algorithms like the quantum approximate optimization problem^12^, where a diagonal Hamiltonian ${{{\mathcal{H}}}}$ encodes a hard combinatorial optimization problem. Here, computational basis states of low energy correspond to approximate solutions of the combinatorial optimization problem. If the noiseless state $\left\vert \psi \right\rangle$ has an overlap of *O*(1/poly(*n*)) with the low-energy subspace of ${{{\mathcal{H}}}}$, polynomially many samples from the noiseless distribution ${D}_{\left\vert \psi \right\rangle }$ are sufficient to solve the optimization problem with high probability. The same would then hold for sampling from an error-mitigated distribution with multiplicative error *κ* = *O*(1/poly(*n*)). Such a distribution must also have an inversely polynomially small weight on the set of low-energy strings. Such important examples motivate our definition of multiplicative error mitigation.

### Relationship between notions of error mitigation

Having established that a comprehensive study of error mitigation must consider both weak and strong versions, we now ask how they are related. We are particularly interested in understanding whether the output of one kind of error mitigation can be used, in polynomial time, to compute the output of another kind of error mitigation. A first observation is that strong error mitigation implies weak error mitigation with local observables. Let ${{{\mathcal{A}}}}$ be a strong error-mitigation algorithm. Definition 6 requires ${{{\mathcal{A}}}}$ to output a sample from the computational basis of the noiseless circuit’s output state. However, if *O*_*i*_ is a local observable (say, a product Pauli observables) and we assume that the cost of strong error mitigation on ${{{\mathcal{C}}}}$ does not increase substantially by appending a layer of product unitaries to ${{{\mathcal{C}}}}$, then ${{{\mathcal{A}}}}$ can also output samples from the probability distribution associated with measuring ${{{\mathcal{C}}}}$ in an eigenbasis of *O*_*i*_. After applying the strong error-mitigation procedure to obtain enough clean samples from the eigenbasis of *O*_*i*_, we can estimate the expectation value $\operatorname{Tr}({O}_{i}{{{\mathcal{C}}}}(\rho ))$ empirically to a desired precision, thereby achieving weak error mitigation. One of the main questions asked in this work is whether we can hope for the other direction to hold. That is, whether weak error-mitigation protocols can also be used to obtain samples from a noiseless circuit. Supplementary Information Section V answers this question.

## Outline of the proof of Theorem 1

To prove Theorem 1, and more generally, Supplementary Information Theorem 4, we engineer a family of quantum circuits ${{{\mathcal{C}}}}$ that converge very quickly to the maximally mixed state under noise, so that $D({{{{\mathcal{C}}}}}^{{\prime} }(\left\vert z\right\rangle \left\langle z\right\vert )\parallel {\mathbb{I}}/{2}^{n})\le n+\log \operatorname{Tr}({{{{\mathcal{C}}}}}^{{\prime} }{(\left\vert z\right\rangle \left\langle z\right\vert )}^{2})\le {p}^{nD}$. Figure 1 illustrates the intuition behind our construction. A toy model of our construction is a circuit consisting of alternating noiseless two-designs and depolarizing noise. The two-designs are from an ensemble of Clifford circuits, which is Pauli mixing. This means that it maps each Pauli to a uniformly random Pauli. Intuitively, such random circuits spread entanglement very fast, as such a uniform distribution puts significant weight on the set of higher Hamming weight, that is, more non-local, Paulis.

However, this renders such circuits more sensitive to noise. Our proof illustrates this phenomenon quantitatively by tracking the evolution of the purity $\operatorname{Tr}(\;{\rho }^{2})$ of some state *ρ* progressing through our circuit. Expanding the purity in terms of the Pauli basis and studying the distribution over contributions by Paulis (grouped by Hamming weight), we see that applying a Pauli mixing circuit shifts the distribution towards higher-weight Paulis. Such Paulis, however, are damped more by the next layer of depolarizing noise, as depolarizing noise causes the coefficient of the Pauli expansion to decay exponentially with its Hamming weight. We then iterate the argument for every additional layer of a two-design followed by noise. Formal definitions of the Pauli mixing property as well as a rigorous version of this argument that also deals with noise within the two-designs are provided in the Supplementary Information.

## Online content

Any methods, additional references, Nature Portfolio reporting summaries, source data, extended data, supplementary information, acknowledgements, peer review information; details of author contributions and competing interests; and statements of data and code availability are available at 10.1038/s41567-024-02536-7.

## Supplementary information

Supplementary Information Further details of the arguments presented in the main text and Methods. It does not provide data or code. It contains one additional figure.

## Data Availability

No data has been used to support the findings of this manuscript.

## References

[1] Feynman, R. P. Quantum mechanical computers. *Found. Phys.* **16**, 507 (1986).

[2] Shor, P. W. Algorithms for quantum computation: discrete logarithms and factoring. In *Proc. 35th Annual Symposium on Foundations of Computer Science* (ed. Goldwasser, S.) 124–134 (IEEE, 1994).

[3] Shor, P. W. Scheme for reducing decoherence in quantum computer memory. *Phys. Rev. A* **52**, R2493 (1995). 9912632 10.1103/physreva.52.r2493

[4] Gottesman, D. in *Quantum Information Science and Its Contributions to Mathematics* (ed. Lomonaco Jr, S. J.) 13–60 (AMS, 2010).

[5] Campbell, E. T., Terhal, B. M. & Vuillot, C. Roads towards fault-tolerant universal quantum computation. *Nature* **549**, 172 (2017). 28905902 10.1038/nature23460

[6] Li, Y. & Benjamin, S. C. Efficient variational quantum simulator incorporating active error minimization. *Phys. Rev. X* **7**, 021050 (2017).

[7] Temme, K., Bravyi, S. & Gambetta, J. M. Error mitigation for short-depth quantum circuits. *Phys. Rev. Lett.* **119**, 180509 (2017). 29219599 10.1103/PhysRevLett.119.180509

[8] Endo, S., Benjamin, S. C. & Li, Y. Practical quantum error mitigation for near-future applications. *Phys. Rev. X* **8**, 031027 (2018).

[9] Cai, Z. et al. Quantum error mitigation. *Rev. Mod. Phys*. **95**, 045005 (2023).

[10] van den Berg, E., Minev, Z. K., Kandala, A. & Temme, K. Probabilistic error cancellation with sparse Pauli–Lindblad models on noisy quantum processors. *Nat. Phys.* **19**, 1116 (2023).

[11] McClean, J. R., Romero, J., Babbush, R. & Aspuru-Guzik, A. The theory of variational hybrid quantum-classical algorithms. *New J. Phys.* **18**, 023023 (2016).

[12] Farhi, E., Goldstone, J. & Gutmann, S. A quantum approximate optimization algorithm. Preprint at 10.48550/arXiv.1411.4028 (2014).

[13] Huggins, W. J. et al. Virtual distillation for quantum error mitigation. *Phys. Rev. X* **11**, 041036 (2021).

[14] Koczor, B. Exponential error suppression for near-term quantum devices. *Phys. Rev. X* **11**, 031057 (2022).

[15] Czarnik, P., Arrasmith, A., Coles, P. J. & Cincio, L. Error mitigation with Clifford quantum-circuit data. *Quantum* **5**, 592 (2022).

[16] Takagi, R., Endo, S., Minagawa, S. & Gu, M. Fundamental limits of quantum error mitigation. *npj Quantum Inf.* **8**, 114 (2022).

[17] Takagi, R., Tajima, H. & Gu, M. Universal sample lower bounds for quantum error mitigation. *Phys. Rev. Lett.* **131**, 210602 (2023). 10.1103/PhysRevLett.131.210602 38072595

[18] Tsubouchi, K., Sagawa, T. & Yoshioka, N. Universal cost bound of quantum error mitigation based on quantum estimation theory. *Phys. Rev. Lett*. **131**, 210601 (2023). 10.1103/PhysRevLett.131.210601 38072608

[19] Deshpande, A. et al. Tight bounds on the convergence of noisy random circuits to the uniform distribution. *PRX Quantum* **3**, 040329 (2022).

[20] Müller-Hermes, A., Franca, D. S. & Wolf, M. M. Relative entropy convergence for depolarizing channels. *J. Math. Phys.* **57**, 2 (2016).

[21] Stilck França, D. & García-Patrón, R. Limitations of optimization algorithms on noisy quantum devices. *Nat. Phys.* **17**, 1221 (2021).

[22] Tsybakov, A. B. *Introduction to Non-Parametric Estimation* (Springer, 2009).

[23] De Palma, G., Marvian, M., Rouzé, C. & Stilck Franca, D. Limitations of variational quantum algorithms: a quantum optimal transport approach. *PRX Quantum* **4**, 010309 (2023).

[24] Wang, S. et al. Can error mitigation improve trainability of noisy variational quantum algorithms? *Quantum* **8**, 1287 (2024).

[25] Thanasilp, S., Wang, S., Cerezo, M. & Holmes, Z. Exponential concentration and untrainability in quantum kernel methods. Preprint at 10.48550/arXiv.2208.11060 (2022).

[26] Wang, S. et al. Noise-induced barren plateaus in variational quantum algorithms. *Nat. Commun.* **12**, 6961 (2021). 34845216 10.1038/s41467-021-27045-6 PMC8630047

[27] Deshpande, A. et al. Tight bounds on the convergence of noisy random circuits to the uniform distribution. *PRX Quantum* **3**, 040329 (2022).

[28] Cleve, R., Leung, D., Liu, L. & Wang, C. Near-linear constructions of exact unitary 2-designs. *Quantum Inf. Comput.* **16**, 721–756 (2016).

[29] Reyzin, L. Statistical queries and statistical algorithms: foundations and applications. Preprint at https://arxiv.org/abs/2004.00557 (2020).

[30] Yatracos, Y. G. Rates of convergence of minimum distance estimators and Kolmogorov’s entropy. *Ann. Stat.* **13**, 768 (1985).

[31] Blum, A. et al. Weakly learning DNF and characterizing statistical query learning using Fourier analysis. In *Proc. 26th Annual ACM Symposium on Theory of Computing* 253–262 (ACM, 1994).

[32] França, D. S., Strelchuk, S. & Studziński, M. Efficient classical simulation and benchmarking of quantum processes in the Weyl basis. *Phys. Rev. Lett.* **126**, 210502 (2021). 34114840 10.1103/PhysRevLett.126.210502

[33] Rall, P., Liang, D., Cook, J. & Kretschmer, W. Simulation of qubit quantum circuits via Pauli propagation. *Phys. Rev. A* **99**, 062337 (2019).

[34] Bravyi, S., Kliesch, A., Koenig, R. & Tang, E. Obstacles to variational quantum optimization from symmetry protection. *Phys. Rev. Lett.* **125**, 260505 (2020). 33449785 10.1103/PhysRevLett.125.260505

[35] Farhi, E., Gamarnik, D. & Gutmann, S. The quantum approximate optimization algorithm needs to see the whole graph: worst case examples. Preprint at 10.48550/arXiv.2005.08747 (2020).

[36] Eldar, L. & Harrow, A. W. Local Hamiltonians whose ground states are hard to approximate. In *Proc. 58th Annual Symposium on Foundations of Computer Science* 427–438 (IEEE, 2017).

[37] Anshu, A., Breuckmann, N. P. & Nirkhe, C. NLTS Hamiltonians from good quantum codes. In *Proc. 55th Annual ACM Symposium on Theory of Computing* 1090–1096 (ACM, 2023).

[38] González-Garcıa, G., Trivedi, R. & Cirac, J. I. Error propagation in NISQ devices for solving classical optimization problems. *PRX Quantum* **3**, 040326 (2022).

[39] Mosonyi, M. & Hiai, F. On the quantum Rényi relative entropies and related capacity formulas. *IEEE Trans. Inf. Theory* **57**, 2474 (2011).

[40] Dankert, C., Cleve, R., Emerson, J. & Livine, E. Exact and approximate unitary 2-designs and their application to fidelity estimation. *Phys. Rev. A* **80**, 012304 (2009).

[41] Gottesman, D. *Stabilizer Codes and Quantum Error Correction* (Caltech, 1997).

[42] Watrous, J. *The Theory of Quantum Information* (Cambridge Univ. Press, 2018).

[43] Cerezo, M. et al. Variational quantum algorithms. *Nat. Rev. Phys.* **3**, 625 (2021).

[44] Bharti, K. et al. Noisy intermediate-scale quantum algorithms. *Rev. Mod. Phys.* **94**, 015004 (2022).

[45] Kearns, M. Efficient noise-tolerant learning from statistical queries. *J. ACM* **45**, 983–1006 (1998).

[46] Hinsche, M. et al. A single *T*-gate makes distribution learning hard. *Phys. Rev. Lett.* **130**, 240602 (2023). 37390441 10.1103/PhysRevLett.130.240602

[47] Arunachalam, S., Grilo, A. B. & Yuen, H. Quantum statistical query learning. Preprint at 10.48550/arXiv.2002.08240 (2020).

[48] França, D. S. & Garcia-Patron, R. A game of quantum advantage: linking verification and simulation. *Quantum* **6**, 753 (2022).

