# Supplementary material for: Exponentially tighter bounds on limitations of quantum error mitigation
Source: Nat Phys. 2024 Jul 25;20(10):1648–58. doi: 10.1038/s41567-024-02536-7 (PMC11473368; doi:10.1038/s41567-024-02536-7)
Supplement: Supplementary file 1 — Further details of the arguments presented in the main text and Methods. It does not provide data or code. It contains one additional figure. [file 41567_2024_2536_MOESM1_ESM.pdf]

nature physics

Supplementary information

https://doi.org/10.1038/s41567-024-02536-7

Exponentially tighter bounds on limitations of quantum error mitigation

In the format provided by the authors and unedited

Supplemental Material: Exponentially tighter bounds on limitations of quantum error mitigation

This supplemental material provides substantial detail of the arguments presented in the main text and in the Methods section.

- We start with Section I discussing practical error mitigation protocols and related work.
- Section II presents our conceptual contribution. Here we prove the 'appetizer' Theorem 1 which studies the limitations of error mitigation under depolarizing noise. While this appetizer theorem implies a weaker scaling of error mitigation complexity as compared to our main theorems, we elect to start here as the proof already introduces the conceptual 'outer loop' that we will use in the rest of our theorems, namely the application of the generalized Fano's lemma to relate error mitigation to the noisy state discrimination problem.
- In Section III, we present our main theorem (Theorem 3), and fill out the technical 'inner loop' of our argument under depolarizing noise. The proof of Theorem 3 is our first technical contribution: the worst-case circuit ensemble that we construct, which has the property that the relative entropy of its average output state to the maximally-mixed state—and hence error mitigation's sample complexity—scales, in the worst case, exponentially in both n and D. We conclude the section by further refining our results so that they hold for local circuits and even lower depths than before.
- Section IV contains our second technical contribution: we extend all our previous conclusions to error mitigation under a new class of noise models, non-unital noise, although under the toy model of local non-unital local noise interspersed by global $2-$designs. We arrive at the same conclusions in this toy model, the sample complexity for error mitigation is exponential.
- Finally, in Section V, we discuss the relationship between notions of error mitigation.

I. RELATION TO PRACTICAL ERROR MITIGATION PROTOCOLS AND RELATED WORK

In this section, we show that our mathematical framework describes many broad classes of error mitigation protocols used in practice, by showing that the same line of argument that we use in the main text can also be applied, with small adaptations, to these other protocols. We also briefly mention related attempts in the literature to rigorously analyze the sample complexity of error mitigation. We will list these classes, briefly explain how they work, and describe to what our extent our results apply to them. In all of the following, $\mathcal{C}'$ refers to a noisy version of the circuit $\mathcal{C}$, $\omega_{\mathcal{C},\mathcal{N}}$ refers to the output state of the circuit affected by noise, while $|\psi\rangle$ refers to the output state vector of the noiseless circuit.

The key interface between the specific workings of any class of algorithms, and our mathematical machinery, is a relative entropy quantity defined on various output probability distributions of those algorithms run on different inputs. The following Lemma, which we will use extensively in this section and hence refer to as the 'central lemma', captures this rigorously. The central lemma is proven by using exactly the same mapping from noisy state discrimination to error mitigation that we use to prove Theorem 1, and invoking the *generalized Fano method for multiple hypothesis testing* [1] to the former problem.

Lemma 1 (Central lemma)**.** *Fix a class of input state-agnostic weak error mitigation algorithms. Suppose there is a set of states $\{\rho_i\}_{i=0}^N$ and a circuit $\mathcal{C}$, such that if we let P_i be the distribution over the outputs of any algorithm in that class, run on m copies of the noisy circuit's output state $\mathcal{C}'(\rho_i)$, and that*

$$\frac{1}{N+1}\sum_{k=0}^{N} D(P_k\|P_N) \leq \log(N)\cdot m\cdot\beta, \tag{1}$$

then there exists a problem instance (namely, specified by $\mathcal{C}'$, $\{\rho_i\}_{i=0}^N$) on which no error mitigation algorithm in the class can succeed with less than $m = \Omega(\beta^{-1}(1-\delta))$ copies of the noisy circuit's output state.

Our framework can thus handle any protocol, for which we can show an upper-bound on the quantity on the left-hand-side of Eq. (1). We now proceed to list out well-known classes of error mitigation protocols for which we can show this:

1. *Virtual distillation* [2, 3]: This protocol takes as input k copies of the noisy circuit output state $\omega_{\mathcal{C},\mathcal{N}}$ and outputs an estimate of

$$\langle O\rangle_{\text{corrected}} = \frac{\mathrm{Tr}(O\omega^k_{\mathcal{C},\mathcal{N}})}{\mathrm{Tr}(\omega^k_{\mathcal{C},\mathcal{N}})} \quad . \tag{2}$$

as its approximation of the ideal $\langle O\rangle = \mathrm{Tr}(O|\psi\rangle\langle\psi|)$. The intention of exponentiating the noisy state k times, is to amplify the dominant eigenvector in the eigen-decomposition of $\omega_{\mathcal{C},\mathcal{N}}$, as under some assumptions, this dominant eigenvector has a large overlap with the noiseless output state vector $|\psi\rangle\langle\psi|$. The protocol is known as 'virtual' distillation because the quantity in Eq. (2) can be estimated without ever preparing the state $\omega^k_{\mathcal{C},\mathcal{N}}/\mathrm{Tr}(\omega^k_{\mathcal{C},\mathcal{N}})$.

Concretely, the numerator and denominator of Eq. (2) are often estimated by applying a short quantum circuit to post-process the k copies of $\omega_{\mathcal{C},\mathcal{N}}$, measuring it and then applying classical post-processing. For example, Ref. [2] shows that for certain observables O, it suffices to apply a single-layer of two-qubit gates to k copies of $\omega_{\mathcal{C},\mathcal{N}}$, followed by a computational basis measurement. As remarked below the proof of Proposition 1, our proof goes through even when the error mitigation algorithm consists not only of the classical post-processing steps, but also manipulates the quantum states coherently before the measurement.

On the other hand, the procedure presented in Ref. [3] passes all k quantum states through a short circuit, and the probability of measuring an auxiliary system in the '0' state is related to the value of the numerator and denominator of Eq. (2). For circuits affected by unital

noise, Ref. [4] has shown that the probability of success of virtual distillation in this protocol decays exponentially both in the number of qubits and depth of the circuit. This work shows that the same holds for circuits that form a 2-design affected by non-unital noise. As we show in Section IV C, after we apply a 2-design followed by one layer of product noise, the purity of the system is already exponentially small in expectation. Since the success probability of virtual distillation is $\mathrm{Tr}(\omega_{\mathcal{C},\mathcal{N}}^k)$ which is upper-bounded by $\mathrm{Tr}(\omega_{\mathcal{C},\mathcal{N}}^2)$, the former will also be exponentially small when the latter is.

2. *Clifford data regression (CDR) and other learning-based schemes for error mitigation* [5–7]: Fix a circuit of interest $\mathcal{C}$, assumed to be not classically simulatable, which is to be error-mitigated. These protocols generally proceed by collecting 'training examples'

$$(X_i^{\text{noisy}}, X_i^{\text{exact}})_i,$$

which are expectation values of the observable on noisy and noiseless classically simulatable circuits related to $\mathcal{C}$. X_i^{noisy} are obtained via running the actual circuits; X_i^{exact} are obtained by classical simulation. One then makes the ansatz that X_i^{noisy} and X_i^{exact}, for classes of observables or circuits, are related via some functional relation—Ref. [5] uses a linear ansatz—and fits the parameters of the ansatz using the training pairs. Then, finally, given a noisy expectation value $\mathrm{Tr}(O\omega_{\mathcal{C},\mathcal{N}})$ measured on the actual circuit, the learned function is applied to obtain the noiseless value $\mathrm{Tr}(O|\psi\rangle\langle\psi|)$.

The techniques in this work suffice to bound the number of runs of $\mathcal{C}'$ that must be used in the last step, and we sketch how to do so in the following paragraph. (This of course also lower bounds the total number of circuit runs, including those used to generate the training examples.) We note however, that this last step is what we term 'input state-aware' – that is, the learned function must be applied to circuits initialized with the same input state as was used in the 'training' phase. Nevertheless, the conclusion of the central lemma holds, albeit with a slight reinterpretation, for input-state aware protocols. We elaborate on this framework for dealing with the input state dependence in Section II B.

Let $\mathcal{C}_1, \ldots, \mathcal{C}_r$ be the r distinct circuits that are run in the process of generating the training data, and suppose each circuit is run g times and measured after each run. Let Train be the algorithm that generates the training data—that is, Train takes in

$$\omega_{\mathcal{C}_1,\mathcal{N}}^{\otimes g} \otimes \omega_{\mathcal{C}_2,\mathcal{N}}^{\otimes g} \otimes \ldots \omega_{\mathcal{C}_r,\mathcal{N}}^{\otimes g}, \tag{3}$$

(here we do not specify the input to the circuits used to generate the training data because it is irrelevant), makes a quantum measurement on $\omega_{\mathcal{C}_1,\mathcal{N}}^{\otimes g}$ to generate X_i^{noisy} and classically simulates it to generate X_i^{exact}, for all i. Further, let $\mathcal{A}$ be the error mitigation algorithm that takes as input m copies of $\Phi_{\mathcal{C},\mathcal{N}}(\rho_k)$, and the training data (which is the output of Train), and outputs the expectation values $\hat{O} \in \mathbb{R}^m$ where $\hat{O}[i]$ is the estimate for $\langle\psi| O_i |\psi\rangle$. We denote the probability distribution of this algorithm over outputs $\hat{O}$ when the circuit input is $|k\rangle\langle k|$ as P_k. Then

$$D(P_k\|P_N) = D\left(\mathcal{A}\left(\Phi_{\mathcal{C},\mathcal{N}}(\rho_k)^{\otimes m} \otimes \mathsf{Train}(\omega_{\mathcal{C}_1,\mathcal{N}}^{\otimes g} \otimes \omega_{\mathcal{C}_2,\mathcal{N}}^{\otimes g} \otimes \ldots \omega_{\mathcal{C}_r,\mathcal{N}}^{\otimes g})\right) \| \mathcal{A}\left(\frac{\mathbb{I}}{2^n}^{\otimes m} \otimes \mathsf{Train}(\omega_{\mathcal{C}_1,\mathcal{N}}^{\otimes g} \otimes \omega_{\mathcal{C}_2,\mathcal{N}}^{\otimes g} \otimes \ldots \omega_{\mathcal{C}_r,\mathcal{N}}^{\otimes g})\right)\right). \tag{4}$$

$$\leq D\left(\Phi_{\mathcal{C},\mathcal{N}}(\rho_k)^{\otimes m} \otimes \mathsf{Train}(\omega_{\mathcal{C}_1,\mathcal{N}}^{\otimes g} \otimes \omega_{\mathcal{C}_2,\mathcal{N}}^{\otimes g} \otimes \ldots \omega_{\mathcal{C}_r,\mathcal{N}}^{\otimes g}) \middle\| \frac{\mathbb{I}}{2^n}^{\otimes m} \otimes \mathsf{Train}(\omega_{\mathcal{C}_1,\mathcal{N}}^{\otimes g} \otimes \omega_{\mathcal{C}_2,\mathcal{N}}^{\otimes g} \otimes \ldots \omega_{\mathcal{C}_r,\mathcal{N}}^{\otimes g})\right) \tag{5}$$

$$\leq mD\left(\Phi_{\mathcal{C},\mathcal{N}}(\rho_k) \middle\| \frac{\mathbb{I}}{2^n}\right) + D\left(\mathsf{Train}(\omega_{\mathcal{C}_1,\mathcal{N}}^{\otimes g} \otimes \omega_{\mathcal{C}_2,\mathcal{N}}^{\otimes g} \otimes \ldots \omega_{\mathcal{C}_r,\mathcal{N}}^{\otimes g}) \middle\| \mathsf{Train}(\omega_{\mathcal{C}_1,\mathcal{N}}^{\otimes g} \otimes \omega_{\mathcal{C}_2,\mathcal{N}}^{\otimes g} \otimes \ldots \omega_{\mathcal{C}_r,\mathcal{N}}^{\otimes g})\right)$$

$$\leq mD\left(\Phi_{\mathcal{C},\mathcal{N}}(\rho_k) \middle\| \frac{\mathbb{I}}{2^n}\right), \tag{6}$$

where the first inequality follows by data processing and the second follows from the tensorization of relative entropy. We conclude by plugging Eq. (6) into the central lemma (Lemma 1), using the bounds on $D(\Phi_{\mathcal{C},\mathcal{N}}(\rho_k)\|\frac{\mathbb{I}}{2^n})$ we have obtained in the main text, and plugging it all into Theorem 2 which pertains to input state aware protocols.

3. *Zero-noise extrapolation* (ZNE) [8]: Given the circuit $\mathcal{C}$ with noise strength λ, one runs it at n different *amplified* noise levels $c_j\lambda$ ($c_j > 1$), computes expectation values on each such circuit, and combines the noisy estimates into an estimate for the expectation value without noise by extrapolating the set of obtained expectation values to zero noise. Variations on the theme include considering other noise models and interpolation schemes [9–11].

We comment now on how our results apply to the proposal of Ref. [8]. The extension to other variations of this scheme should be similar. Here, to run a circuit at noise level $c\lambda$ where $c \geq 1$, each gate is run for a time cT, where $c = 1$ corresponds to the noise rate in the original circuit. Ref. [8] further prescribes that the final estimate should be computed as

$$\hat{E} = \sum_{j=1}^{n} \gamma_j \hat{E}(c_j\lambda) \quad , \tag{7}$$

where $\sum_{j=0}^{n} \gamma_j = 1$ and $\sum_{j=0}^{n} \gamma_j c_j^k = 0$ for $k = 1, \ldots, n$. If the evolution of the original circuit is

$$\frac{\partial}{\partial t}\rho = -i[K(t), \rho] + \lambda\mathcal{L}(\rho) \quad , \tag{8}$$

where $K(t)$ is the coherent circuit and $\mathcal{L}$ represents the noise we want to mitigate, then stretching all gate times by c has the effect of replacing $\lambda \leftarrow c\lambda$ for $c > 1$ in the above equation. In the quantum circuit picture, if we assume that each circuit is acted upon by local depolarizing noise with effective depolarizing parameter p after each gate, this has the effect of replacing the parameter $p \leftarrow p^c$. Let us assume that c is the minimum noise level at which the circuit is run. Indeed, c cannot be arbitrarily small, as in this case the circuit is noiseless and error mitigation would not be required. Then, letting P_k denote the probability distribution over $\hat{E}$ when the input to the circuits we have constructed is $|k\rangle\langle k|$, we apply a similar line of reasoning as in Eqs. (4) to (6) to obtain a relative entropy bound. In fact, our choice to compute these bounds with c taking its minimum value weakens them, as the higher noise levels used in practice result in even faster convergence to the identity.

4. *Probabilistic error cancellation* [8**?** , 9]: In this protocol, one rewrites a noiseless circuit $\mathcal{U}$ in terms of a linear combination of noisy circuits $\mathcal{F}_i$: $\mathcal{U} = \sum_i a_i \mathcal{F}_i$ where $a_i \in \mathbb{R}$ could be negative. Similarly, any desired expectation value of interest (of the observable M, say) can be decomposed as the convex combination

$$\mathrm{Tr}(M\mathcal{U}(\cdot)) = \sum_i a_i \,\mathrm{Tr}(M\mathcal{F}_i(\cdot)) = \gamma \sum_i p_i \,\mathrm{sign}(a_i)\, \mathrm{Tr}(M\mathcal{F}_i(\cdot)) \quad . \tag{9}$$

Then, with probability

$$p_i = \frac{|a_i|}{\sum_i |a_i|} \tag{10}$$

and $\gamma = \sum_i |a_i|$, $\mathrm{Tr}(M\mathcal{F}_i(\cdot))$ is estimated by running the noisy circuit $\mathcal{F}_i$ g times. This is repeated r times (each time sampling $\mathcal{F}_i$ anew from p), the results are averaged and the estimate is multiplied by γ.

Our analysis applies here too, because we can model the computation of the desired quantity (the right-hand-side of Eq. (9)) with a quantum channel $\mathcal{M}$, as follows: Consider the state

$$\omega_k^{\otimes r} = \bigotimes_{j=1}^{r} \left(\sum_i p_i |i\rangle\langle i|_{A_j} \otimes (\mathcal{F}_i(|k\rangle\langle k|)^{\otimes g})_{B_j} \right) \quad , \tag{11}$$

and the channel $\mathcal{M}$ which consists in 1) Applying $\bigotimes_j P_{A_j}$ on this state, where P_{A_j} is a projector on the classical system A_j; 2) On each of the r systems, measuring $\mathcal{M}^{\otimes g}$ on B_j and averaging the result; 3) Classically averaging the results across the r systems and multiplying by γ.

Then let the output of this channel be denoted by P_k, for $k \in \{0,1\}^n$, while let P_N denote $\mathcal{M}$ acting on the state

$$\sigma^{\otimes r} = \bigotimes_{j=1}^{r} \left(\sum_i p_i |i\rangle\langle i|_{A_j} \otimes \left(\frac{\mathbb{I}}{2^n}^{\otimes g} \right)_{B_j} \right) \quad . \tag{12}$$

Then we follow similar steps as before to obtain

$$\begin{aligned} D(P_k \| P_N) &= D\left(\mathcal{M}(\omega_k^{\otimes r}) \| \mathcal{M}\left(\sigma^{\otimes r}\right)\right) \leq rD\left(\omega_k \| \sigma\right) \\ &\leq r \sum_i p_i D\left(|i\rangle\langle i| \otimes (\mathcal{F}_i(|k\rangle\langle k|)^{\otimes g}) \middle\| |i\rangle\langle i| \otimes \left(\frac{\mathbb{I}}{2^n}^{\otimes g}\right)\right) \\ &= rg \sum_i p_i D\left(\mathcal{F}_i(|k\rangle\langle k|) \| \frac{\mathbb{I}}{2^n}\right). \end{aligned} \tag{13}$$

It then suffices to upper-bound the expression (13). But by applying Markov's inequality to the probability distribution over the circuit ensemble used in our construction, Eq. (59) shows that there exist a set of circuits $\{\mathcal{F}_i\}$ on n qubits and depth d for which $D\left(\mathcal{F}_i(|k\rangle\langle k|) \| \frac{\mathbb{I}}{2^n}\right)$ is at most a constant factor times exponentially small in n, d, which, via the central lemma, yields the conclusion that the total number of circuit runs rg can go up to exponential in n, d.

A. Related work

We review what was known about theoretical limits of error mitigation prior to our work. Prior art typically falls into two camps: 'go' results, which propose new error mitigation algorithms and (often numerically) study their guarantees, and 'no-go' theorems, which demarcate the resource limitations of these algorithms. Before mentioning the more quantitative works on limitations of quantum error mitigation, it is worth noting the experimental motivation for such results: there is a body of heuristic evidence of instances of quantum error mitigation performing poorly in certain cases. For example, Ref. [12] discusses limitations of the extrapolation method to the noise-free case being unable to approach the mean of the error-free circuit. Ref. [13] has also shown that for *probabilistic error cancellation* assuming a sparse noise model, the sampling overhead scales exponentially in both the depth and the width of the circuit.

Concerning sample complexity bounds, Ref. [14] has presented a general framework. In terms of theoretical results, Refs. [14–16] also study the worst-case sample complexity of weak error mitigation under depolarizing noise from information-theoretic considerations, and they show that error mitigation incurs an $\exp(d)$ blowup in sample complexity. However, as explained in the main text, given existing circuit depths, their results are exponentially weaker than ours. They do not mention strong error mitigation. While we have made a case that our findings impose

strong limitations also on input state-*aware* protocols, their results do not apply to such protocols. For these papers implicitly assume knowledge of the noiseless circuit—and with knowledge of the input state on top of that, an error mitigation algorithm could produce expectation value estimates without taking even a single sample, simply by simulating the circuit on the input state, even if it takes an exponential amount of computation.

Ref. [16] consider two desiderata for the error mitigation algorithm: a metric, similar to our Eq. (43), that requires the algorithm to be probably-approximately correct on *each* observable (note that this metric is marginally weaker than Eq. (43), which requires the algorithm to succeed with $1-\delta$ probability at predicting expectation values for *all* observables) and the maximum bias/standard deviation of the estimators. While they also study the relative entropy decay to the maximally- mixed state, their ultimate sample complexity lower bound decays exponentially only in the *depth* D of the circuit—and polynomially in the number of qubits n. By contrast, our sample complexity lower bounds (Theorem 3) decay *exponentially* in both n and D—indicating that their bounds are exponentially loose. Moreover, we make an incursion into proving our results for non-unital noise models.

Refs. [15, 16] studies weak error mitigation from the perspective of Fisher information. More precisely, the authors show that the quantum Fisher information associated with layered noisy circuits decays exponentially with circuit depth, which thus also lower bounds the variance of any unbiased estimator of expectation values. However, we have our reservations about using estimator variance as a metric of resources required, as it gives a very loose bound on the sample complexity to attain a certain estimation error. Take, for instance, the random variable which is e^n with probability $e^{-n/2}$, $-e^n$ with probability $e^{-n/2}$ and 0 otherwise. This zero-mean random variable has exponential variance, but with a sub-exponential number of samples, the empirical mean is overwhelmingly likely to be exactly the true mean. Of course, such pathological examples use the fact that the underlying random variables take exponentially large values, but this is also the case the case for some error mitigation protocols.

II. CONCEPTUAL BACKGROUND AND CONTRIBUTIONS

We first show a basic version of our theorem bounding the sample complexity of error mitigation in a circuit subject to depolarizing noise. This theorem already has all the features of our main results but is weaker, in the sense that we will only be able to show an exponential-in-D dependence of the sample complexity, while our main result improves this to an exponential in both n, D.

A. Appetizer: mitigating depolarizing noise requires exponential-in-D samples

We are finally ready to present our 'appetizer' theorem. Let us first establish the following notation:

Definition 1 (Circuit with depolarizing noise)**.** *For any noiseless circuit $\mathcal{C}$ of depth D acting on n qubits, define the noisy circuit with depolarizing noise parameter p, $\Phi_{\mathcal{C},p}$, as*

$$\Phi_{\mathcal{C},p} \coloneqq \mathcal{C}^{(D)} \circ \mathcal{D}_p^{\otimes n} \cdots \circ \mathcal{C}^{(2)} \circ \mathcal{D}_p^{\otimes n} \circ \mathcal{C}^{(1)}, \tag{14}$$

where $\mathcal{C}^{(i)}$ represents the ith layer of the circuit $\mathcal{C}$, and $\mathcal{D}_p$ is a local, single-qubit depolarizing channel.

We will now present the key reduction we will use to bound the sample complexity of error mitigation. This reduction is visually represented in Fig. 2. To do so, let us step away from the error mitigation setting for a moment, and first consider the following problem of *state discrimination in the presence of noise*:

Problem 1 (State discrimination in the presence of noise)**.** *Fix a set $\{\rho_0, \rho_1, \ldots, \rho_N\}$ of $N+1$ quantum states and a circuit $\mathcal{C}$ acted upon by noise $\mathcal{N}$. Suppose that a distinguisher has knowledge of $\mathcal{C}$ and $\mathcal{N}$, and is given access to copies of the state $\Phi_{\mathcal{C},\mathcal{N}}(\rho_i)$, with the index $i \in \{0, 1, \ldots, N\}$ unknown. What is the fewest number of copies of $\Phi_{\mathcal{C},\mathcal{N}}(\rho_i)$ needed in order to identify i with high probability, in terms of the noise strength?*

The following variation of Fano's lemma can be used to address this question:

Lemma 2 (Corollary 2.6 of Ref. [1])**.** *Let $P_0, \ldots, P_N$ be probability measures on some state space X such that*

$$\frac{1}{N+1}\sum_{k=0}^{N} D(P_k \| P_0) \leq \alpha \log(N) \tag{15}$$

for $0 < \alpha < 1$. Then the minimum average probability of error over tests $\psi : X \to \{0, 1, \ldots, N\}$ that distinguish the probability distributions $P_0, \ldots, P_N$ which we define as

$$\bar{p}_{e,N} \coloneqq \inf_{\psi} \frac{1}{N+1} \sum_{j=0}^{N} P_j(\psi \neq j) \tag{16}$$

satisfies

$$\bar{p}_{e,N} \geq \frac{\log(N+1) - \log(2)}{\log(N)} - \alpha. \tag{17}$$

Let us explain what this lemma implies about the sample complexity of state discrimination in the presence of noise (Problem 1). Intuitively, dialing up the noise on the circuit $\mathcal{C}$ makes the various output states $\Phi_{\mathcal{C},\mathcal{N}}(\rho_i)$ less distinguishable from each other—and so, more copies of $\Phi_{\mathcal{C},\mathcal{N}}(\rho_i)$ must be taken to determine the true identity of the input state. This Lemma formalizes this intuition. For let $P_0, \ldots, P_N$ be distributions over the output of the m-copy distinguisher in Problem 1 when the true state is $\rho_0 \ldots \rho_N$, respectively. One can then show that, for $\mathcal{N}$ being depolarizing noise of parameter p on generic depth-D circuits, the average distance of $P_1, \ldots, P_N$ to P_0 (the left-hand-side of (15)) scales as $\alpha \log(N) = p^{2D} \log(N)$. To get a constant probability of error in (17), α must be constant. In order for α to be constant, one needs to choose m scaling as p^{-2D}.

We can go one step further and use Lemma 2 to bound the performance of error mitigation. The proof proceeds according to the following two basic steps:

1. Embed an error mitigation protocol into a solver for a state discrimination problem. This means, we identify a state discrimination problem such that an error mitigation algorithm can be transformed, with no additional copies required, into a distinguisher that solves the state discrimination problem.
2. Compute Fano's lower bound for the number of copies needed for the state discrimination problem.

More formally, we now state and prove the following theorem:

Theorem 1 (Appetizer: Error mitigation under depolarizing noise requires exponential-in-depth samples)**.** *Let $\mathcal{A}$ be an input state-agnostic weak error mitigation algorithm (see Definition 5) that takes as input m noisy copies $\Phi_{\mathcal{C},p}(\rho)$. Suppose that for any circuit $\mathcal{C}$ acted upon by depolarizing noise of parameter p, and any set of observables $\mathcal{M} = \{O_i\}_{i\in[n]}$, $\mathcal{A}$ is able to produce estimates $\{\hat{o}_i\}_{i\in[n]}$ such that with probability at least $1-\delta$,*

$$|\hat{o}_i - \mathrm{Tr}\left[O_i \mathcal{C}(\rho)\right]| \le \epsilon \qquad \text{for all } i \in [n]. \tag{18}$$

Then there exists an observable set $\mathcal{M}$ and a set of input states ρ such that, as long as $\epsilon < 1/2$, the number of noisy copies, m, needed by $\mathcal{A}$, is lower-bounded as

$$m = \Omega(p^{-2D}) \tag{19}$$

for any circuit $\mathcal{C}$ on n qubits of depth D.

After we prove this theorem, we remark on extensions of our proof to EM protocols with other types of input state access, including those protocols that are input state-aware.

Proof of Theorem 1. Let $\mathcal{A}$ be an input state-agnostic weak error mitigation algorithm and consider the observable set $\mathcal{M} = \{\mathcal{C}(Z_j)\}_{j=1}^{n}$ where Z_j is the Pauli Z acting on qubit j. For ease of notation, we will denote by $\hat{z}_j$ the estimate of $\mathrm{Tr}[\mathcal{C}(Z_j)\mathcal{C}(\rho_i)]$. We are going to identify a set S of states (which is also the set of states referenced in the Theorem), such that to solve Problem 1 when the unknown state is chosen from S, it suffices to run $\mathcal{A}$ on copies of the unknown state and then do simple post-processing.

a. Step 1: A noisy state identification problem that can be solved by weak error mitigation. Consider choosing the unknown state ρ_i, $i \in [2^n+1]$, in Problem 1 from the following $N+1 = 2^n+1$ options:

- $\rho_N = \mathbb{I}/2^n$, the maximally-mixed state. If this option is chosen, say we are in **Case 1**.
- $\{\rho_x := |x\rangle\langle x|\}_{x\in\{0,1\}^n}$. If one of these options is chosen, say we are in **Case 2**.

(We emphasize that the state discrimination algorithm must output i, the label of the state, rather than simply decide whether it is in Case 1 or 2.) We will now show that Problem 1 can be solved with access to an error mitigation algorithm $\mathcal{A}$. First suppose hypothetically that the error mitigation algorithm were *perfect*; i.e., when given m copies of $\Phi_{\mathcal{C},\mathcal{N}}(\rho_i)$, $\mathcal{A}$ outputs estimates

$$\hat{z}_j = \mathrm{Tr}[\mathcal{C}^\dagger(Z_j)\mathcal{C}(\rho_i)] = \mathrm{Tr}[Z_j\rho_i] \qquad \forall j \in [n] \tag{20}$$

that are *exact*. Later we will show that the analysis below goes through if we assume only approximate estimates. But consider that, in Case 1, where ρ_i is $\rho_N = \mathbb{I}/2^n$, the true expected value of the observables is

$$\mathrm{Tr}[Z_j\mathbb{I}/2^n] = 0 \qquad \forall j \in [n] \tag{21}$$

i.e., a perfect error mitigation algorithm should estimate $\hat{z}_j = 0$ for every observable.

On the other hand, in Case 2, when $\rho_i = \rho_x$ for some $x = (x_1, x_2, \ldots, x_n) \in \{0,1\}^n$, the true expected value of the observables is

$$\mathrm{Tr}[|x\rangle\langle x|Z_j] = 2x_j - 1 \qquad \forall j \in [n] \quad , \tag{22}$$

i.e., a perfect error mitigation algorithm would output the estimate $\hat{z}_j = 1$ if $x_j = 1$ and $\hat{z}_j = -1$ if $x_j = 0$. Hence, if the error mitigation algorithm were perfect, one could perfectly distinguish Case 1 and Case 2; furthermore in the latter case, concatenating the set $\{(1+\hat{z}_j)/2\}_{j=1}^{n}$ results in an n-bit string corresponding to $x = (x_1, x_2, \ldots, x_n)$, the label of the actual input state. A perfect error mitigation algorithm thus solves the state identification problem perfectly.

Now we argue that state identification can still be solved by an error mitigation algorithm that returns only *approximate* estimates. By assumption, with probability $1-\delta$, the estimates are accurate up to additive error ϵ. As long as $\epsilon < 1/2$, we find

$$|\hat{z}_j - \mathrm{Tr}[\rho_i Z_j]| \le 1/2 \qquad \forall j \in [n] \quad , \tag{23}$$

(c.f. Eq. (20)). The *true* values, of course, remain the same: Eq. (21) and Eq. (22) still hold in Case 1 and Case 2 respectively. In particular, let $\hat{z} = (\hat{z}_1, \hat{z}_2, \ldots, \hat{z}_n)$ be the estimates produced by $\mathcal{A}$ when the input state is $\rho_N = \mathbb{I}/2^n$ (Case 1) and let $\hat{z}_x = (\hat{z}_{1,x}, \hat{z}_{2,x}, \ldots, \hat{z}_{n,x})$ be the estimates produced by $\mathcal{A}$ when the input state is $\rho_x = |x\rangle\langle x|$ (Case 2). Then Eq. (23) says that with probability $1-\delta$,

$$-\frac{1}{2} < \hat{z}_i < \frac{1}{2} \;\forall i \quad \text{(Case 1)} \quad \text{and} \quad \left\{ \begin{array}{ll} \frac{1}{2} < \hat{z}_{j,x} < \frac{3}{2} & \text{if } x_j = 0 \\ -\frac{3}{2} < \hat{z}_{j,x} < -\frac{1}{2} & \text{if } x_j = 1 \end{array} \quad \text{(Case 2)} \right\}. \tag{24}$$

Now, let $\mathsf{EstInput}$ be the algorithm that takes the output of $\mathcal{A}$, which is the set of estimates $\{\hat{z}_j\}_{j\in[n]}$, and does the following:

1. If there exists $j \in [n]$ such that $\hat{z}_j \in (-1/2, 1/2)$, it outputs that $\hat{x} = N$, i.e., the unknown state is ρ_N.
2. Else, it computes the n-bit string $\hat{x} = (\hat{x}_1, \hat{x}_2, \ldots, \hat{x}_n) \in \{0,1\}^n$ as

$$\hat{x}_j = \left\{ \begin{array}{ll} 0 & \text{if } \hat{z}_j \geq 0 \\ 1 & \text{if } \hat{z}_j < 0 \end{array} \right\}, \tag{25}$$

and outputs that the unknown state is $\rho_{\hat{x}}$.

It is easy to see from Eq. (24) that this procedure correctly identifies the unknown state, and hence solves Problem 1 with success probability $1-\delta$.

b. Step 2: Computing Fano's lower bound for state identification. The string $\hat{x}$ output by the above procedure is probabilistic. In particular, let $P_i(\hat{x})$ for $i \in [2^n+1]$ be the probability that running $\mathsf{EstInput} \circ \mathcal{A}$ on the unknown state ρ_i will output $\hat{x}$. Since the last step of our procedure is to sample from P_i and then report the output, this last step is a single-sample test to distinguish probability distributions. By Fano's Lemma (Lemma 2), any such test must fail with probability no less than

$$\frac{\log(N+1) - \log(2)}{\log(N)} - \alpha \xrightarrow{N \text{ large}} 1 - \alpha \tag{26}$$

where $\alpha \log(N)$ is defined to be an *upper bound* to the quantity

$$\frac{1}{N+1} \sum_{k=0}^{N} D(P_k \| P_N) \quad . \tag{27}$$

We will show later (in Proposition 1) that we can bound the average relative entropy between P_k and P_N as follows:

$$\frac{1}{N+1} \sum_{k=0}^{N} D(P_k \| P_N) \leq p^{2D} m \log(N), \tag{28}$$

so it suffices to set $\alpha = p^{2D} m$. Therefore, in order for the test to have a constant failure probability δ, it must take at least $m = p^{-2D}(1-\delta)$-many samples. □

Eq. (27) is a critical quantity in Fano's Lemma and our argument, so let us interpret it: take the maximally-mixed state $\mathbb{I}/2^n$ to be the reference state and the probability distribution output by our noisy state discrimination algorithm on $\mathbb{I}/2^n$ to be the reference probability distribution P_N. Eq. (27) examines the divergence between the output of the algorithm initialized with one of the states ρ_x, and that when initialized on the reference state. Intuitively, if this quantity is high, it indicates that the states are "far apart", and hence the distinguishing algorithm could potentially have a lower probability of error.

We remark that in our construction we have used the error mitigation protocol on the observables $C(Z_i)$. These observables will in general be supported on almost all qubits and we can efficiently compute their expectation value for all quantum circuits. So the reader might argue that these expectation values are unlikely to be the target of error mitigation protocols and they are usually designed with local observables in mind. However, note that the only property of the observables we have used in the proof was that they allow us to distinguish different input states from the maximally mixed state. Thus, for any set of observables whose expectation values differ for outputs of the noiseless circuit and the maximally mixed state, the same proof goes through. This is because the noiseless expectation values can be used to envision a statistical test to distinguish the output states.

Indeed, bounding the distinguishability of the states in our set (which becomes the left-hand-side of Eq. (27) after one application of data-processing) is the technical crux of our argument. We present a basic bound in Proposition 1 and we will gradually refine this bound in the next few sections to get progressively more severe dependencies of the sample complexity of error mitigation on various parameters of the problem. In this Proposition, we will suppress the notation for the classical inputs $\mathcal{C}, p, \mathcal{M}$ to the error mitigation algorithm $\mathcal{A}$ (see Def. 4) and use $\mathcal{A}(\Phi_{\mathcal{C},p}(\rho)^{\otimes m})$ to denote $\mathcal{A}$ with all the above classical inputs, and a quantum input which is m copies of the quantum state $\Phi_{\mathcal{C},p}(\rho)$.

Proposition 1 (Simple bound on average divergence). *Let P_k, $k \in \{0,1\}^n$ be the probability distributions over the outputs of the algorithm for noisy state discrimination described in the proof of Theorem 1, run on the input state ρ_k. That is, P_k for $k \in \{0,1\}^n$ is the probability distribution output by*

$$\mathsf{EstInput} \circ \mathcal{A}(\Phi_{\mathcal{C},p}(\rho_k)^{\otimes m}) \quad , \tag{29}$$

and similarly let P_N where $N = 2^n$ be the probability distribution over the output of

$$\mathsf{EstInput} \circ \mathcal{A}(\Phi_{\mathcal{C},p}(\rho_N)^{\otimes m}) \quad . \tag{30}$$

Then it holds that

$$\frac{1}{N+1}\sum_{k=0}^{N} D(P_k\|P_N) \leq p^{2D} m\log(N). \tag{31}$$

Proof. Firstly, note that since $\rho_N := (\mathbb{I}/2^n)^{\otimes m}$, we can explicitly write down the output of the noisy circuit on this state as $\Phi_{\mathcal{C},p}(\rho_N) = (\mathbb{I}/2^n)^{\otimes m}$, as the maximally-mixed state is the fixed point of depolarizing noise. Since $P_k = \mathsf{EstInput}\circ\mathcal{A}(\Phi_{\mathcal{C},p}(\rho_k)^{\otimes m})$ and $P_N = \mathsf{EstInput}\circ\mathcal{A}((\mathbb{I}/2^n)^{\otimes m})$, by the data-processing inequality we have

$$D(P_k\|P_N) \leq D(\Phi_{\mathcal{C},p}(\rho_k)^{\otimes m}\|(\mathbb{I}/2^n)^{\otimes m}), \tag{32}$$

and so it suffices to bound the quantity $D(\Phi_{\mathcal{C},p}(\rho_k)^{\otimes m}\|(\mathbb{I}/2^n)^{\otimes m})$. The rest of this proof shows that for $k\in\{0,1\}^n$:

$$D(\Phi_{\mathcal{C},p}(\rho_k)^{\otimes m}\|(\mathbb{I}/2^n)^{\otimes m}) \leq p^{2D} nm \tag{33}$$

by the unitary invariance of the relative entropy, the strong data-processing inequality for depolarizing noise and the additivity of the relative entropy. To see this, observe that

$$\begin{aligned} D(\Phi_{\mathcal{C},p}(\rho_k)^{\otimes m}\|(\mathbb{I}/2^n)^{\otimes m}) &= mD(\Phi_{\mathcal{C},p}(\rho_k)\|\mathbb{I}/2^n) && (34)\\ &\leq m\,p^{2D} D(\rho_k\|\mathbb{I}/2^n)\\ &= m\,p^{2D} D(|k\rangle\langle k|\|\mathbb{I}/2^n)\\ &= p^{2D} mn, && (35)\end{aligned}$$

where the first equality follows from the additivity of relative entropy, the first inequality is strong data-processing for D layers of depolarizing noise with parameter p [17],

$$D(\Phi_{\mathcal{C},p}(\rho_k)\|\mathbb{I}/2^n) \leq p^{2D} D(\rho_k\|\mathbb{I}/2^n), \tag{36}$$

and the third equality follows from direct calculation. Lastly, we clearly have $D(P_N\|P_N) = 0 < p^{2D}nm$. Therefore,

$$\frac{1}{N+1}\sum_{k=0}^{N} D(P_k\|P_N) < p^{2D} nm \tag{37}$$

as desired. □

Though it follows immediately, we emphasize that inequality (32) still holds in the case that the error mitigation algorithm is not *entirely* classical, but performs some (usually small amount of) quantum post-processing on the noisy states – as is the case with virtual distillation [2], where a single layer of two-qubit gates is applied on the noisy states. Thus, all our conclusions still hold in this setting.

Theorem 1 states that a general input state-*agnostic* error mitigation protocol should have a worst-case sample complexity scaling exponentially in D. So far, our proof method does *not* encompass input state-*aware* error mitigation protocols, which are those that, in addition to knowing the noiseless circuit, also know its input state. Such knowledge would be an unauthorized 'back door' for the error mitigation algorithm used inside the solver for noisy state discrimination.

Can we remedy this lack? We offer two affirmative answers. The first follows immediately from close inspection of our proof method: our results extend to even error mitigation protocols working with a guarantee that the input state is chosen from a known set of size $N+1$—a situation somewhere between input-state agnosticism and full input-state awareness. Secondly, we will next show that a modification of our argument allows us to make a statement about the complexity of fully input-state *aware* error mitigation algorithms.

B. The input-state *aware* case

The input-state *aware* setting refers to settings where, in doing the translation from the real-life setting to the mathematical framework we have defined in Problem 1, the error mitigation protocol does not return the correct results if the input state to the noisy circuit is chosen arbitrarily. This could be because the error mitigation algorithm is designed with a particular, fixed circuit input state 'hard-coded' into the computation that is done on the noisy output states of the circuit. Clifford data regression in I is one example of such a protocol.

Without further assumptions, we cannot expect lower bounds on the sample complexity of the general error mitigation task in the input-state aware setting, because there always exists a trivial error mitigation algorithm that does not access the quantum device—hence uses zero samples—and instead simulates the quantum circuit to obtain the desired properties of the noiseless state. This would not be possible in the setting of the previous subsection, because there the error mitigation algorithm is restricted to not use information about the input state to the circuit. While we previously found sample complexity bounds on *any* input state-agnostic error mitigation algorithm outputting expectation value estimates, we now change our perspective: we ask how many samples would be needed by an error mitigation algorithm whose outputs *are meaningfully different* from one that obtains comparable results through purely classical means. In other words, if an error mitigation algorithm is successful but it invokes the quantum device an insufficient number of times, then there exists an equivalent classical algorithm. A tool we will use is the following bound on two-hypothesis testing due to Le Cam:

Lemma 3 (Two-hypothesis testing [1], Theorem 2.2iii))**.** *For two probability distributions P_0, P_1 satisfying*

$$\|P_0 - P_1\|_{TV} \leq \alpha \quad , \tag{38}$$

the minimum probability of error of any test that draws a single sample and distinguishes P_0 and P_1 is

$$P_{error} \geq \frac{1-\alpha}{2} \quad . \tag{39}$$

Our results on the convergence of random quantum circuits to the maximally mixed state can easily be used to obtain bounds in the total variation distance by an application of Pinker's inequality and the data processing inequality. In the following theorem, we prove that our construction can be used to make a statement about the sample complexity of input state aware error mitigation under depolarizing noise, as long as one demands that such error mitigation makes meaningful use of the quantum inputs. We then remark on how our technique can be extended even to the case of non-unital noise.

Theorem 2 (Input state-awareness does not break the curse of exponentiality)**.** *Let $\mathcal{A}$ be an input state-*aware *weak error mitigation algorithm that takes:*

- Quantum *input: At most m copies of the noisy output state $\Phi_{\mathcal{C},\mathcal{N}}(\rho)$.*
- Classical *input, $CL(\mathcal{C},\mathcal{N},\rho)$: Classical descriptions of $\mathcal{C}$, the noise channel $\mathcal{N}$ acting on $\mathcal{C}$ and* the input state to $\mathcal{C}$*, which we call ρ,*

and outputs a distribution over possible outcomes. Then either:

- ***Case I:** For some $c > 1$, $mc^{-\tilde{\mathcal{O}}(nD)} = \Omega(1)$, i.e., even for input-state aware error mitigation algorithms, exponential in n, D samples are needed; or*
- ***Case II:** no algorithm exists can distinguish between the outputs of $\mathcal{A}(\Phi_{\mathcal{C},\mathcal{N}}(\rho)^{\otimes m}, CL(\mathcal{C},\mathcal{N},\rho))$ and $\mathcal{A}((\mathbb{I}/2)^{\otimes m}, CL(\mathcal{C},\mathcal{N},\rho))$ with an error probability significantly bounded away from $2/3$.*

Proof. Suppose we are not in Case I. This implies that $mc^{-\tilde{\mathcal{O}}(nD)} = \Theta(1)$. In Sections III and IV we proved there exists a circuit $\mathcal{C}^*$ such that for any algorithm $\mathcal{A}$ run on either m copies of the state $\Phi_{\mathcal{C}^*,\mathcal{N}}(\rho)$ or m copies of the maximally-mixed state,

$$\begin{aligned} &D(\mathcal{A}(\Phi_{\mathcal{C}^*,\mathcal{N}}(\rho)^{\otimes m}, CL(\mathcal{C}^*,\mathcal{N},\rho))\|\mathcal{A}(\mathbb{I}/2^{n\otimes m}, CL(\mathcal{C}^*,\mathcal{N},\rho))) \\ &\leq mD(\Phi_{\mathcal{C}^*,\mathcal{N}}(\rho)\|\mathbb{I}/2^n) \leq mc^{-\tilde{\mathcal{O}}(nD)} = \Theta(1) \end{aligned} \tag{40}$$

therefore, Lemma 3 implies that the minimum probability of error distinguishing the output of $\mathcal{A}(\Phi_{\mathcal{C}^*,\mathcal{N}}(\rho)^{\otimes m}, CL(\mathcal{C},\mathcal{N},\rho))$ from the output of $\mathcal{A}((\mathbb{I}/2^n)^{\otimes m}, CL(\mathcal{C},\mathcal{N},\rho))$ is $1/2 - o(1)$, proving the claim. □

The statement above implies that if the error mitigation was successful, then we could have supplied the algorithm with copies of the maximally mixed state as an input and observed similar outcomes.

We can prove a similar statement in the case of non-unital noise. However, unitary circuits affected by product non-unital noise are qualitatively different: whereas in the case of unital circuits the outputs will converge to the maximally mixed state asymptotically, non-unital random circuits do not converge to a single state. For instance, as we will show later in the setting of our toy example of noise being applied after 2−design, what happens instead is that after we apply D layers of 2−designs followed by noise, yielding the evolution T_D, we have

$$\mathbb{E}\left[\|T_D(\rho_i - \rho_j)\|_1\right] \leq 2^{\frac{n+1}{2}} q_n^{\frac{D}{2}}, \tag{41}$$

where $q_n = \mathcal{O}(c_1^n)$ for $c_1 < 1$ is an exponentially small in n parameter that depends on the noise channel and ρ_i, ρ_j orthogonal inputs. That is, any two inputs will be mapped to essentially the same state σ_D after D applications of the circuit. However, it is in general not possible to compute this state efficiently to simulate it classically and unless the noise is unital the sequence $\sigma_1, \sigma_2, \ldots$ does not necessarily converge. Thus, in the argument we have used above, the maximally mixed state would have to be replaced by this state σ_D. Thus, the same conclusions would hold, but the output of the error mitigation algorithm would be indistinguishable from the output given as input copies of a fixed state σ_D that is independent of the input ρ. We cannot exclude the possibility that sampling from σ_D gives some extra computational power, but as in general there is no way to compute σ_D, it is unclear how to profit from it. In summary, the output of the error mitigation algorithm given the samples from the noisy device would be indistinguishable from that of the same error mitigation algorithm given as input copies of a state σ_D that is independent of the input.

As an interlude, let us review the conceptual contribution of this section and look ahead to the next. Theorem 1 leveraged a relation between a noisy state distinguishability problem and a related error mitigation problem to show that the worst-case sample complexity of error mitigation needs to depend exponentially on the depth of the circuit whose errors are being mitigated. The proof of this theorem depended crucially on upper-bounding the quantity

$$\frac{1}{N+1}\sum_{k=0}^{N} D(\Phi_{\mathcal{C},p}(\rho_k)\|\Phi_{\mathcal{C},p}(\mathbb{I}/2^n)) \quad , \tag{42}$$

which is a sort of average distance between the output of the noisy circuit (whose errors we would like to mitigate) and its fixed point, which is the maximally-mixed state. This quantity controls the sample complexity of noisy state discrimination via Fano's Lemma, and in the chain of inequalities (34) to (35) of the proof of Proposition 1, we upper-bounded it by $\alpha \log N$ where we computed α in terms of m and D. Plugging into Fano's Lemma then completed the argument and yielded the lower bound on sample complexity m.

In the next section, we will refine the upper-bound on (42), so that α has an exponential dependence not only on D, but also on n. Plugging this into Fano's Lemma yields, in turn, a worst-case sample complexity m that depends exponentially on *both* of these quantities. We will achieve this improvement by carefully constructing the input states $\mathcal{C}^{-1}(|x\rangle\langle x|)$. In fact, the key will be a perspicacious choice of $\mathcal{C}$, which leads to a worst-case-bound *better* than the bound on the right-hand-side of Eq. (36), which has been proven for general $\mathcal{C}$.

III. RAPIDLY MIXING CIRCUITS ARE HARD TO ERROR MITIGATE

In this section, we show that there exists a 'rapidly mixing' quantum circuit $\mathcal{C}^*$. In so doing we prove a stronger version of our results, which we state in Theorem 4. This circuit is rapidly mixing in the sense that, its output states

$$\Phi_{\mathcal{C}^*,p}(\mathbb{I}/2^n \otimes |0\rangle\langle 0|^{\otimes s}), \Phi_{\mathcal{C}^*,p}(\rho_k \otimes |0\rangle\langle 0|^{\otimes s}), \quad k \in \{0,1\}^n, \tag{43}$$

for the input states $\{\rho_k\}$ and the maximally mixed state (concatenated with auxiliary qubits), satisfies

$$\frac{1}{N+1}\sum_{k=0}^{N} D(\Phi_{\mathcal{C}^*,p}(\rho_k \otimes |0\rangle\langle 0|^{\otimes s}) \| \Phi_{\mathcal{C}^*,p}(\mathbb{I}/2^n \otimes |0\rangle\langle 0|^{\otimes s})) < c^{\Omega(nD)}, \tag{44}$$

for some constant $c < 1$ (c.f. Eq. (42)). Here the notation $\Phi_{\mathcal{C}^*,p}$ refers to a noisy version of the circuit $\mathcal{C}$.

Let us compare this to what we obtained in the previous section. In Eq. (37), for a generic $\mathcal{C}$, we could only prove that the average distinguishability could be bounded by $p^{\Omega(D)}nm$, which does not have an n dependence in the exponent. Thus when $D = O(\log n)$, the old bound is exponentially weaker in n. After we prove the improved bound for our particular circuit $\mathcal{C}^*$, we will conclude by again plugging this bound into the information-theoretic outer loop of our argument, which uses the sample complexity of noisy state distinguishability to lower bound that of error mitigation.

A. Technical background

In fact, we do not construct $\mathcal{C}^*$ explicitly. Instead, we let $\mathcal{C}^*$ be a concatenation of blocks, i.e.,

$$\mathcal{C}^* = \widetilde{\mathcal{C}}_\ell \circ \cdots \circ \widetilde{\mathcal{C}}_2 \circ \widetilde{\mathcal{C}}_1, \tag{45}$$

where every *block* is a circuit $\widetilde{\mathcal{C}}_j$ sampled independently from an *ensemble* of circuits (described in Lemma 4). We will in fact show that the noisy version of $\mathcal{C}^*$, when run on the input states $\mathbb{I}/2^n$ and ρ_k, $k \in \{0,1\}^n$, on expectation displays the scaling we claim in Eq. (44). We can then use the probabilistic method to conclude that there *exists* one particular circuit that achieves the same bound. Every block in $\mathcal{C}^*$ is constructed via the following prescription by Cleve, Leung, Liu and Wang [18]:

Lemma 4 (Exact unitary 2-designs from Clifford gates [18])**.** *With circuits $\widetilde{\mathcal{C}}$ consisting of $O(n\ \log^2\ n \log\log n)$ single- and two-qubit Clifford gates and $\tilde{O}(n)$ auxiliary qubits, in $O(\log^2(n))$ depth, it is possible to implement an* exact *unitary 2-design on n qubits. The auxiliary qubits all start in the state $|0\rangle\langle 0|$ and are returned to this state at the end of the circuit.*

In other words, the above is a prescription for constructing Clifford circuits $\widetilde{\mathcal{C}}$ that form unitary 2 designs on a subset of the qubits. Each such circuit has the property that

$$\widetilde{\mathcal{C}}(\rho \otimes |0\rangle\langle 0|^{\otimes s}) = \mathcal{C}(\rho) \otimes |0\rangle\langle 0|^{\otimes s}, \tag{46}$$

where s is the number of auxiliary qubits and $\mathcal{C}$ is a unitary sampled from an exact unitary 2-design.

B. Dealing with noise within the circuits

In this subsection, we wish to consider a noisy version of the circuit $\mathcal{C}^*$ defined in Eq. (45). This means that in between every layer of single- and two-qubit Clifford gates comprising each circuit $\widetilde{\mathcal{C}}_j$, there is a layer of depolarizing noise on every qubit. In particular, the noisy version of the block $\widetilde{\mathcal{C}}_j$ is of the form

$$\Phi_{\widetilde{\mathcal{C}}_j,\vec{p}_j} = \mathcal{D}_{p_{D'}}^{\otimes(n+s)} \circ \widetilde{\mathcal{C}}_j^{(D')} \circ \cdots \circ \mathcal{D}_{p_2}^{\otimes(n+s)} \circ \widetilde{\mathcal{C}}_j^{(2)} \circ \mathcal{D}_{p_1}^{\otimes(n+s)} \circ \widetilde{\mathcal{C}}_j^{(1)}, \tag{47}$$

where $\vec{p}_j = (p_1, p_2, \ldots, p_{D'})$ is a vector of single-qubit depolarizing noise parameters and D' is the depth of the construction of Ref. [18], as specified in Lemma 4 above. The superscript indexes the layer of the circuit and the subscript indexes the block. The noisy version of the overall circuit $\mathcal{C}^*$, which is the subject of our analysis, is then

$$\Phi_{\mathcal{C}^*,\vec{p}} := \Phi_{\widetilde{\mathcal{C}}_\ell,\vec{p}_\ell} \circ \cdots \circ \Phi_{\widetilde{\mathcal{C}}_2,\vec{p}_2} \circ \Phi_{\widetilde{\mathcal{C}}_1,\vec{p}_1}. \tag{48}$$

We now show that we can simplify the form of this noisy circuit by making use of an important property of Clifford gates, which can even be taken to be their defining property, which is that they map Pauli operators to other Pauli, up to a sign. We state this useful property more formally in Lemma 5, which leads to the simplified form of the circuit presented in Lemma 6 and illustrated in Figure 1.

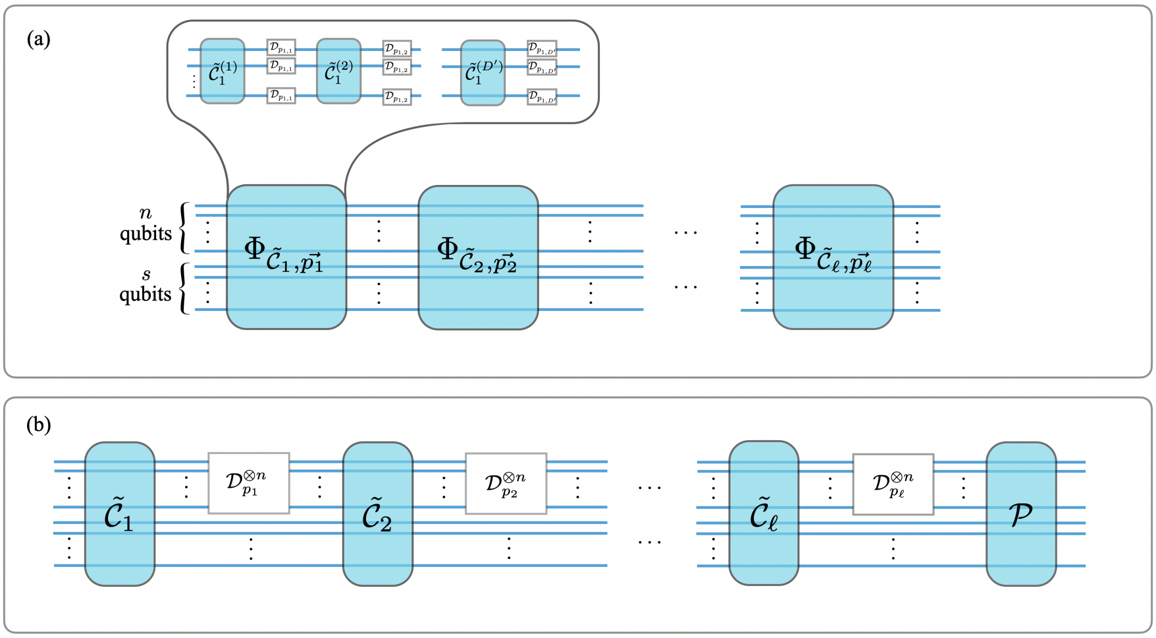

FIG. 1. A sketch of the constructions used in this subsection. Figure a) depicts the noisy circuit that results from our prescription of iterating the construction in Ref. [18] ℓ times (refer to Eq. (47) and (48)) and using our model of local depolarizing noise. Figure b) depicts how we can re-write the circuit in Figure a) by pushing the depolarizing noise on the auxiliary qubits to the end of the circuit, as stated in Lemma 6.

Lemma 5 (Pushing a Clifford past a Pauli channel)**.** *Let $\mathcal{P}$ be a Pauli channel and let $\mathcal{C}$ be a Clifford unitary. Then there exists a Pauli channel $\mathcal{P}'$ such that*

$$\mathcal{C} \circ \mathcal{P} = \mathcal{P}' \circ \mathcal{C}. \tag{49}$$

Proof. Let $\mathcal{P}' = \mathcal{C} \circ \mathcal{P} \circ \mathcal{C}^\dagger$. It is clear that Eq. (49) holds. Furthermore, we see that letting $\mathcal{P} = \sum_{P \in \{I,X,Y,Z\}^{\otimes n}} q_P P(\cdot)P$, then

$$\mathcal{P}'(\rho) = \sum_{P \in \{I,X,Y,Z\}^{\otimes n}} q_P C P C^\dagger (\cdot) C^\dagger P C.$$

As C is a Clifford unitary, $CPC^\dagger$ will also be a Pauli string, up to a sign. Thus, we conclude that the Kraus operators of $\mathcal{P}'$ are also Paulis and it is a Pauli channel.

□

Invoking this property (and noting that depolarizing channels are Pauli channels), we may rewrite $\Phi_{\mathcal{C}^*,\vec{p}}$ in a way that is more convenient to us: namely we rewrite the circuit so that part of the noise has been pushed to the end of the circuit. Note that we retain the noise on the system.

Lemma 6 (Pushing Paulis to the end of the circuit)**.** *Consider the noisy circuit on $n+s$ qubits defined in Eq. (48). There exists a Pauli channel $\mathcal{P}_{n+s}$, acting on all $n+s$ qubits, such that the circuit*

$$\mathcal{P}_{n+s} \circ (\mathcal{D}_{p_\ell}^{\otimes n} \otimes \mathrm{id}_s) \circ \tilde{\mathcal{C}}_\ell \circ \cdots \circ (\mathcal{D}_{p_2}^{\otimes n} \otimes \mathrm{id}_s) \circ \tilde{\mathcal{C}}_2 \circ (\mathcal{D}_{p_1}^{\otimes n} \otimes \mathrm{id}_s) \circ \tilde{\mathcal{C}}_1 \tag{50}$$

is equivalent to $\Phi_{\mathcal{C}^*,\vec{p}}$. In other words, all of the noise acting on the s auxiliary qubits and within the circuit for each design can be pushed to the end, and the intermediate 2-designs $\tilde{\mathcal{C}}_i$ can be treated as noiseless.

Proof. First, note that all gates of the circuit are Clifford and all the noisy channels are assumed to be product of $1-$ qubit Pauli noise channels. As observed in Lemma 5, given a Pauli channel followed by a Clifford gate, we can always obtain the same transformation by first applying the gate followed by a (typically different) Pauli channel. Furthermore, Pauli channels all commute with each other. To see this, note that Pauli strings are the eigenvectors of all Pauli channels. As they share a common eigenbasis, they all commute.

Thus, we can take all the individual depolarizing channels either acting on the auxiliary systems or within the blocks that generate each design and commute them to end of the circuit, leaving only the depolarizing channels acting *between* the designs (see Fig. 1b). This gives the desired representation. □

We briefly remark on how the above calculations will be used in our proof. We will again use the central lemma, Lemma 1, but now with the noisy circuits we have specially constructed: $\mathcal{C}' = \Phi_{\mathcal{C}^*,\vec{p}}$. That is, consider a new instance of the noisy state distinguishability problem

(Problem 1), in which the task is to identify the label k given a noisy state from the set

$$\Phi_{\mathcal{C}^*,\vec{p}}(|k\rangle\langle k|\otimes|0\rangle\langle 0|^{\otimes s}),\quad k\in\{0,1\}^n,\quad \text{ and } \Phi_{\mathcal{C}^*,\vec{p}}(\mathbb{I}_n/2^n\otimes|0\rangle\langle 0|^{\otimes s}). \tag{51}$$

Let us denote $\rho_k := |k\rangle\langle k|\otimes|0\rangle\langle 0|^{\otimes s}$. We then want to consider the quantity

$$D(\Phi_{\mathcal{C}^*,\vec{p}}(\rho_k)\|\Phi_{\mathcal{C}^*,\vec{p}}(\mathbb{I}_n/2^n\otimes|0\rangle\langle 0|^{\otimes s})). \tag{52}$$

Lemma 6 will simplify the task of bounding this quantity. Recall the result in (50). In particular, let

$$\widetilde{\mathcal{N}} := (\mathcal{D}_{p_\ell}^{\otimes n}\otimes \mathrm{id}_s)\circ\widetilde{\mathcal{C}}_\ell\circ\cdots\circ(\mathcal{D}_{p_2}^{\otimes n}\otimes \mathrm{id}_s)\circ\widetilde{\mathcal{C}}_2\circ(\mathcal{D}_{p_1}^{\otimes n}\otimes \mathrm{id}_s)\circ\widetilde{\mathcal{C}}_1, \tag{53}$$

so that $\Phi_{\mathcal{C}^*,\vec{p}} = \mathcal{P}_{n+s}\circ\widetilde{\mathcal{N}}$. This re-writing is useful because recursive application of (46) implies that the state $\mathbb{I}_n/2^n\otimes|0\rangle\langle 0|^{\otimes s}$ is a fixed-point of $\widetilde{\mathcal{N}}$, a fact that we use in Eq. (55) below. From the data-processing inequality, we obtain

$$\begin{aligned}
&D(\Phi_{\mathcal{C}^*,\vec{p}}(\rho_k)\|\Phi_{\mathcal{C}^*,\vec{p}}(\mathbb{I}_n/2^n\otimes|0\rangle\langle 0|^{\otimes s})) && (54)\\
&= D((\mathcal{P}_{n+s}\circ\widetilde{\mathcal{N}})(\rho_k)\|(\mathcal{P}_{n+s}\circ\widetilde{\mathcal{N}})(\mathbb{I}_n/2^n\otimes|0\rangle\langle 0|^{\otimes s})) && (55)\\
&\leq D(\widetilde{\mathcal{N}}(\rho_k)\|\mathbb{I}_n/2^n\otimes|0\rangle\langle 0|^{\otimes s})\\
&\leq D\left(\left(\bigcirc_{j=1}^{\ell}\mathcal{D}_{p_j}^{\otimes n}\circ\mathcal{C}_j\right)(|k\rangle\langle k|)\otimes|0\rangle\langle 0|^{\otimes s}\|\widetilde{\mathcal{N}}(\mathbb{I}_n/2^n\otimes|0\rangle\langle 0|^{\otimes s})\right)\\
&= D\left(\left(\bigcirc_{j=1}^{\ell}\mathcal{D}_{p_j}^{\otimes n}\circ\mathcal{C}_j\right)(|k\rangle\langle k|)\|\mathbb{I}_n/2^n\right),
\end{aligned}$$

where we note that the channel $\bigcirc_{j=1}^{\ell}\mathcal{D}_{p_j}^{\otimes n}\circ\mathcal{C}_j$ acts only on the n system qubits and not on the auxiliary ones. We have thus reduced our task to upper-bounding the quantity

$$D\left(\left(\bigcirc_{j=1}^{\ell}\mathcal{D}_{p_j}^{\otimes n}\circ\mathcal{C}_j\right)(|k\rangle\langle k|)\|\mathbb{I}_n/2^n\right) \tag{56}$$

where the circuits $\mathcal{C}_j$ are noiseless and independently drawn from a unitary 2-design. Since the uniform ensemble of Clifford unitaries forms a unitary 2-design, we assume from now on that the $\mathcal{C}_j$ are Clifford unitary channels and proceed to bound Eq. (56) in the remainder of this section.

C. Bound for multiple layers of depolarizing noise

Let us recall that our goal is to bound the *average distinguishability* quantity

$$\frac{1}{N+1}\sum_{k=0}^{N} D(P_k\|P_N). \tag{57}$$

It turns out that we can obtain a k-independent bound on $D(P_k\|P_N)$; to do so, we invoke the following consequence of Lemma 1,which says that we can bound the expected relative entropy for each k by calculating the expected purity

$$\begin{aligned}
&\mathbb{E}_{C\sim\mathcal{E}}\left[D\left(\left(\bigcirc_{j=1}^{\ell}\mathcal{D}_{p_j}^{\otimes n}\circ\mathcal{C}_j\right)(|k\rangle\langle k|)\middle\|\frac{\mathbb{I}}{2^n}\right)\right]\leq n+\mathbb{E}_{C\sim\mathcal{E}}\left[\log\mathrm{Tr}\left[\left(\bigcirc_{j=1}^{\ell}\mathcal{D}_{p_j}^{\otimes n}\circ\mathcal{C}_j\right)(|k\rangle\langle k|)\right)^2\right]\right]\leq && (58)\\
&n+\log\mathbb{E}_{C\sim\mathcal{E}}\,\mathrm{Tr}\left[\left(\bigcirc_{j=1}^{\ell}\mathcal{D}_{p_j}^{\otimes n}\circ\mathcal{C}_j\right)(|k\rangle\langle k|))^2\right],
\end{aligned}$$

where $k\in\{0,1\}^n$ and the last step follows from Jensen's inequality. Let us now compute that purity. In Section IV we will compute the expected purity of the outcome of l applications of a channel with a $2-$design for arbitrary channels. In particular, in Prop. 4 we show that

$$\mathbb{E}_{C\sim\mathcal{E}}\,\mathrm{Tr}\left[\left(\bigcirc_{j=1}^{\ell}\mathcal{D}_{p_j}^{\otimes n}\circ\mathcal{C}_j\right)(|k\rangle\langle k|))^2\right]=\left(q_n^{\ell}(1-2^{-n})+2^{-n}\right), \tag{59}$$

where for a qubit depolarizing channel with depolarizing probability p we have

$$q_n=\frac{(3p^2+1)^n-1}{4^n-1}. \tag{60}$$

Inserting this bound into Eq. (58), we conclude that

$$\begin{aligned}
&\mathbb{E}_{C\sim\mathcal{E}}\left[D\left(\left(\bigcirc_{j=1}^{\ell}\mathcal{D}_{p_j}^{\otimes n}\circ\mathcal{C}_j\right)(|k\rangle\langle k|)\middle\|\frac{\mathbb{I}}{2^n}\right)\right] && (61)\\
&\leq n+\log(q_n^{\ell}(1-2^{-n})+2^{-n})=\log(2^n q_n^{\ell}+1)\leq 2^n q_n^{\ell}.
\end{aligned}$$

In particular, if

$$\ell > \frac{1}{2 - \log_2(4 - 3p(2-p))}, \tag{62}$$

then

$$2^n q_n^\ell \le c^n \tag{63}$$

for a constant $c < 1$.

D. Putting it all together

We finally feed the results of the previous subsections into the information-theoretic outer loop already used in Section II, and obtain a tighter bound on sample complexity. For conciseness, we state Theorem 3 for depolarizing noise that acts with the same strength after every circuit layer; however, as demonstrated in Section III C, the theorem holds with minor modifications for the case when the noise parameter differs for every layer.

Theorem 3 (Error mitigation with depolarizing noise requires exponential-in-n, D samples)**.** *Let $\mathcal{A}$ be an input state-agnostic weak error mitigation algorithm (see Definition 5) that takes as input m noisy copies of the output state of a circuit $\mathcal{C}$ acted upon by local depolarizing noise of parameter p. Suppose that for any circuit $\mathcal{C}$ acting on n qubits of depth D, where*

$$D = \Omega\left(\frac{\log^2(n)}{\log\left(\frac{4}{1+3p^2}\right)}\right), \tag{64}$$

and the set of observables $\mathcal{M} = \{Z_i\}_{i=1}^n$, $\mathcal{A}(\mathcal{C}, \mathcal{M}, p)$ is able to produce estimates $\{\hat{z}_i\}_{i\in[n]}$ such that with probability at least $1-\delta$,

$$|\hat{z}_i - \mathrm{Tr}\left[Z_i \mathcal{C}(\rho)\right]| \le \epsilon \qquad \textit{for all } i \in [n]. \tag{65}$$

Then as long as $\epsilon < 1/2$, there exists a constant $c \ge 1$, such that the number of noisy copies, m, needed by $\mathcal{A}(\mathcal{C}, \mathcal{M}, p)$, is lower-bounded as

$$m = c^{-\tilde{\mathcal{O}}(nD)} \quad , \tag{66}$$

where the notation $\tilde{\mathcal{O}}(.)$ hides polylogarithmic factors in n.

Proof. Let us first note that we must re-scale n by a constant factor because it now refers to the size of both system and auxiliary qubits (the constant factor is because only $s = \tilde{\mathcal{O}}(n)$ auxiliary qubits are required, according to Ref. [18]), while our bounds from the previous subsections were in terms of only the number of system qubits. From Eq. (61) and the linearity of expectation we conclude that

$$\mathbb{E}_{\mathcal{C}}\left[\frac{1}{N+1}\sum_{k=0}^{N} D\left(\mathcal{C}(|k\rangle\langle k| \otimes |0\rangle\langle 0|) \,\middle\|\, \sum_E p(E)\frac{\mathbb{I}}{2^n} \otimes \sigma_E\right)\right] \le c^{\Omega(n\ell)}) \quad . \tag{67}$$

Here, the expectation is over the distribution induced by constructing a circuit $\mathcal{C}$ by sequentially performing the randomized circuit construction of Ref. [18],

$$\ell \ge \frac{1}{\log\left(\frac{4}{1+3p^2}\right)} \tag{68}$$

many times. Here, we note that—as stated in the previous subsection—the auxiliary states σ_E are a function of $\mathcal{C}$. By the probabilistic method, there thus exists at least one circuit $\tilde{\mathcal{C}}$ of this form, with corresponding states $\{\sigma_E\}_E$, such that the average relative entropy is upper-bounded by the same quantity: the circuit's output states

$$\tilde{\rho}_k := \tilde{\mathcal{C}}(|k\rangle\langle k| \otimes |0\rangle\langle 0|) \quad \text{for } k = 0, \dots, N-1, \tag{69}$$

$$\tilde{\rho}_N := \tilde{\mathcal{C}}\left(\sum_E p(E)\frac{\mathbb{I}}{2^n} \otimes \sigma_E\right) \tag{70}$$

for $N = 2^n$, satisfy

$$\frac{1}{N+1}\sum_{k=0}^{N} D(\rho_k || \rho_N) \le c^{\mathcal{O}(n\ell)} = c^{\mathcal{O}(nD/\log^2(n))}. \tag{71}$$

Wrapping up, we plug this into the central lemma (Lemma 1): we lower bound the sample complexity of mitigating the depolarizing noise in the circuit $\mathcal{C}$ by the sample complexity of noisy state discrimination of the states $\{\tilde{\rho}_0, \dots \tilde{\rho}_N\}$. This proceeds in much the same way as we proved Theorem 1, except that we do not even require all the estimates output by weak error mitigation for the state discrimination problem: we may throw away the information in the estimates $\hat{z}_j$ for $j > n$ (i.e., the Paulis on the auxiliary qubits). □

E. Strengthening our results

In this last subsection, we strengthen our results so that they hold for circuits that are restricted to use local gates only. We also improve the depth dependence and introduce a dependence on the light-cone size.

1. Geometrically local circuits

Our circuit construction works by iterating the construction of Ref. [18] D times. The reader may observe, however, that the above construction requires all-to-all connectivity. Bearing in mind that error mitigation is usually applied to near-term quantum circuits for which only some geometrically local architecture is available, we now discuss how our results transfer to error mitigation only on such circuits. We observe that, for architectures in which it is possible to apply gates to nearest neighbors on a d dimensional lattice, it is possible to implement depth D circuit with all-to-all connectivity in depth $\mathcal{O}(Dn^{\frac{1}{d}})$ [19, 20] by performing additional layers of SWAP gates. Note that SWAP gates are Clifford, thus combining these SWAP networks with the exact 2-designs we have used before again give rise to Cliffords. Also note that depth $\mathcal{O}(n^{1/d})$ is necessary to ensure that the lightcone of all qubits corresponds to the whole system.

Thus, unsurprisingly, with limited connectivity, the exponential in n cost of error mitigation implied by our results only kicks in at depths at which the light-cone of all qubits is the entire system. And, for d dimensional architectures, this is $\mathcal{O}(n^{1/d})$. Translating the circuit used for Thm. 5 to a d-dimensional architecture then gives that our results kick-in at $\mathcal{O}(n^{\frac{1}{d}}\log^2(n))$ depth.

Corollary 1 (Error-mitigating circuits composed of 2-local gates in d dimensions with depolarizing noise)**.** *There exists a constant $c \geq 1$, such that for n-qubit, depth-D circuits composed of 2-local gates in d dimensions, at least*

$$m \geq c^{-\tilde{\mathcal{O}}(nD)} \tag{72}$$

copies of the noisy circuit output state are needed to mitigate depolarizing noise of parameter p, once

$$D \geq \tilde{\mathcal{O}}\left(\frac{n^{1/d}}{\log(\frac{4}{1+3p^2})}\right) \quad . \tag{73}$$

2. Smaller depths and light-cone size dependence

Furthermore, we can easily use the construction above to also obtain a large relative entropy contraction at smaller depths. Indeed, suppose we partition the qubits into s disjoint subsets $S_1, \ldots, S_s$ such that each subset has maximal size $L = \lceil n/s \rceil$. After that, we independently apply the construction above *on each* subset. Then at depth

$$D \geq O\left(\frac{\log^2(L)}{\log\left(\frac{4}{1+3p^2}\right)}\right) \tag{74}$$

on each block the relative entropy will satisfy

$$\frac{1}{N+1}\sum_{k=0}^{N} D(\rho_k||\rho_N) \leq O(c^{-LD}). \tag{75}$$

As the state will be product across different subsets, the total relative entropy will be at most

$$\mathcal{O}\left(sc^{-LD}\right). \tag{76}$$

Furthermore, another property this construction enjoys is that it only involves quantum circuits with light-cones of size at most $\lceil n/s \rceil$. Indeed, as each of the unitaries only acts on subset of qubits of this size, the light-cone of each qubit is of that size. Such light-cone size considerations allow us to reformulate our theorem in terms of a customizable parameter s. Choosing s wisely leads to our final theorem with the advertised depth dependence, as follows:

Theorem 4 (Error mitigation with depolarizing noise with small depth and light-cones)**.** *Let $\mathcal{A}$ be an input state-agnostic weak error mitigation algorithm (see Definition 5) that takes as input m noisy copies of the output state of a circuit $\mathcal{C}$ acted upon by local depolarizing noise of parameter p. Suppose that for an arbitrary circuit $\mathcal{C}$ acting on n qubits of depth D, where*

$$D \geq O\left(\frac{\log^2(n/s)}{\log\left(\frac{4}{1+3p^2}\right)}\right) \tag{77}$$

for some $0 < s < n$, and the set of observables $\mathcal{M} = \{Z_i\}_{i=1}^n$, $\mathcal{A}(\mathcal{C}, \mathcal{M}, p)$ is able to produce estimates $\{\hat{z}_i\}_{i\in[n]}$ such that with probability

at least $1-\delta$,

$$|\hat{z}_i - \mathrm{Tr}\left[Z_i \mathcal{C}(\rho)\right]| \leq \epsilon \qquad \textit{for all } i \in [n]. \tag{78}$$

Then as long as $\epsilon < 1/2$*, there is a constant* $c \geq 1$*, such that the number of noisy copies,* m*, needed by* $\mathcal{A}$*, is lower-bounded as*

$$m = \frac{1}{s} c^{-\tilde{\mathcal{O}}(Dn/s)} \quad , \tag{79}$$

where the notation $\tilde{\mathcal{O}}()$ *hides polylogarithmic factors in* n*. In particular, by picking* $s \sim n/\log^2(n)$*, we see that there are circuits of depth poly*$(\log\log(n))$ *that require a super-polynomial number of samples to error-mitigate.*

The theorem above shows that as long as light-cone sizes are slightly above $\log(n)$ (namely $\log^2(n)$) and depths are slightly above constant ($\log^2(\log(n))$, error mitigation is no longer efficient in general. However, note that it is possible to compute the expectation value of observables with light-cones of size $\mathcal{O}(\log(n))$ efficiently classically. This proves that in general error mitigation can only provide a better exponent in the scaling of an algorithm to estimate expectation values when compared with classical algorithms.

Furthermore, also this construction can be applied to the case of geometrically local circuits on a d-dimensional lattice. Note that the above construction consists of a parallel circuits on n/s qubits. Thus, we can implement circuits with all-to-all connectivity *only* on the n/s qubits with a depth overhead of $\mathcal{O}((n/s)^{\frac{1}{d}})$. In particular, for the choice of $s = n/\log(n)^2$, this gives gives overheads in depth of $\mathcal{O}((\log(n))^{\frac{2}{d}})$ for when superpolynomially-many samples are required for error mitigation in d-dimensional circuits.

IV. BEYOND UNITAL NOISE

So far all the results we have discussed only apply to local random circuits with unital noise. It is thus natural to ask how the picture changes when the noise is non-unital. This is because many physically relevant noise models, such as amplitude damping, are non-unital. However, going beyond the non-unital model brings with it several technical complications. First, as we show, in general the noisy circuit will not converge anymore to a fixed point. Second, the entropy of a state does not necessarily increase under a non-unital map. As our previous results were based on entropic considerations, our arguments do not apply anymore. Furthermore, if the outputs of the noisy circuit are not full-rank, as could be the case for noise models like amplitude damping, the relative entropy between the outputs given different inputs might even be infinitely large. This motivates us to look at a different distance measure as well. Indeed, we resort in this section to the standard technique of controlling the trace distance through the Hilbert-Schmidt distance.

In light of these complications, we now discuss a toy model for the convergence of noisy circuits where it is still possible to obtain explicit bounds on their convergence. More precisely, we consider the model in which we have a circuit composed of (global) 2-designs followed by a noisy channel, and then we iterate this construction.

We show below that if the noisy channel is a qubit product channel, in expectation the trace distance between outputs are exponentially small in both the depth and the number of qubits. Although global 2-designs require at least polylogarithmic depth to implement and we only consider the case in which the noise acts after we implement it, we believe that our results illustrate that in principle our conclusions in the previous sections should carry over to the non-unital setting for deep enough unitary circuits. We start by computing moments of noisy random circuits that will be crucial for our conclusions later on.

A. Purity and overlap change after one noisy gate

Our goal is to understand the expected Hilbert-Schmidt distance between two initially-orthogonal pure states that are impacted by a noisy random circuit, in which the noise is potentially non-unital. Specifically, we let the noisy random circuit to be of the form

$$T_D := \bigcirc_{\ell=1}^{D} \mathcal{N} \circ \mathcal{U}_\ell, \tag{80}$$

where $\mathcal{N}$ is an arbitrary quantum channel and the unitary quantum channels $\mathcal{U}_\ell(\cdot) = U_\ell(\cdot)U_\ell^\dagger$ are such that the unitaries U_ℓ are Haar-random. (We will see below that it suffices that the U_ℓ come from a unitary 2-design.) The idea is that $\mathcal{N}$ represents the noise after we apply the gate. Then, we are interested in the output states

$$\rho_{i,D} := T_D(\rho_i), \quad i \in \{1,2\}, \tag{81}$$

where ρ_1 and ρ_2 are arbitrary states of a d-dimensional system. In particular, we are interested in how the quantity

$$\mathbb{E}_{U_1,\ldots,U_D}[\|\rho_{1,D} - \rho_{2,D}\|_2^2] = \mathbb{E}_U[\mathrm{Tr}(\rho_{1,D}^2) + \mathrm{Tr}(\rho_{2,D}^2) - \mathrm{Tr}(\rho_{1,D}\rho_{2,D}) - \mathrm{Tr}(\rho_{2,D}\rho_{1,D})] \tag{82}$$

decays with respect to the depth D. Note that by controlling overlaps and purities it is then straightforward to control the Hilbert-Schmidt distance.

As a first step, let us study the above question for $D = 1$. We consider the states $\rho_{i,1} = \mathcal{N} \circ \mathcal{U}(\rho_i)$, $i \in \{1,2\}$, and we would like to compute the expected value of the overlap $\mathrm{Tr}[\rho_{1,1}\rho_{2,1}]$. We can express this overlap in a more convenient way, namely,

$$\begin{aligned} \mathrm{Tr}[\rho_{1,1}\rho_{2,1}] &= \mathrm{Tr}[(\mathcal{N} \circ \mathcal{U})(\rho_1)(\mathcal{N} \circ \mathcal{U})(\rho_2)] \\ &= \mathrm{Tr}[F\left((\mathcal{N} \circ \mathcal{U})(\rho_1) \otimes (\mathcal{N} \circ \mathcal{U})(\rho_2)\right)], \end{aligned} \tag{83}$$

where F is the swap operator. Now, suppose that $\mathcal{N}$ has the Kraus decomposition

$$\mathcal{N}(\cdot) = \sum_{\ell=1}^{r} K_\ell(\cdot)K_\ell^\dagger. \tag{84}$$

Using this, we get that the expected overlap of the output states is

$$\mathbb{E}_U[\mathrm{Tr}[\rho_{1,1}\rho_{2,1}]] = \sum_{\ell,\ell'=1}^{r} \mathbb{E}_U\left[\mathrm{Tr}\left[F\Big((K_\ell\otimes K_{\ell'})(U\otimes U)(\rho_1\otimes\rho_2)(U^\dagger\otimes U^\dagger)(K_\ell^\dagger\otimes K_{\ell'}^\dagger)\Big)\right]\right] \tag{85}$$

$$= \alpha \sum_{\ell,\ell'=1}^{r} \mathrm{Tr}\left[F((K_\ell\otimes K_{\ell'})(K_\ell^\dagger\otimes K_{\ell'}^\dagger))\right] + \beta \sum_{\ell,\ell'=1}^{r} \mathrm{Tr}\left[F(K_\ell\otimes K_{\ell'})F(K_\ell^\dagger\otimes K_{\ell'}^\dagger)\right], \tag{86}$$

where for the last line we have used the general fact (see, e.g., Ref. [21, Example 7.25]) that

$$\mathbb{E}_U[(U\otimes U)X(U^\dagger\otimes U^\dagger)] = \underbrace{\left(\frac{\mathrm{Tr}[X]}{d^2-1} - \frac{\mathrm{Tr}[FX]}{d(d^2-1)}\right)}_{\alpha}\mathbb{I}\otimes\mathbb{I} + \underbrace{\left(\frac{\mathrm{Tr}[FX]}{d^2-1} - \frac{\mathrm{Tr}[X]}{d(d^2-1)}\right)}_{\beta}F. \tag{87}$$

We now observe that, because only two copies of the unitary U appear in the expectation value, we would get the same quantity if we took the expectation value with respect to a unitary 2-design instead of the Haar-random unitaries.

Now, the involved quantities in (86) take the values

$$\mathrm{Tr}\left[F((K_\ell\otimes K_{\ell'})(K_\ell^\dagger\otimes K_{\ell'}^\dagger))\right] = \mathrm{Tr}[K_\ell K_\ell^\dagger K_{\ell'}K_{\ell'}^\dagger], \tag{88}$$

$$\mathrm{Tr}\left[F(K_\ell\otimes K_{\ell'})F(K_\ell^\dagger\otimes K_{\ell'}^\dagger)\right] = \mathrm{Tr}[K_{\ell'}K_\ell^\dagger\otimes K_\ell K_{\ell'}^\dagger] = |\,\mathrm{Tr}[K_\ell K_{\ell'}^\dagger]|^2, \tag{89}$$

for all $\ell,\ell'\in\{1,2,\dots,r\}$ and

$$\alpha = \frac{1}{d^2-1} - \frac{\mathrm{Tr}[\rho_1\rho_2]}{d(d^2-1)}, \tag{90}$$

$$\beta = \frac{\mathrm{Tr}[\rho_1\rho_2]}{d^2-1} - \frac{1}{d(d^2-1)}. \tag{91}$$

We can rephrase these quantities in terms of global properties of the channel as

$$\sum_{\ell=1}^{r} K_\ell K_\ell^\dagger = \mathcal{N}(\mathbb{I}), \tag{92}$$

$$\sum_{\ell,\ell'=1}^{r} |\,\mathrm{Tr}[K_\ell K_{\ell'}^\dagger]|^2 = \mathrm{Tr}[(\Gamma^{\mathcal{N}})^2], \tag{93}$$

where

$$\Gamma^{\mathcal{N}} := (\mathrm{id}\otimes\mathcal{N})(|\Gamma\rangle\langle\Gamma|), \quad |\Gamma\rangle := \sum_{j=0}^{d-1}|j,j\rangle, \tag{94}$$

is the Choi representation of $\mathcal{N}$. Therefore, the expected overlap of the output states is

$$\mathbb{E}_U[\mathrm{Tr}[\rho_{1,1}\rho_{2,1}]] = \left(\frac{1}{d^2-1} - \frac{\mathrm{Tr}[\rho_1\rho_2]}{d(d^2-1)}\right)\mathrm{Tr}[\mathcal{N}(\mathbb{I})^2] + \left(\frac{\mathrm{Tr}[\rho_1\rho_2]}{d^2-1} - \frac{1}{d(d^2-1)}\right)\mathrm{Tr}[(\Gamma^{\mathcal{N}})^2]. \tag{95}$$

Note that both $\mathrm{Tr}[(\Gamma^{\mathcal{N}})^2]$ and $\mathrm{Tr}[\mathcal{N}(\mathbb{I})^2]$ scale with the underlying dimension of the space. In order to more easily grasp the scaling of the formula above, it is fruitful to look at the rescaled quantities

$$\eta = \mathrm{Tr}[\mathcal{N}(\mathbb{I}/d)^2], \quad \nu = \mathrm{Tr}[(\Gamma^{\mathcal{N}})^2/d^2]. \tag{96}$$

Note that η is just the purity of the output on the maximally mixed state and, thus, takes values in $[d^{-1},1]$, whereas ν is just the purity of the output on the maximally entangled state if we act with the channel on one half of the system (i.e., the Choi state) and takes values in $[d^{-2},1]$. In terms of these newly defined quantities, we obtain

$$\mathbb{E}_U[\mathrm{Tr}[\rho_{1,1}\rho_{2,1}]] = \left(\frac{d^2}{d^2-1} - \frac{d\,\mathrm{Tr}[\rho_1\rho_2]}{(d^2-1)}\right)\eta + \left(\frac{d^2\,\mathrm{Tr}[\rho_1\rho_2]}{d^2-1} - \frac{d}{(d^2-1)}\right)\nu. \tag{97}$$

Remark 5. *It is well-known that twirling any quantum channel with a unitary* 2*-design will lead to a global depolarizing noise in expectation,*

i.e., $\mathbb{E}_{U\sim\text{Haar}}\big[\mathcal{U}^\dagger \circ \mathcal{N} \circ \mathcal{U}\big] = \mathcal{D}_p^{(d)}$*. Note, however, that the problem at hand is slightly different, as we are interested in an expectation value that contains two copies of the channel. In that case, the resulting action cannot be effectively of a global depolarizing channel. To illustrate this point, assume that the noise* $\mathcal{N}$ *is actually a unitary. In that case, the purity or overlap between two different initial states should not change after the action of* $\mathcal{U}^\dagger \circ \mathcal{N} \circ \mathcal{U}$*, which would not be the case if the resulting channel were that of depolarizing noise. A simple instantiation of this is when the two initial states are actually identical:* $\rho_1 = \rho_2 = \rho$*; then plug in* $\mathcal{N}(\mathbb{I}) = \mathbb{I}$ *and* $\mathrm{Tr}[(\Gamma^{\mathcal{N}})^2] = d^2$ *to our earlier calculation, obtaining*

$$\begin{aligned}\mathbb{E}_U[\mathrm{Tr}[\rho_{1,1}\rho_{2,1}]] &= \alpha d + \beta d^2 \\ &= d\left(\frac{1}{d^2-1} - \frac{\mathrm{Tr}[\rho^2]}{d(d^2-1)}\right) + d^2\left(\frac{\mathrm{Tr}[\rho^2]}{d^2-1} - \frac{1}{d(d^2-1)}\right) \\ &= \mathrm{Tr}[\rho^2],\end{aligned} \tag{98}$$

as expected.

B. Convergence of global random circuits

From Eq. (97), we obtain a recursive formula for the overlap and/or purity of the output states $\rho_{i,D}$, $i \in \{1,2\}$, as defined in (81). We have

$$\begin{aligned}\mathbb{E}_{U_1,\ldots,U_D}[\mathrm{Tr}[\rho_{1,D}\rho_{2,D}]] &= \left(\frac{d^2}{d^2-1} - \frac{d\,\mathrm{Tr}[\rho_{1,D-1}\rho_{2,D-1}]}{(d^2-1)}\right)\eta + \left(\frac{d^2\,\mathrm{Tr}[\rho_{1,D-1}\rho_{2,D-1}]}{d^2-1} - \frac{d}{(d^2-1)}\right)\nu \\ &= \frac{d(d\nu-\eta)}{d^2-1}\mathbb{E}_{U_1,\ldots,U_{D-1}}[\mathrm{Tr}[\rho_{1,D-1}\rho_{2,D-1}]] + \frac{d(d\eta-\nu)}{d^2-1}.\end{aligned} \tag{99}$$

The structure of the recursion becomes more apparent when discussed in abstract terms. Letting $A := \frac{d(d\nu-\eta)}{d^2-1}$, $B := \frac{d(d\eta-\nu)}{d^2-1}$, and $f(D) := \mathbb{E}_{U_1,\ldots,U_D}[\mathrm{Tr}[\rho_{1,D}\rho_{2,D}]]$, we have that

$$\begin{aligned}f(D) &= Af(D-1) + B \\ &= A(Af(D-2)+B) + B \\ &= A^2 f(D-2) + AB + B \\ &= A^2(Af(D-3)+B) + (A+1)B \\ &= A^3 f(D-3) + (A^2+A+1)B \\ &= A^D f(0) + (A^{D-1} + \cdots + A + 1)B.\end{aligned} \tag{100}$$

The right term is nothing but a geometric series, which, noting that $0 \le A < 1$, yields

$$f(D) = A^D f(0) + \frac{1-A^D}{1-A}B. \tag{101}$$

Applied to our setting, we obtain

$$\mathbb{E}_{U_1,\ldots,U_D}[\mathrm{Tr}[\rho_{1,D}\rho_{2,D}]] = \left(\frac{d(d\nu-\eta)}{d^2-1}\right)^D \mathrm{Tr}[\rho_1\rho_2] + \left[1 - \left(\frac{d(d\nu-\eta)}{d^2-1}\right)^D\right]\frac{d(d\eta-\nu)}{d^2-1-d(d\nu-\eta)}. \tag{102}$$

The exponential behavior in D of this quantity and the applicability of the geometric series is determined by the term $d(d\nu-\eta)/(d^2-1)$. The fact that this term is always positive and strictly smaller than one if the channel is not unitary, indicating an exponential decay in D, is a consequence of the following proposition.

Proposition 2. *Let* $\mathcal{N} : \mathsf{M}_d \to \mathsf{M}_d$ *be a quantum channel. Define* η *and* ν *as in Eq. (96). Then*

$$d\nu - \eta > 0. \tag{103}$$

Furthermore,

$$\frac{d^2\nu - d\eta}{d^2-1} \le 1 \tag{104}$$

with equality if and only if $\mathcal{N}$ *is unitary.*

Proof. First, note that for any linear superoperator $\mathcal{L}$ we have $\mathrm{Tr}[|\Gamma\rangle\langle\Gamma|(\mathrm{id}\otimes\mathcal{L})(|\Gamma\rangle\langle\Gamma|)] = \mathrm{Tr}[\mathcal{L}]$,where by $\mathrm{Tr}[\mathcal{L}]$ we mean the trace of $\mathcal{L}$ as a linear map. Thus, we see that $\nu = d^{-2}\,\mathrm{Tr}[\mathcal{N}^\dagger\mathcal{N}]$ is just the average of the square of the singular values of quantum channel. Now, note that $d^{-\frac{1}{2}}\mathbb{I}$ is a normalized vector with respect to the Hilbert-Schmidt norm. Furthermore, let X_i, $1 \le i \le d^2-1$ be an orthonormal, self-adjoint

basis for the space of traceless matrices. Then, using the fact that

$$|\Gamma\rangle\langle\Gamma| = \frac{\mathbb{I}}{\sqrt{d}} \otimes \frac{\mathbb{I}}{\sqrt{d}} + \sum_{i=1}^{d^2-1} \overline{X_i} \otimes X_i, \tag{105}$$

we obtain

$$\nu = d^{-2}\operatorname{Tr}[\mathcal{N}^\dagger \mathcal{N}] = d^{-2}\operatorname{Tr}[\mathcal{N}(d^{-\frac{1}{2}}I)^2] + d^{-2}\sum_{i=1}^{d^-1}\operatorname{Tr}[\mathcal{N}(X_i)^2] = d^{-1}\eta + d^{-2}\sum_{i=1}^{d^2-1}\operatorname{Tr}[\mathcal{N}(X_i)^2]. \tag{106}$$

As the second term in the sum above is clearly positive, we obtain the claim in Eq. (103). To obtain the claim in Eq. (104), note that the expression is clearly monotone increasing in ν and decreasing in η. The maximum value for ν is 1 because it is a purity, and this value is attained on a unitary channel (pure Choi states are unitaries). Thus, the quantity in Eq. (104) is strictly smaller than 1 for non-unitary channels, as then $\nu < 1$. Furthermore, for unitary channels we have also have $\eta = d^{-1}$, and, thus, the quantity in (104) is 1 for unitary channels. □

In particular, it follows that unless the channel is unitary, the expected trace norm between outputs will be exponentially small. To conclude that, we will first study the convergence in the Hilbert-Schmidt norm.

Proposition 3. *Let $T_D = \bigcirc_{\ell=1}^{D} \mathcal{N} \circ \mathcal{U}_\ell$ be a random quantum channel where $\mathcal{N} : \mathsf{M}_d \to \mathsf{M}_d$ is a fixed quantum channel and $\mathcal{U}_\ell$ are drawn independently from a unitary 2-design on a d-dimensional space. Furthermore, let η, ν be defined as in Eq. (96) and*

$$q = \frac{d(d\nu - \eta)}{d^2 - 1}. \tag{107}$$

Then, for any orthogonal pure states ρ_1, ρ_2 we have

$$\mathbb{E}_{U_1,\ldots,U_D}\left[\|T_D(\rho_1 - \rho_2)\|_2^2\right] = 2q^D \tag{108}$$

and

$$\mathbb{E}_{U_1,\ldots,U_D}[\|T_D(\rho_1 - \rho_2)\|_1] \le \sqrt{2dq^D}. \tag{109}$$

Proof. First, observe that

$$\mathbb{E}_{U_1,\ldots,U_D}\left[\|T_D(\rho_1 - \rho_2)\|_2^2\right] = \mathbb{E}_{U_1,\ldots,U_D}[\operatorname{Tr}[\rho_{1,D}^2]] + \mathbb{E}_{U_1,\ldots,U_D}[\operatorname{Tr}[\rho_{2,D}^2]] - 2\mathbb{E}_{U_1,\ldots,U_D}[\operatorname{Tr}[\rho_{1,D}\rho_{2,D}]], \tag{110}$$

where $\rho_{i,D} = T_D(\rho_i)$, $i \in \{1,2\}$. Inserting the expression in Eq. (102) for each term, we get

$$\mathbb{E}_{U_1,\ldots,U_D}\left[\|T_\ell(\rho_1 - \rho_2)\|_2^2\right] = q^D\left(\operatorname{Tr}[\rho_1^2] + \operatorname{Tr}[\rho_2^2] - 2\operatorname{Tr}[\rho_1\rho_2]\right), \tag{111}$$

as the rest of the terms cancel. Using that $\operatorname{Tr}[\rho_1\rho_2] = 0$ and $\operatorname{Tr}[\rho_1^2] = \operatorname{Tr}[\rho_2^2] = 1$ we arrive at Eq. (108). Next, from Jensen's inequality, it follows that

$$\mathbb{E}\left[\|T_D(\rho_1 - \rho_2)\|_1\right]^2 \le \mathbb{E}\left[\|T_D(\rho_1 - \rho_2)\|_1^2\right]. \tag{112}$$

Furthermore, we have the standard inequality $\|X\|_1 \le \sqrt{d}\|X\|_2$ for all Hermitian X. Combining this observation with the claim in Eq. (112) yields Eq. (109). □

Let us investigate the case of $d = 2^n$ (n qubits) and $\mathcal{N} = \bigotimes_{i=1}^n \mathcal{N}_i$ (i.e., $\mathcal{N}$ is a product channel) in a bit more detail. Furthermore, assume for simplicity that all $\mathcal{N}_i$ are the same. As the purity is multiplicative, we obtain $\eta_{\mathcal{N}} = \eta_{\mathcal{N}_i}^n$, $\nu_{\mathcal{N}} = \nu_{\mathcal{N}_i}^n$ and

$$q_n = \frac{2^{2n}\nu_{\mathcal{N}_i}^n - 2^n\eta_{\mathcal{N}_i}^n}{2^{2n} - 1}. \tag{113}$$

Thus, we see that the 2-norm decays exponentially in the number of qubits, even after one layer. In particular, we obtain the following corollary.

Corollary 2. *In the same setting of Proposition 3, assume further that $\mathcal{N}$ is an n-qubit product channel and define q_n as in Eq. (113). Then we have*

$$\mathbb{E}_{U_1,\ldots,U_D}[\|T_D(\rho_1 - \rho_2)\|_1] \le 2^{\frac{n+1}{2}} q_n^{\frac{D}{2}}. \tag{114}$$

Proof. The statement follows from Proposition 3 and the discussion above. □

We can combine the statement above with sub-additivity of the trace distance, the triangle inequality and a hybrid argument to conclude that

for m copies of the outputs of the noisy circuit we have

$$\mathbb{E}_{U_1,\ldots,U_D}\left[\|T_D^{\otimes m}(\rho_1^{\otimes m}-\rho_2^{\otimes m})\|_1\right] \leq m 2^{\frac{n+1}{2}} \left(\frac{2^{2n}\nu_{\mathcal{N}_i}^n - 2^n \eta_{\mathcal{N}_i}^n}{2^{2n}-1}\right)^{\frac{D}{2}}. \tag{115}$$

As explained before, successful error mitigation can be used to distinguish orthogonal input states. However, by the Holevo-Helstrom theorem [22, 23], the trace distance bounds the probability of successfully distinguishing between two states. That is, we find the following theorem.

Theorem 6 (Holevo-Helstrom [22, 23]). *The success probability to discriminate two mixed states represented by ρ_1 and ρ_2 is at most*

$$\frac{1}{2}+\frac{1}{2}\left(\frac{1}{2}\|\rho_1-\rho_2\|_1\right).$$

Thus, we conclude the following.

Theorem 7 (Error mitigation with non-unital noise). *Any input agnostic error mitigation procedure that works successfully for outputs of T_D would require a number of copies m that grows as*

$$m \geq 2^{-\frac{n+1}{2}} \left(\frac{2^{2n}\nu_{\mathcal{N}_i}^n - 2^n \eta_{\mathcal{N}_i}^n}{2^{2n}-1}\right)^{-\frac{D}{2}}. \tag{116}$$

As discussed before, $\nu_{\mathcal{N}_i} < 1$ unless we have a unitary channel. Thus m will be $\Omega(c^{-nD})$ for some some $c<1$ at a number of iterations $D=\mathcal{O}(1)$, as

$$\left(\frac{2^{2n}\nu_{\mathcal{N}_i}^n - 2^n \eta_{\mathcal{N}_i}^n}{2^{2n}-1}\right)^{\frac{D}{2}} \leq 2^{-\frac{n+1}{2}} \tag{117}$$

for $D=\mathcal{O}(1)$.

The input aware case is a bit more subtle. What we conclude from the results above is that all input states will be mapped by T_D to the same state up to an exponential small correction. Thus, the noisy circuit is essentially a replacer channel and any error mitigation algorithm with sub-exponential samples would have the same performance given copies of this one state. However, unlike the case with the maximally mixed state, it is unclear that it is possible to classically simulate that state or that it is not a valuable computational resource. However, it is of course also unclear how a practical error mitigation might profit from access to samples from an (unknown) fixed state.

C. Bounds on the probability of successful virtual distillation

It is straightforward to use the result introduced above to bound the probability of virtual distillation protocols [2, 3] succeeding under non-unital noise. As discussed before, the probability of such protocols succeeding is bounded by $\mathrm{Tr}[\rho^2]$. If we introduce the quantity

$$r_n = \frac{2^{2n}\eta_{\mathcal{N}_i}^n - 2^n \nu_{\mathcal{N}_i}^n}{2^{2n}-1}, \tag{118}$$

we can show the following:

Proposition 4. *In the same setting of Proposition 3, assume further that $\mathcal{N}$ is an n-qubit product channel and define q_n as in Eq. (113) and r_n as in Eq. (118). Then we have for all pure states ρ*

$$\mathbb{E}_{U_1,\ldots,U_D}\left[\mathrm{Tr}\left[T_D(\rho)^2\right]\right] = \left(q_n^D + \frac{(1-q_n^D)r_n}{1-q_n}\right). \tag{119}$$

Furthermore, both r_n and q_n are exponentially small in n unless the quantum channel $\mathcal{N}_i$ is unitary or a replacer channel for a pure state, i.e., $\mathcal{N}_i(\sigma)=|\psi\rangle\langle\psi|$ for all σ and some $|\psi\rangle$.

Proof. The claim in Eq. (119) follows from Eq. (102), inserting the definition of q_n and r_n. As $q_n \leq \frac{\nu_{\mathcal{N}_i}^n}{1-2^{-2n}}$ and $\nu_{\mathcal{N}_i}=1$ if, and only if, $\mathcal{N}_i$ is a unitary channel, we see that q_n will decay exponentially unless the channel is unitary. Let us now analyse r_n, assuming the channel is not unitary. Note that it will also decay exponentially if we manage to show that $\eta_{\mathcal{N}_i}<1$ if the channel is not a replacer with a pure state. As $\eta_{\mathcal{N}_i}$ is the purity of the output of the maximally mixed state, if $\eta_{\mathcal{N}_i}=1$ then the maximally mixed state is mapped to a pure state $|\psi\rangle\langle\psi|$. We will now show that this implies that all states are mapped to the same pure state. By linearity, it suffices to show the claim for a pure input state $|\phi\rangle\langle\phi|$. For this, note that

$$\frac{1}{2}|\phi\rangle\langle\phi| \leq \frac{\mathbb{I}}{2} \implies \frac{1}{2}\mathcal{N}_i(|\phi\rangle\langle\phi|) \leq \mathcal{N}_i\left(\frac{\mathbb{I}}{2}\right) = |\psi\rangle\langle\psi|.$$

The fact that $\frac{1}{2}\mathcal{N}_i(|\phi\rangle\langle\phi|) \leq |\psi\rangle\langle\psi|$ implies that $\frac{1}{2}\mathcal{N}_i(|\phi\rangle\langle\phi|) = \frac{1}{2}|\psi\rangle\langle\psi|$. Indeed, if it was not the case, $\mathcal{N}_i(|\phi\rangle\langle\phi|)$ would have nonzero support on the subspace orthogonal to $|\psi\rangle$, which would contradict the inequality. Thus, we conclude that $\eta_{\mathcal{N}_i} < 1$ for non-replacer channels and hence that r_n is exponentially small in n. □

Thus, it follows that the probability of success of virtual distillation, which is upper-bounded by Eq. (119) will also be exponentially small even after *a single* layer of noise unless we are in the unitary or replacer case. But for unitary errors virtual distillation does not have any effect, as the input state is anyways pure. And for replacer channels it is clear that, even though the procedure will succeed with probability 1, we will just output a fixed pure product state. So in the cases where the success probability is not exponentially small, the procedure is of no use. However, note that it will not converge to the minimal possible value of the purity (2^{-n}) unless the channel is unital, but rather to $r_n/(1-q_n)$. This is to be expected, as the noisy quantum circuit will not converge to the maximally mixed state in this case.

Let us now briefly compute the involved quantities for the case of local depolarizing noise on n qubits. As this is a unital channel, we have that $\eta = 2^{-n}$. To compute ν, note that

$$(\mathcal{D}_p \otimes \mathrm{id})(|\Omega\rangle\langle\Omega|) = (1-p)\frac{I}{4} + p|\Omega\rangle\langle\Omega|, \tag{120}$$

where $|\Omega\rangle := \frac{1}{\sqrt{2}}|\Gamma\rangle$. It is then not difficult to check that the purity of this state is given by $3p^2 + 1/4$. Inserting this expression into the definition of q_n we get

$$q_n = \frac{(3p^2+1)^n - 1}{4^n - 1}. \tag{121}$$

V. RELATIONSHIP BETWEEN DIFFERENT METHODS OF QUANTUM ERROR MITIGATION

We here follow up on the discussion in the Methods section relating different methods of quantum error mitigation.

A. Weak error mitigation implies strong error mitigation only with exponentially-many observables

Let us now consider the problem of using the outputs of a weak error mitigation algorithm to obtain a strong error mitigation algorithm. That is, what is the minimum number of expectation values required to produce samples from the noiseless circuit? Here we allow arbitrarily complex post-processing steps because our focus is on sample complexity, or fundamental information-theoretic limits. Restricting our focus to bounded-time (i.e., realistic) computations would only further limit the set of protocols under consideration.

First note that in the limit of *exponentially* many error-mitigated expectation values, obtaining a (potentially inefficient) strong error mitigation algorithm is possible. Indeed, suppose we were able to perform weak error mitigation, outputting estimates of all n-qubit Paulis

$$\mathrm{Tr}(\mathcal{C}(\rho)P_i) \qquad \forall P_i \in \mathcal{Q}_n \tag{122}$$

up to exponentially small precision. This allows us to perform full tomography on the noiseless output $\mathcal{C}(\rho)$—if we were allowed to query exponentially-many such expectation values (since there are exponentially many members of $\mathcal{Q}_n$). However, this procedure would clearly be inefficient and likely more costly than just simulating the circuit classically. Thus, the more interesting question is: **could there be an algorithm that only needs to see *polynomially*-many expectation values from the output of weak error-mitigation to obtain strong error mitigation?**

We proceed to provide a partial negative answer to this question, showing that there cannot exist such an algorithm if the expectation values are all of observables *diagonal in the same eigenbasis*. To do so, we showcase an instance of weak error mitigation that provides an explicit counterexample to the conjecture. First, we will need to introduce the notion of a *statistical query* [24]:

Definition 2 (Statistical query)**.** *A statistical query is a pair (q, τ) with*

- *a function $q : \{0,1\}^n \times \{0,1\} \to \{0,1\}$.*
- *τ: a tolerance parameter $\tau \geq 0$.*

Now we are ready to define a statistical query oracle.

Definition 3 (Statistical query oracle)**.** *Fix an unknown function $c : \{0,1\}^n \to \{0,1\}$ and a probability distribution D over the domain $\{0,1\}^n$. The statistical query oracle, $SQ(q,\tau)$, when given a statistical query, returns any value in the range as*

$$\left[\mathbb{E}_{x\sim D}[q(x,c(x))] - \tau, \mathbb{E}_{x\sim D}[q(x,c(x))] + \tau\right]. \tag{123}$$

We will also need to use the observation that the problem of PARITIES on n bits (which we now define) can be solved with $\mathrm{poly}(n)$ samples but requires $\exp(n)$ statistical queries.

Definition 4 (PARITIES)**.** *The class of* PARITIES *is the set of functions $\{c_s : \{0,1\}^n \to \{0,1\}\}_{s\in\{0,1\}^n}$ where*

$$c_s(x) = x \cdot s \quad \textit{for } s \in \{0,1\}^n \quad . \tag{124}$$

For every such c_s, we may define the associated distribution $P_s : \{0,1\}^{n+1} \to [0,1]$ via

$$P_s(x \bowtie y) = \begin{cases} 2^{-n}\,, & \textit{if} \quad y = x \cdot s \\ 0\,, & \textit{else}\,, \end{cases} \tag{125}$$

where $x \in \{0,1\}^n$, $y \in \{0,1\}$, and the symbol $\bowtie$ means concatenation. That is, P_s is the distribution which is supported uniformly on those bit-strings whose last bit is the parity of the subset of the first n bits that are indexed by the 'secret' n-bit string s.

There are three important facts about PARITIES that we will use.

1. For every $s \in \{0,1\}^n$, there exists an $n+1$-qubit quantum circuit $\mathcal{C}_s$ that 'encodes' the distribution P_s, in the following sense: when initialized on the all-0s state, the output state has support only on the computational basis states whose labels are in the support of the distribution P_s:
$$\mathcal{C}_s \left|0\right\rangle^{\otimes n+1} = \frac{1}{\sqrt{2^n}} \sum_{x \in \{0,1\}^n} \left|x \bowtie c_s(x)\right\rangle \quad , \tag{126}$$
for the parity function $c_s(x) = x \cdot s$. This has been proven in, for example, Ref. [25].

2. PARITIES are not solvable with Clifford data regression sub-exponentially-many statistical queries [26]:

   Lemma 7 (SQ-hardness of PARITIES [26])**.** *Any learning algorithm that is restricted to make statistical queries of the form (χ, τ), where $\tau \geq \tau_0$ for each query, and for all $c_s \in$ PARITIES, and all D, is able to output a hypothesis $h : \{0,1\}^n \to \{0,1\}$ such that $\mathbb{P}_{x \sim D}[h(x) \neq c_s(x)] \leq \epsilon$ must make $\Omega(\tau_0^2 \cdot 2^n)$ queries.*

3. We can lower-bound the total variation distance between any two PARITIES distributions as follows.

   Lemma 8 (Parity distributions)**.** *For any two $s, s' \in \{0,1\}^k$, $d_{TV}(P_s, P_{s'}) \geq 1/2$.*

   Proof. Le Cam's two point method says that the failure probability of any binary hypothesis test $\Psi : X \to \{P, Q\}$ on two distributions $P, Q : X \to [0,1]$ each chosen with probability $1/2$, is lower bounded as
$$\min_{\Psi} P_{\text{error}}(\Psi) \geq \frac{1 - d_{TV}(P,Q)}{2} \quad . \tag{127}$$
   For any two strings $s, s' \in \{0,1\}^k$, let $P = P_s$ and $Q = P_{s'}$. We wish to bound $d_{TV}(P_s, P_{s'})$. To do so we will exhibit a hypothesis test on P_s versus $P_{s'}$ that has error probability $1/4$. Plugging this into Eq. (127) yields the desired lower bound on $d_{TV}(P_s, P_{s'})$. A simple test is then as follows: given a string $x \bowtie c$ drawn from either P_s or $P_{s'}$, where c is one bit, we compute the parity of x with s and s'. In Case 1, we have $x \cdot s = x \cdot s'$. Then we output s or s' uniformly at random. This succeeds with probability $1/2$. Otherwise (call this Case 2) $x \cdot s \neq x \cdot s'$. Then we output whichever string s or s' yields the right parity c. This succeeds with probability 1 in distinguishing the two distributions. So the probability of error of the test we have described is simply
$$P_{\text{error}} = P(\text{Case } 1)1/2 \quad . \tag{128}$$
   Now let us compute $P(\text{Case } 1) = 1 - P(\text{Case } 2)$. As the x part of the string is chosen uniformly from $\{0,1\}^n$, computing $P(\text{Case } 2)$ boils down to computing the number of x's that either satisfy the equations $x \cdot s = 0$ and $x \cdot s' = 1$ or $x \cdot s = 1$ and $x \cdot s' = 0$. In both cases, x must satisfy two linearly independent relations. Thus, we conclude that the strings that satisfy $x \cdot s = 0$ and $x \cdot s' = 1$ form a vector space of dimension $n-2$, which contains 2^{n-2} strings. Thus, the number of strings that satisfy either $x \cdot s = 0$ and $x \cdot s' = 1$ or $x \cdot s = 1$ and $x \cdot s' = 0$ is 2^{n-1} which is half of all the strings x. Thus, $P(\text{Case } 2) = P(\text{Case } 1) = 1/2$, and so the overall $P_{\text{error}} = 1/2 * 1/2 = 1/4$. □

In addition, we will need the notion of hypothesis selection and an algorithm that achieves a good approximation guarantee for it, due to Yatracos [27]:

Theorem 8 (3-proper hypothesis selection)**.** *Fix a class of distributions $\mathcal{Q} = \{q_1, \ldots, q_k\}$ and $\epsilon, \delta > 0$. Given $O(\log|\mathcal{Q}|/\epsilon^2)$ samples from a target distribution p (which may not be in $\mathcal{Q}$), there is an algorithm to output a distribution $q^* \in \mathcal{Q}$ satisfying*

$$d_{TV}(p, q^*) \leq 3 \min_{i \in [k]} d_{TV}(p, q_i) + \epsilon \tag{129}$$

with probability at least $1 - \delta$.

Here, the word *proper* refers to the fact that the output distribution is required to be in $\mathcal{Q}$, a feature that we will require for the argument we are about to make. The connection between expectation values (the output of weak error mitigation) and statistical queries is encapsulated in the following observations (where for $b \in \{0,1\}^n$, $Z^b := Z^{b_1} \otimes Z^{b_2} \otimes \cdots \otimes Z^{b_n}$):

Lemma 9 (Weak error mitigation on the circuits $\mathcal{C}_s$ outputs statistical queries to the PARITIES_s distribution)**.** *Consider error mitigation on the $n+1$-qubit PARITIES circuit $\mathcal{C}_s$ (see Def. 4). That is, let $\mathcal{M} = \{(\mathbb{I} + Z^b)/2\}_{b \in \{0,1\}^{n+1}}$, and suppose we have a weak error mitigation algorithm $\mathcal{A}(\mathcal{C}_s, \mathcal{N}, \mathcal{M})$ that, with probability $1-\delta$, outputs τ-accurate estimates*

$$|\hat{o}_i - \text{Tr}[\mathcal{C}_s(|0\rangle\langle 0|^{\otimes n+1}) O_b)]| < \tau \qquad \textit{for all } O_b \in \mathcal{M} \quad . \tag{130}$$

The outputs $\hat{o}_i$ are valid responses to statistical queries with tolerance τ for the distribution P_s.

The observation that weak error mitigation outputs statistical queries has also been made in Ref. [28] which introduces the notion of a *quantum statistical query* (QSQs) in the quantum PAC learning setting. We will come back to this after we state our main theorem.

Proof. We will prove that each τ-accurate expectation value output by $\mathcal{A}$ that satisfies Eq. (130) takes the form of a response of a statistical query oracle to a statistical query of tolerance τ, by specifying what c, q, D from Definition 3 correspond to. Let us denote the circuit's clean output state vector as $\mathcal{C}_s |0\rangle^{n+1} = |\psi\rangle = 2^{-n/2} \sum_{x\in\{0,1\}^n} |x \bowtie c_s(x)\rangle$. Then it is easy to see, from inspecting the basis vectors in the support of $|\psi\rangle$, that the desired expectation values can be rewritten as

$$\begin{aligned}\mathrm{Tr}(|\psi\rangle\langle\psi|(\mathbb{I} + Z^b)/2) &= \mathbb{E}_{z\sim P_s}[q_b(z)] \\ &= \mathbb{E}_{x\sim \mathsf{Unif}\{0,1\}^n}[q_b(x, c_s(x))]\end{aligned} \tag{131}$$

where $q_b : \{0,1\}^{n+1} \to \{0,1\}$ is defined as

$$q_b(z) = \frac{1 + \langle z|Z^b|z\rangle}{2} \quad . \tag{132}$$

That is, $q = q_b, c = c_s$ and $D = \mathsf{Unif}\{0,1\}^n$. □

The re-scaling of the observables Z^b is cosmetic; it is merely to obtain a set of observables whose eigenvalues take values in $\{0,1\}$ due to the way we have defined statistical queries in Def. 3. In a similar fashion, while this lemma is written for the observables Z^b, one could write an analogous lemma for observables that are all diagonal in some other common eigenbasis, by simply applying the corresponding basis change at the end of the circuit $\mathcal{C}_s$ and re-scaling the value of the tolerance parameter τ for the statistical query.

This observation will be crucial to the main theorem of this section (Theorem 9), which states that with sub-exponentially-many expectation values of observables which share the same eigenbasis, weak error mitigation cannot imply strong error mitigation in the worst-case. We now state this more formally:

Theorem 9 (Exponentially-many expectation values of observables in the same eigenbasis are required to output samples from that basis)**.** *There is a class of n-qubit circuits, such that for every circuit $\mathcal{C}$ in the class (denote the clean circuit output state vector as $|\psi\rangle = \mathcal{C}(|0\rangle)$) and the set of observables $\mathcal{M} = \{Z^b\}_{b\in\{0,1\}^{n+1}}$ with $m = o(\tau^2 \cdot 2^n)$, the following holds:*

No algorithm $\mathcal{B}$ exists that takes as input the output of weak error mitigation $\mathcal{A}(\mathcal{C}, \mathcal{N}, \mathcal{M})$—the estimates $\{\hat{z}_b\}_{b\in\{0,1\}^m}$ with $|\hat{z}_b - \langle\psi| Z_b |\psi\rangle| < \tau$ for all b—and outputs $O(n)$-many samples from some distribution p where

$$d_{TV}(p, D_{|\psi\rangle}) \leq 1/16 \quad , \tag{133}$$

where $D_{|\psi\rangle}$ is the computational basis distribution on $|\psi\rangle$.

After we prove this, we will remark that not even the power to choose the observables *adaptively* will make it possible to transform a sub-exponential number of expectation values into linearly-many samples.

Proof of Theorem 9. Ref. [25] has shown that there is a class of n-qubit Clifford circuits whose output distributions are exactly the set of $\mathsf{PARITIES}$ distributions. Suppose to the contrary that for some circuit $\mathcal{C}$ in this class whose output distribution is some $P_s \in \mathsf{PARITIES}$, there exists some set of observables $\mathcal{M}$ with $|\mathcal{M}| = o(\tau^2 \cdot 2^n)$ and some algorithm $\mathcal{B}$, which takes as input the estimates $\hat{z}_i$ of weak error mitigation up to error τ and outputs $O(n)$ samples from some distribution p such that $d_{TV}(p, P_s) \leq 1/16$.

We will show that we can use these samples to solve for the hidden string and thus solve the problem of $\mathsf{PARITIES}$. However, this would imply a contradiction: Recall from Lemma 9 that each expectation value estimate is a statistical query to some distribution from the class of $\mathsf{PARITIES}$, but as stated in Lemma 7, there is no *statistical query* algorithm to solve $\mathsf{PARITIES}$ with $o(\tau^2 \cdot 2^n)$ statistical queries.

In the rest of this proof, we will explain how to use the samples output by the presumed $\mathcal{B}$ to solve $\mathsf{PARITIES}$. The key is simply to run the hypothesis selection algorithm of Ref. [27] (Theorem 8), with the set of candidate hypotheses being the set of all Clifford distributions encoding $\mathsf{PARITIES}$. This algorithm will take as input the $O(n)$ samples from p and by the guarantees of Theorem 8, will output a distribution $P_{s'} \in \mathsf{PARITIES}$ such that

$$d_{TV}(p, P_{s'}) \leq 3 \min_{\tilde{s}\in\{0,1\}^n} d_{TV}(p, P_{\tilde{s}}) + 1/16 \tag{134}$$

Now, consider that by the guarantees of $\mathcal{B}$, $3d_{TV}(p, P_s) + 1/16 = 1/4$ and so Eq. (134) yields that $d_{TV}(p, P_{s'}) < 1/4$. But as stated in Lemma 8, for all s, s',

$$d_{TV}(P_s, P_{s'}) \geq 1/2 \quad , \tag{135}$$

and so by the triangle inequality,

$$\arg \min_{\tilde{s}\in\{0,1\}^n} d_{TV}(p, P_{\tilde{s}}) = s \tag{136}$$

i.e., hypothesis selection recovers the hidden string of the $\mathsf{PARITIES}$ problem successfully. This implies the contradiction. □

B. Extensions of our proof

We remark that our bound holds against even an *adaptive* choice of observables in error mitigation because the statistical query hardness of $\mathsf{PARITIES}$ holds against an adaptive choice of statistical queries. However, we reiterate a limitation of our result: we can only rule out obtaining strong error mitigation from weak error mitigation with sub-exponentially-many *observables whose eigenbasis is the basis we desire*

to sample from. It is natural to ask if we can lift this restriction. Here, we point out that the discussion in Ref. [29] shows that for 'flat' circuit output distributions, and a specially-crafted set of observables with multiple different eigenbases, only polynomially-many expectation values are needed to obtain a sampler (hence we cannot lift the restriction in general). We could also take a different tack: our proof is based on the hardness of PARITIES from *classical* statistical queries.

As has been observed by Ref. [28], it is also possible to define *quantum* statistical queries for a given unknown distribution D, and unknown function c, and QSQs generalize classical statistical queries by allowing for observables not diagonal in the eigenbasis defined by c. Hence, if we knew of a problem that was hard for certain classes of *quantum* statistical queries (in the sense that exponentially-many QSQs are needed to solve them), our proof technique could then be applied to say something about the hardness of obtaining strong error mitigation from weak error mitigation without the restriction mentioned at the beginning of this paragraph. Unfortunately we do not know of any such problems.

---

[1] A. B. Tsybakov, *Introduction to non-parametric estimation* (Springer New York, 2009).
[2] W. J. Huggins, S. McArdle, T. E. O'Brien, J. Lee, N. C. Rubin, S. Boixo, K. B. Whaley, R. Babbush, and J. R. McClean, Virtual distillation for quantum error mitigation, Phys. Rev. X **11**, 041036 (2021).
[3] B. Koczor, Exponential error suppression for near-term quantum devices, Phys. Rev. X **11**, 031057 (2022).
[4] G. De Palma, M. Marvian, C. Rouzé, and D. Stilck Franca, Limitations of variational quantum algorithms: a quantum optimal transport approach (2022), arXiv:2204.03455.
[5] P. Czarnik, A. Arrasmith, P. J. Coles, and L. Cincio, Error mitigation with Clifford quantum-circuit data, Quantum **5**, 592 (2022).
[6] A. Lowe, M. H. Gordon, P. Czarnik, A. Arrasmith, P. J. Coles, and L. Cincio, Unified approach to data-driven quantum error mitigation, Phys. Rev. Res. **3**, 033098 (2021).
[7] A. Strikis, D. Qin, Y. Chen, S. C. Benjamin, and Y. Li, Learning-based quantum error mitigation, PRX Quantum **2**, 040330 (2021).
[8] K. Temme, S. Bravyi, and J. M. Gambetta, Error mitigation for short-depth quantum circuits, Phys. Rev. Lett. **119**, 180509 (2017).
[9] S. Endo, S. C. Benjamin, and Y. Li, Practical quantum error mitigation for near-future applications, Phys. Rev. X **8**, 031027 (2018).
[10] T. Giurgica-Tiron, Y. Hindy, R. LaRose, A. Mari, and W. J. Zeng, Digital zero noise extrapolation for quantum error mitigation, in *2020 IEEE International Conference on Quantum Computing and Engineering (QCE)* (2020) pp. 306–316, 2005.10921.
[11] Z. Cai, Multi-exponential error extrapolation and combining error mitigation techniques for NISQ applications, npj Quant. Inf. **7**, 80 (2021), 2007.01265.
[12] Y. Li and S. C. Benjamin, Efficient variational quantum simulator incorporating active error minimization, Phys. Rev. X **7**, 021050 (2017).
[13] E. van den Berg, Z. K. Minev, A. Kandala, and K. Temme, Probabilistic error cancellation with sparse Pauli-Lindblad models on noisy quantum processors, Nature Physics **19**, 1116 (2023), 2201.09866.
[14] R. Takagi, S. Endo, S. Minagawa, and M. Gu, Fundamental limits of quantum error mitigation, npj Quant. Inf. **8**, 114 (2022).
[15] K. Tsubouchi, T. Sagawa, and N. Yoshioka, Universal cost bound of quantum error mitigation based on quantum estimation theory (2022), arXiv:2208.09385.
[16] R. Takagi, H. Tajima, and M. Gu, Universal sample lower bounds for quantum error mitigation (2022), arXiv:2208.09178.
[17] C. Hirche, C. Rouzé, and D. Stilck França, On contraction coefficients, partial orders and approximation of capacities for quantum channels, Quantum **6**, 862 (2022).
[18] R. Cleve, D. Leung, L. Liu, and C. Wang, Near-linear constructions of exact unitary 2-designs, Quant. Inf. Comp. **16**, 721–756 (2016).
[19] R. Beals, S. Brierley, O. Gray, A. W. Harrow, S. Kutin, N. Linden, D. Shepherd, and M. Stather, Efficient distributed quantum computing, Proc. Roy. Soc. A **469**, 20120686 (2013).
[20] S. Brierley, Efficient implementation of quantum circuits with limited qubit interactions, Quant. Inf. Comp. **17**, 1096–1104 (2017).
[21] J. Watrous, *The theory of quantum information* (Cambridge University Press, 2018).
[22] A. S. Holevo, Statistical decision theory for quantum systems, J. Multiv. Ana. **3**, 337 (1973).
[23] C. W. Helstrom, Quantum detection and estimation theory, J. Stat. Phys. **1**, 231 (1969).
[24] M. Kearns, Efficient noise-tolerant learning from statistical queries, J. ACM **45**, 983–1006 (1998).
[25] M. Hinsche, M. Ioannou, A. Nietner, J. Haferkamp, Y. Quek, D. Hangleiter, J.-P. Seifert, J. Eisert, and R. Sweke, A single T-gate makes distribution learning hard, Phys. Rev. Lett. **130**, 240602 (2023).
[26] A. Blum, M. Furst, J. Jackson, M. Kearns, Y. Mansour, and S. Rudich, Weakly learning DNF and characterizing statistical query learning using Fourier analysis, in *Proceedings of the Twenty-Sixth Annual ACM Symposium on Theory of Computing*, STOC '94 (Association for Computing Machinery, New York, NY, USA, 1994) p. 253–262.
[27] Y. G. Yatracos, Rates of convergence of minimum distance estimators and Kolmogorov's entropy, Ann. Stat. **13**, 768 (1985).
[28] S. Arunachalam, A. B. Grilo, and H. Yuen, Quantum statistical query learning, arXiv:2002.08240 (2020).
[29] D. S. França and R. Garcia-Patron, A game of quantum advantage: Linking verification and simulation, Quantum **6**, 753 (2022).
